# Association of Circulating Vascular Endothelial Growth Factor Levels With Autoimmune Diseases: A Systematic Review and Meta-Analysis

**DOI:** 10.3389/fimmu.2021.674343

**Published:** 2021-05-27

**Authors:** Haoting Zhan, Haolong Li, Chenxi Liu, Linlin Cheng, Songxin Yan, Yongzhe Li

**Affiliations:** ^1^ Department of Clinical Laboratory, Peking Union Medical College Hospital, Peking Union Medical College and Chinese Academy of Medical Sciences, Beijing, China; ^2^ Department, State Key Laboratory of Complex, Severe and Rare Diseases, Peking Union Medical College Hospital, Chinese Academy of Medical Science and Peking Union Medical College, Beijing, China

**Keywords:** diagnosis, disease activity, autoimmune disease, vascular endothelial growth factor, angiogenesis

## Abstract

**Background:**

Autoimmune diseases (ADs) are characterized by immune-mediated tissue damage, in which angiogenesis is a prominent pathogenic mechanism. Vascular endothelial growth factor (VEGF), an angiogenesis modulator, is significantly elevated in several ADs including rheumatoid arthritis (RA), systemic sclerosis (SSc), and systemic lupus erythematosus (SLE). We determined whether circulating VEGF levels were associated with ADs based on pooled evidence.

**Methods:**

The analyses included 165 studies from the PubMed, EMBASE, Cochrane Library, and Web of Science databases and fulfilled the study criteria. Comparisons of circulating VEGF levels between patients with ADs and healthy controls were performed by determining pooled standard mean differences (SMDs) with 95% confidence intervals (CIs) in a random-effect model using STATA 16.0. Subgroup, sensitivity, and meta-regression analyses were performed to determine heterogeneity and to test robustness.

**Results:**

Compared with healthy subjects, circulating VEGF levels were significantly higher in patients with SLE (SMD 0.84, 95% CI 0.25–1.44, *P* = 0.0056), RA (SMD 1.48, 95% CI 0.82–2.15, *P <*0.0001), SSc (SMD 0.56, 95% CI 0.36–0.75, *P <*0.0001), Behcet’s disease (SMD 1.65, 95% CI 0.88–2.41, P <0.0001), Kawasaki disease (SMD 2.41, 95% CI 0.10–4.72, P = 0.0406), ankylosing spondylitis (SMD 0.78, 95% CI 0.23–1.33, P = 0.0052), inflammatory bowel disease (SMD 0.57, 95% CI 0.43–0.71, P <0.0001), psoriasis (SMD 0.98, 95% CI 0.62–1.34, P <0.0001), and Graves’ disease (SMD 0.69, 95% CI 0.20–1.19, P = 0.0056). Circulating VEGF levels correlated with disease activity and hematological parameters in ADs.

**Conclusion:**

Circulating VEGF levels were associated with ADs and could predict disease manifestations, severity and activity in patients with ADs.

**Systematic Review Registration:**

PROSPERO, identifier CRD42021227843.

## Introduction

Angiogenesis, a hallmark of inflammatory activation, is an integral part of pathogenic processes including endothelial cell proliferation and migration and subsequent neoangiogenesis and remodeling in autoimmune diseases (ADs). Synovial pannus initiates the invasion of cartilage and subchondral bone to perpetuate rheumatoid arthritis (RA) (1, 2), whereas ankylosing spondylitis (AS) is characterized by increased vascularity and vascular lesions (3). Vascular endothelial dysfunction and injury are considered as the primum movens triggering Kawasaki disease (KD), systemic lupus erythematosus (SLE), inflammatory bowel disease (IBD), Behcet’s disease (BD), systemic sclerosis (SSc), and psoriasis (PsA) (4–9). Therefore, early detection of vascular involvement is pivotal in AD diagnosis.

Vascular endothelial growth factor (VEGF)-A, generally known as VEGF, is a crucial regulator of endothelial dysfunction, capillary permeability, and angiogenesis. For example, serum VEGF level and intrathyroid microvessel density were reported to be increased patients with Graves’ disease (GD) compared to healthy control (HC) subjects (10). Increased serum VEGF and significant difference in diffused and limited SSc suggest VEGF as a potential surrogate indicator of capillary damage (11). Strong VEGF expression in synovial fluid and serum of patients with RA was shown to lead to synovial neovascularization and destruction in cartilage and bones (12, 13). VEGF was reported to be overexpressed in the skin and peripheral blood of patients with PsA (14). Serum VEGF levels were shown to be elevated and to correlate with disease activity and severity in PsA, SLE, BD, IBD, KD, and AS (14–19). These findings suggest VEGF as a potential pathogenic factor with promising diagnostic value in ADs. However, no clinical guidelines currently recommend serum VEGF evaluation in routine care and counseling of patients with ADs, and intensive studies are warranted to identify the clinical implications of the findings regarding VEGF’s role in ADs to date and to resolve contradictory results (20–24).

Given the inconsistency among these findings and lower statistic power of the studies, we performed a systematic review and meta-analysis to generate independent results and recognize the source of heterogeneity. In the present study, we aimed to determine whether circulating VEGF was a causative factor in ADs.

## Materials and Methods

### Literature Search

The present systematic review with meta-analysis was performed according to the PRISMA guidelines (PROSPERO registration number, CRD42021227843). Two authors (HTZ and HLL) independently searched the PubMed, Embase, Cochrane Library, and the Web of Science databases for studies published until October 14. The detailed search strategies are provided in the online **Supplemental Materials**. Reference lists were manually retrieved.

### Eligibility Criteria

Without restrictions on time, language, ethnicity, and geographical region, studies satisfying the following criteria were included: (1) case-control or cohort studies on the association between circulating VEGF and ADs including SLE, RA, SSc, BD, KD, AS, IBD, PsA, and GD; (2) HCs without ADs (2); available data on circulating VEGF levels (serum or plasma); (3) sufficient data on VEGF levels for both HCs and patients with ADs to evaluate standard mean differences (SMDs) with 95% confidence intervals (CIs). Studies based on animal and cellular models, those comprising HCs with insufficient data; and editorial letters with insufficient data were excluded.

### Data Extraction and Quality Assessment

Two independent investigators (HTZ and HLL) individually screened the literature and extracted and evaluated the data. Any discrepancies were resolved by consensus or by a third opinion (YZL). Study number, name of the first author, publication year, country, study type, sample type, inclusion and exclusion criteria, demographic features, aggregated number of subjects and circulating VEGF levels in patients with ADs and HCs, diagnostic criteria, type of VEGF assay, and treatment history and strategy were extracted into pre-designed charts. For meta-analysis, continuous variables were translated from medians (interquartile range [IQR] or range) to means ± standard deviation (25). Newcastle–Ottawa quality assessment scale was used to evaluate study quality. Further details of the pooled studies were obtained by directly contacting the authors if warranted.

### Data Analysis

STATA V.16.0 was used to perform the meta-analysis. SMDs with 95% CIs were used to estimate the pooled results and compare circulating VEGF levels between patients and HC groups. Random-effect model was used for analysis. Significant heterogeneity was ascertained based on a *p* value of ≤0.10 using the Cochrane Q test or an I^2^ value of >50%. Subgroup, sensitivity, and meta-regression analyses were performed to identify the source of heterogeneity and to test robustness. Spearman correlation coefficients were transformed into Pearson’s r values, which were converted to Fisher’s z values to obtain approximately normal distributions. Ultimately, the summary Fisher’s z values were converted into summary r values. Summary r values of 0.8–1.0, 0.6–0.8, 0.4–0.6, and 0.2–0.4 indicated extreme, high, and moderate relevance and poor correlation, respectively (details provided in the online **Supplemental Materials**). Publication bias was assessed by Egger’s linear regression test and contour-enhanced funnel plots with collaborative meta-trim. A two-sided *P <*0.05 was considered to indicate statistical significance.

## Results

### Search Results and Population Characteristics

**Figure 1:**
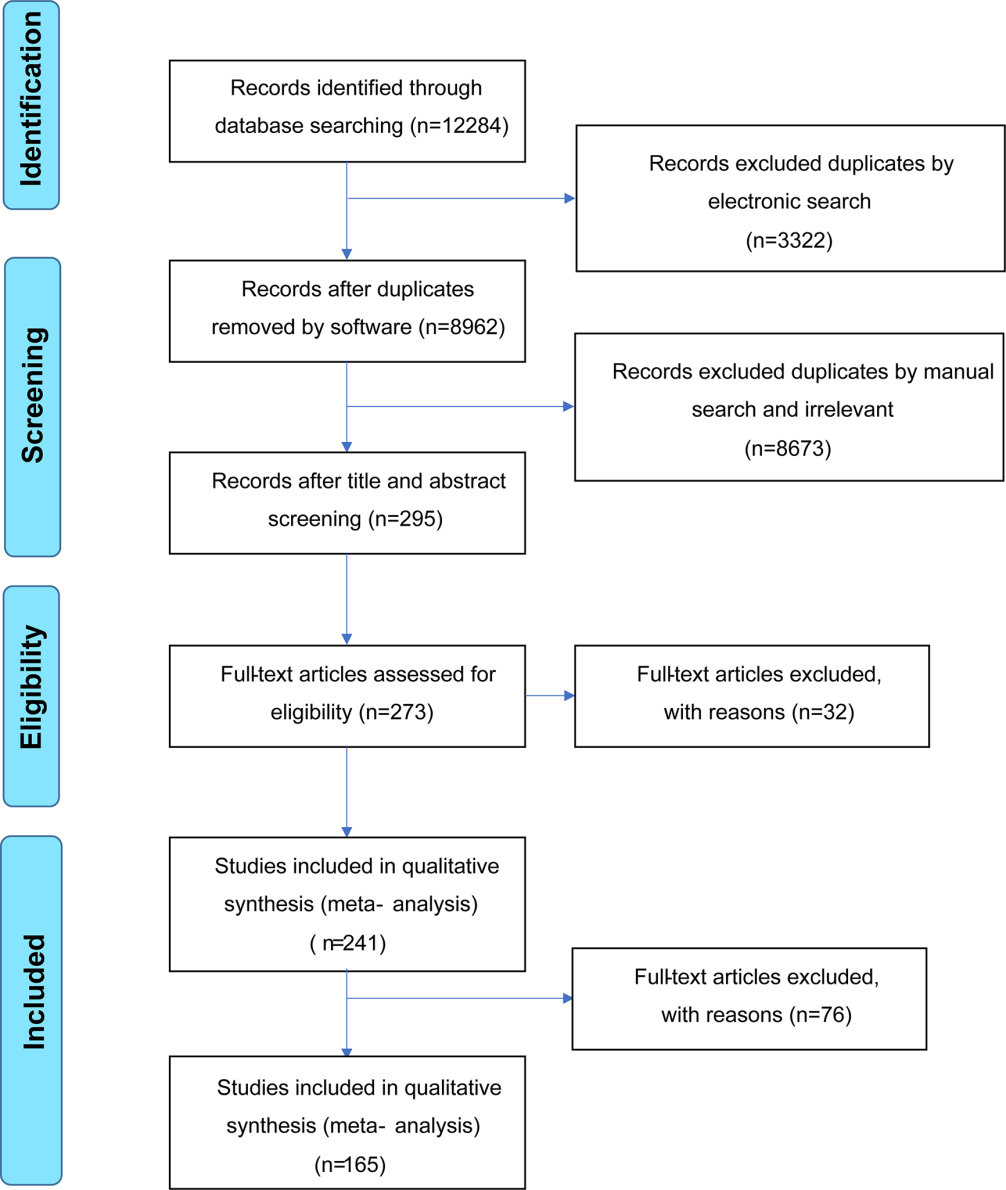
Flow diagram of included/excluded studies.

The literature search is summarized in **Figure 1**. After removing duplicate studies (n = 3,322) and irrelevant publications (n = 8,673), 298 articles were analyzed and the full texts of 273 articles were read. Thirty-two full-text articles were eliminated due to incomplete data or unrelated outcomes. Among 241 eligible studies meeting the inclusion criteria, 76 articles were excluded due to unextractable data, insufficient data on HCs, irrelevant VEGF sample type (urine/synovial fluid/tear fluid), or inappropriate disease control groups. Finally, 165 studies were included in the meta-analysis, with 28, 29, 40, 13, 8, 12, 16, 23, and six studies on SLE (20, 21, 26–51), RA (12, 22–24, 38, 43, 52–74), SSc (11, 38, 39, 64, 75–110), BD (111–123), KD (18, 124–130), AS (55, 73, 131–140), IBD (141–156), PsA (12, 14, 135, 136, 157–175) and GD (10, 176–180), respectively. The main study characteristics are summarized in **Table 1** and **Appendix 1**. The studies were medium-to-high quality based on the Newcastle–Ottawa quality assessment scale scores (range, 4–9).

**Table 1:** Population characteristics of the studies included in the meta-analysis.

Year	Author		Country	Study type	SLE			HC		
					Sample size	Female (%)	Age (years)	Sample size	Female (%)	Age (years)
2015	Barbulescu AL (20)		Romania	case-control	18	16 (88.88)	45.00 ± 10.81	17	16 (94.11)	range: 19–64
2019	Barraclough M (21)		UK	case-control	36	34 (94)	40 ± 12.41	30	30 (100)	32 ± 14.44
2008	Ciprandi G (26)		Italy	case-control	40	40 (100)	41.95 ± 8.3	40	33 (82.5)	43 ± 8.2
2009	Colombo BM (27)		Italy	case-control	80	80 (100)	42.6 ± 9.1	80	80 (100)	40.1 ± 9.5
2014	De Jesus GR (28)		Brazil	case-control	54	54 (100)		34	34 (100)	
2015	Ding Y (29)		China	case-control	41	30 (73.2)	11.1 ± 2.4	10		
2009	Elhelaly NS (30)		Egypt	case-control	23	21 (91.3)	Range 8–18	25		
2012	Edelbauer M (31)		Austria	case-control	23	17 (73.9)	15 ± 5	20	5 (25)	12 ± 3
2018	El-Gazzar II (32)		Egypt	case-control	84	84 (100)	29.03 ± 5.4	33		
2017	Ghazali WSW (33)		Malaysia	case-control	92			26	26 (100)	33.19 ± 10.3
					LN 46	44 (96)	28.48 ± 9.93			
					Non-LN 46	46 (100)	32.39 ± 11.46			
2007	Heshmat NM (34)		Egypt	case-control	25	24 (96)	14.1 ± 2.6	30	29 (96.7)	14.0 ± 2.5
									
2009	Hrycek A (35)		Poland	case-control	48	48 (100)	47 ± 14	24	24 (100)	51 ± 15
2009	Hrycek A (36)		Poland	case-control	21	21 (100)	51 ± 12.4	24	24 (100)	51 ± 15.3
2008	Ibrahim FF (37)		Egypt	case-control	30	30 (100)	25 ± 7.75	10	10 (100)	32 ± 7.5
1998	Kikuchi K (38)		Japan	case-control	17	14 (82.4)	47 ± 12.75	20	16 (80%)	50 ± 12.5
2013	Koca SS (39)		Turkey	case-control	23	21 (91.3)	37.9 ± 9.3	28	22 (78.6%)	42.5 ± 13.9
2007	Kuryliszyn-Moskal A (40)		Poland	case-control	47	44 (93.6)	40.8 ± 13.6	30		
2014	Liu J (41)		China	case-control	75	59 (78.7)	35.42 ± 11.79	40	31 (77.5)	33.62 ± 10.21
2018	Merayo-Chalico J (42)		Mexico	case-control	active SLE 6	6 (100)	34.6 ± 4.2	6	6 (100)	36 ± 4.1
					remission SLE 6	6 (100)	34.1 ± 4.8			
2016	Novikov A (43)		Russia	case-control	80	72 (90)	31.5 ± 36.3	28		
2012	Moneib HA (44)		Egypt	case-control	30	21 (70)	28.9 ± 10.2	15	10 (66)	35.00 ± 9.48
2002	Navarro C (45)		Mexico	case-control	28	24 (85.7)	36.6± 16.1	24	19 (79.2)	29.2 ± 8.5
									
2005	Robak E (46)		Poland	case-control	41	38 (92.7)	40.5 ± 13.5	20		
2003	Robak E (47)		Poland	case-control	60	55 (91.7)	41 ± 14.25	20	17 (85)	45 ± 5.75
2013	Robak E (48)		Poland	case-control	60	56 (93.3)	39.2 ± 11.25	20	17 (85)	
2002	Robak E (49)		Poland	case-control	52	48 (92.3)	41 ± 14.75	20	18 (90)	38 ± 11.75
2017	Willis R (50)		America	case-control	312			30	83.3	43.5 ± 12.5
				cohort1	267	252 (94.4)	47.6 ± 12.4			
				cohort2	45	44 (97.8)	44.0 ± 12.1			
2014	Zhou L (51)		China	case-control	54	50 (92.6)	36.81 ± 12.52	28	22 (78.6)	37.82 ± 12.86
**Year**	**Author**		**Country**	**Study type**	**RA**			**HC**		
					**Sample size**	**Female (%)**	**Age (years)**	**Sample size**	**Female (%)**	**Age (years)**
2004	Ardicoglu O (52)			case-conrol	38			40		
2001	Ballara S (12)		UK	cohort	early 44	61	61 ± 17.78	31	65	49 ± 12.59
					longstanding 78	85	61 ± 14.07			
2000	Bottomley MJ (53)		UK	case-conrol	61	51 (83.6)	59 ± 11.75	29	20 (69.0)	34 ± 8
2005	Kim HR (62)		Korea	case-conrol	30	24 (80)	50 ± 8	20	16 (80)	30 ± 8
2016	Deveci K (55)		Turkey	case-control	30		mean age of 30–50	30		mean age of 30–50
2002	Drouart M (56)		France	case-control	50	32 (64)	59.8 ± 12.8	64	30 (46.9)	42.1 ± 10.1
2016	do Prado AD (57)		Brazil	case-control	64	50 (78.1)	55.3 ± 9.8	30	23 (76.7)	55.9 ± 11.1
2009	Foster W (22)		UK	case-control	66	41 (62.1)	58 ± 14	49	34 (69.4)	54 ± 10
2018	Gumus A (58)		Turkey	case-control	59			25	20 (80.0)	46.4 ± 13.3
					joint swelling (+) 31	27 (87.10)	45.06 ± 9.66			
					joint swelling (−) 28	25 (89.28)	45.10 ± 13.03			
2014	Heard BJ (59)		Canada	case-control	100		46.5 ± 14.5	100		40.0 + 9.5
2008	Hetland ML (60)		Denmark	case-control	10			10		
2003	Hashimoto N (61)		Japan	case-control	active RA22	18 (81.8)	54 ± 12.75	11		
1998	Kikuchi K (38)		Japan	case-control	11	10 (90.9)	51 ± 10.75	20	16 (80)	50 ± 12.5
2007	Cho ML (54)		Korea	case-control	72		49.6 ± 1.3	31		47.1 ± 2.1
2006	Kuryliszyn-Moskal A (63)		Poland	case-control	64	54 (84.4)	58.6 ± 12.6	32		
2004	Kuwana M (64)		Japan	case-control	11	11 (100)	59.1 ± 12.0	11	11 (100)	52.7 ± 10.6
2010	Milman N (65)		Canada	case-control	47	78.70	54.3 ± 14.25			
2018	Misra S (23)		India	case-control	50	46 (92)	35.90 ± 18.607	30	28 (93.3)	34.03 ± 10.3
2016	Novikov A (43)		Russia	case-control	74	59 (79.7)	54.0 ± 13.33			
2001	Olszewski WL (66)		Poland	case-control	20	16 (80)	42 ± 7.5	20		25 ± 1
2012	Oranskiy SP (67)		Russia	case-control	39 (BMI normal)	82.0	53.0 ± 2.75	20	80.0	52.0 ± 2.5
2010	Ozgonenel L (68)		Turkey	case-control	40	32 (80)	46 ± 12.59	38	18 (47.4)	44 ± 11.11
									
2009	Young HR (69)		America	case-control	169	69.20	54.2 ± 11.8	92	63	53.2 ± 11.6
									
2016	Rodriguez-Carrio J (70)		Spain	case-control	212	175 (82.5)	54 ± 17.25	175	102 (58.3)	51 ± 14.25
2016	Smets P (71)		France	case-control	80:RA13	8 (61.5)	71 ± 7.97	37	24 (64.9)	73.35 ± 8.55
									
2004	Strunk J (72)		Germany	case-control	active RA 21	16 (76.2)	range: 38–79	12	6 (50)	range: 17–58
2010	Tseng JC (73)		China	case-control	50			50		
2001	Sone H (24)		Japan	case-control	155	130 (83.9)	57.9 ± 12.0	75	62 (82.7)	55.8 ± 15.4
2007	Zayed A (74)		Egypt	case-control	40		range:21–57	20		
**Year**	**Author**		**Country**	**Study type**	**SSc**			**HC**		
					**Sample size**	**Female (%)**	**Age (years)**	**Sample size**	**Female (%)**	**Age (years)**
2018	Alekperov R (75)		Russia	case-control	46			20		
2004	Allanore Y (76)		France	case-control	40	33 (82.5)	57 ± 12	20	17 (85)	51 ± 7
2013	Aydogdu E (77)		Turkey	case-control	40	38 (95)	48.35 ± 13.2	20	19 (95)	49.3 ± 8.5
2017	Benyamine A (78)		France	case-control	45	44 (97.8)	61.49 ± 11.95	41	38 (92.7)	56.09 ± 7.82
2014	Bosello SL (79)		Italy	case-control	28			11		
2014	Bosello SL (80)		Italy	case-control	24			10		
2002	Choi JJ (11)		Korea	case-control	48	45 (81.8)	40.6 ± 13	55	30	38 ± 6
2017	Chora I (81)		Italy	case-control	55	49 (89.0)	64 ± 11	55	51 (92.7)	52 ± 10.25
					VEDOSS 25	21 (84.0)	50 ± 14.5			
2016	Cossu M (82)		Italy	case-control	UCTD/SSC 47	52.7 ± 14.2		43		
					SSc without skin fibrisis 48	62 ± 13.2				
					limited 51	62.1 ± 10.4				
					diffused 35	54.6 ± 12.6				
2013	De Lauretis A (83)		UK	case-control	74	59 (79.7)	51.4 ± 12.1	20	7 (35)	32.7 ± 6.3
2017	Delle Sedie A (84)		Italy	case-control	41	40 (97.6)	56 ± 15	31	25 (80.6)	50 ± 16
2011	Distler JHW (85)		Germany	case-control	40	34 (85)	46 ± 14.5	66	44 (66.7)	39 ± 13.75
2002	Distler O (86)		Italy	case-control	43	35 (81.4)	61 ± 13.75	21	16 (76.2)	55 ± 16.75
2012	Dunne JV (87)		Canada	case-control	40	35 (87.5)		40		
					diffused 14	45.5 ± 9.5				
					limited 26	53.8 ± 13.25				
2005	Dziankowska-Bartkowiak B (88)		Poland	case-control	34	26 (76.5)	48 ± 13.5	20	19 (95.0)	46 ± 9.75
					diffused 15	8 (53.3)	45 ± 12			
					limited 19	18 (94.7)	50 ± 10.75			
2006	Dziankowska-Bartkowiak B (89)		Poland	case-control	28	22 (78.6)	47.5 ± 13	20	15 (75)	46 ± 9.75
					diffused 12	7 (58.3)	48 ± 11.5			
					limited 16	15 (93.8)	47 ± 10.75			
2013	Farouk HM (90)		Egypt	case-control	25	21 (84)	40.3 ± 5.86	20	17 (85)	38.9 ± 3.8
2014	Gkodkowska-Mrowka E (91)		Poland	case-control	66	60 (90)	53 ± 13.25	21	18 (85.7)	52 ± 10.25
2018	Gigante A (92)		Italy	case-control	15	15 (100)	41 ± 10.835	10		39 ± 10.484
2008	Hummers LK (93)		America	case-control	113	88.90	53.0 ± 12.2	27	63	57.5 ± 2.8
2017	Ibrahim SE (94)		Egypt	case-control	35	33 (94.2)	30.43 ± 4.53	35		29.8 ± 4.03
2018	Kawashiri S (95)		Japan	case-control	60	56 (93.3)	64 ± 8.889	25		
					diffused 16	15 (93.8)	64 ± 6.667			
					limitted 44	41 (93.2)	64 ± 10.37			
1998	Kikuchi K (38)		Japan	case-control	40	37 (92.5)	53 ± 16.25	20	16 (80)	50 ± 12.5
2004	Kuryliszyn-Moskal A (96)		Poland	case-control	31	31 (100)	55.2 ± 10.4	30		
2013	Koca SS (39)		Turkey	case-control	37	32 (86.5)	45.7 ± 13.6	28	22 (78.6)	42.5 ± 13.9
2020	Lv TT (97)		China	case-control	30	18 (75)	44 ± 12.0	15		
2004	Kuwana M (64)		Japan	case-control	11	11 (100)	57.7 ± 11.8	11	11 (100)	52.7 ± 10.6
2019	Michalska-Jakubus M (98)		Poland	case-control	47	47 (100)	56.43 ± 11.01	27	27 (100)	52.37 ± 8.87
2010	Minier T (99)		Hungary	case-control	131	90.80	55.9 ± 11.7	30		
					diffused 41	82.80	52.6 ± 13.8			
					limited 90	94.40	57.4 ± 10.3			
2012	Morgiel E (100)		Poland	case-control	30	26 (86.7)	54 ± 10.3	20		
2009	Papaioannou AI (101)		Greece	case-control	40	33 (82.5)	56.75 ± 12.5	13		
2015	Reiseter S (102)		Norway	cohort	298	243 (82)	56.0 ± 13.8	100		
2001	Sato S (103)		Japan	case-control	32	29 (90.6)	47 ± 18	20		
2010	Riccieri V (104)		Italy	case-control	65	63 (96.9)	57.3 ± 15.25	16		
2017	Saranya C (105)		India	case-control	55		median 38	30		median 39
2016	Shenavandeh S (106)		Iran	case-control	44	40 (90.9)	40.7 ± 12.8	44	41 (93.2)	39.4 ± 11.76
2009	Solanilla A (107)		France	case-control	35			25		
2016	Yalcinkaya Y (108)		Turkey	case-control	72	66 (92)	44.9 ± 12.7	20		
2020	Waszczykowska A		Poland	case-control	25	21 (84)	57.1 ± 10.8	25	20 (80)	59.4 ± 9.9
	(109)				diffused 8	7 (87.5)	50.6 ± 11.4			
					limited 17	14 (82.4)	60.2 ± 9.4			
2008	Wipff J (110)		France	case-control	187	157 (84)	55.9 ± 13.2	48	40 (83.3)	59.4 ± 11.6
**Year**	**Author**		**Country**	**Study type**	**BD**			**HC**		
					**Sample size**	**Female (%)**	**Age (years)**	**Sample size**	**Female (%)**	**Age (years)**
2018	Arica DA (111)		Turkey	case-control	45	22 (48.9)	36.7 ± 10.3	28		35.7 ± 7.51
2003	Cekmen M (112)		Turkey	case-control	39	18 (46.2)	38.1 ± 10.4	15	7 (46.7)	39.2 ± 9.3
2013	Eldin AB (113)		Egypt	case-control	30	6 (20)	30.6 ± 9.36	20	4 (20)	26.9 ± 8.38
2003	Erdem F (114)		Turkey	case-control	33	16 (48.5)	33.2 ± 10.4	30	9 (30)	34.0 ± 11.1
2012	Ganeb SS (115)		Egypt	case-control	70	27 (38.6)	32.84 ± 3.63	70	29 (41.4)	32.81 ± 3.89
2019	Gheita TA (116)		Egypt	case-control	96		34.9 ± 10.1	60	9 (25)	36.7 ± 12.6
					active 59	11 (18.6)	33.03 ± 9.8			
					inactive 37	6 (16.2)	36.2 ± 10.1			
2011	Ibrahim SE (117)		Egypt	case-control	40	8 (20)	40.35 ± 7.34	40	9 (22.5)	37.3 ± 7.06
2017	Kul A (118)		Turkey	case-control	active 40	16 (40)	37.6 ± 8.7	40	18 (45)	38.8 ± 7.9
2009	Ozdamar Y (119)		Turkey	case-control	active prosterior segment of BD 20	7 (35)	33 ± 6			
					inactive ocular BD 23	10 (43.5)	35 ± 7			
2007	Ozturk MA (120)		Turkey	case-control	21	6 (28.6)	35.8 ± 8.6	21		
2018	Sertoglu E (121)		Turkey	case-control	55	18 (32.7)	40 ± 10	31	12 (38.7)	40 ± 13
2006	Shaker O (122)		Egypt	case-control	30	20	32.6 ± 9.14	15	20	30.13 ± 12.32
2013	Yalcindag A (123)		Turkey	case-control	65	32 (49)	40.3 ± 9.8	21	11 (48)	38.5 ± 9.3
**Year**	**Author**		**Country**	**Study type**	**KD**			**HC**		
					**Sample size**	**Female (%)**	**Age (years)**	**Sample size**	**Female (%)**	**Age (years)**
2011	Breunis WB (124)		Netherlands	case-control	early101			18		
2001	Hamamichi Y (125)		Japan	case-control	acute 49		1.9 ± 0.2	38		4.5 ± 0.7
					convalesent 30		4.8 ± 0.7			
1998	Maeno N (126)		Japan	case-control	22	10 (45.5)	2.2 ± 1.425	healthy 19	9 (47.7)	1.4 ± 1.4
					acute 20	10 (50)	1.5 ± 1.15	febrile 22	10 (45.5)	1.3 ± 1.4
					subacute 13	5 (38.5)	2.5 ± 1.325			
					convalesent 15	8 (53.3)	1.9 ± 1.4			
1999	Ohno T (18)		Japan	case-control	acute 66	24 (36.4)	1.79 ± 2.375	healthy 18	8 (44.4)	4.25 ± 1.75
					acute phase31			febrile 18	9 (50)	3.375 ± 2.29
					convalescent phase31					
2002	Takuro Ohno (127)		Japan	case-control	acute phase 41	14 (34.1)	1.83 ± 2.17	25	8 (32)	9 ± 1.75
					convalescent phase 41					
2019	Su Y (128)		China	case-control	90	51 (56.7)	2.55 ± 1.72	healthy 60	28 (46.7)	2.19 ± 2.22
								febrile 40	20 (50)	2.84 ± 1.63
2009	Ueno K (129)		Japan	case-control	80	37 (46.25)	2.1 ± 1.8	febrile 26	10 (38.5)	1.9 ± 1.1
2016	Zeng H (130)		China	case-control	52					
**Year**	**Author**		**Country**	**Study type**	**AS**			**HC**		
					**Sample size**	**Female (%)**	**Age (years)**	**Sample size**	**Female (%)**	**Age (years)**
2016	Akar S (13)]		Turkey	case-control	98		27.7 ± 8.6	49		
2016	Deveci K (55)		Turkey	case-control	30		mean age of 30–50	30		mean age of 30–50
2002	Goldberger C (132)		Austria	case-control	16	2 (12.5)	50.4 ± 2.7	8		
2015	Lin TT (133)		China	case-control	140	102 (72.9)	31.8 ± 9.3	90	72 (80)	30.2 ± 8.2
2016	Przepiera-Bedzak H (134)		Poland	case-control	80	16 (20)	50.9 ± 12.8	21	8 (38.1)	48.2 ± 13.5
2015	Przepiera-Bedzak H (135)		Poland	case-control	61	12 (19.7)	43.3 ± 13.2	29	19 (65.5)	48.2 ± 13.5
2016	Przepiera-Bedzak H (136)		Poland	case-control	81	20 (24.7)	44.7 ± 13.2	30	19 (63.3)	43.5 ± 9.4
2016	Sakellariou GT (137)		Greece	case-control	57	4 (7.0)	39.1 ± 1.4	34	2 (6.0)	38.8 ± 1.0
2015	Solmaz D (138)		Turkey	case-control	98	21 (21.4)	39.3 ± 10.0	49	12 (24.5)	39.0 ± 5.9
									
2018	Solmaz D (139)		Turkey	case-control	97	21 (21.6)	38 ± 10.4	48	12 (25)	41 ± 5.0
2019	Torres L (140)		Sweden	case-control	204	87 (43)	49 ± 15.56	80		
2010	Tseng JC (73)		China	case-control	50			50		
**Year**	**Author**		**Country**	**Study type**	**IBD**			**HC**		
					**Sample size**	**Female (%)**	**Age (years)**	**Sample size**	**Female (%)**	**Age (years)**
2018	Aksoy EK (141)		Turkey	case-control	UC 39	15 (38.5)	46.1 ± 12.6	15	7 (46.7)	41.4 ± 12.6
2014	Algaba A (142)		Spain	case-control	37 (UC = 6)	20 (54)	36 ± 13	40	24 (60)	43 ± 9
2004	Di Sabatino A (143)		Italy	case-control	CD 25		37.8 ± 11.25	22		38.3 ± 11.25
2007	Dueñas Pousa I (144)		Spain	case-control	CD 30	15 (50)	44 ± 14	30	15 (50)	43 ± 14
2006	Ferrante M (145)		Belgium	cohort	824	466 (56.6)	38.9 ± 12.07	271	156 (57.6)	28 ± 10.37
1999	Griga T (146)		Germany	case-control	27			10	5 (50)	29.3 ± 6.1
					CD 19	8 (42.1)	34.8 ± 11.0			
					UC 8	3 (37.5)	46.6 ± 19.5			
1998	Griga T (147)		Germany	case-control	46			9	5 (55.6)	31.5 ± 8.0
					CD 31	13 (41.9)	33.1 ± 7.9			
					UC 15	7 (46.7)	34.5 ± 12.0			
2001	Kanazawa S (148)		Japan	case-control	22			20	12 (60)	60 ± 8
					CD 11	7 (63.6)	38.5 ± 5.75			
					UC 11	6 (54.5)	56.5 ± 10.75			
2003	Kapsoritakis A (149)		Greece	case-control	94			23		38 ± 9
					CD 44					
					UC 50					
2015	Kleiner G (150)		Italy	case-control	26;CD15;UC11	12 (46.2)	9 ± 3.75	37	22 (59.5)	11 ± 4
2004	Magro F (151)		Portugal	case-control	218			115	59 (51.3)	32 ± 9.75
					CD 145	84 (57.9)	33 ± 14.5			
					UC 73	43 (58.9)	35 ± 11.75			
2011	Pousa ID (152)		Spain	case-control	active UC 13	46	46 ± 12	26		
2007	Pousa ID (153)		Spain	case-control	CD 70	39 (55.7)	42 ± 13	30	15 (50)	43 ± 14
1997	Schurer-Maly CC (154)		Switzer-land	case-control	CD 24			32		
					UC 23					
2020	deZoeten EF (155)		America	case-control	pediatric	5/18 (27.8)	12.7 ± 12.7	pediatric 17	7/18 (38.9)	12.7 ± 16.5
					active IBD 17					
					adult			adult 19	7/19 (36.8)	56.9 ± 14.4
					actuve UC 10	36.4 ± 11.7				
					inactive UC 10	52.6 ± 17.7				
2007	Wiercinska-Drapalo A (156)		Poland	case-control	UC 33	13 (39.4)	43 ± 12.75	20	5 (25)	38 ± 6
**Year**	**Author**		**Country**	**Study type**	**PsA**			**HC**		
					**Sample size**	**Female (%)**	**Age (years)**	**Sample size**	**Female (%)**	**Age (years)**
2009	Ablin JN (14)	Israel		case-control	skin10	4 (40)	48.6 ± 18.6	16	12 (75)	41.69 ± 9.71
					arthritis22	10 (45.5)	47.18 ± 8.15			
2007	Akman A (157)	Turkey		case-control	46	30 (65.2)	43.2 ± 14.4	20	7 (35)	34.6 ± 14.5
2010	Anderson KS (158)	Sweden		case-control	plaque(PV) 14	4 (28.6)	47 ± 10.75	14		
2001	Ballara S (12)	UK		cohort	arthritis13	62	46 ± 17.04	31	65	49 ± 12.59
2016	Batycka-Baran A (159)	Poland		case-control	arthritis 24	37.5	48.29 ± 9.05	36		
									
2012	Batycka-Baran A (160)	Poland		case-control	plaque-type psoriasis 63	41.3	42.16 ± 15.42	31	48.4	41.35 ± 15.23
2016	Capkin AA (161)	Turkey		case-control	48	16 (33.3)	48.6 ± 12.5	48	20 (41.7)	52.3 ± 8.4
1999	Bhushan M (162)	UK		case-control	chronic plaque 15	6 (30)	45 ± 13.75	13	7 (53.8)	43 ± 14.75
2002	Creamer D (163)	UK		case-control	22	7 (31.8)	47 ± 12	17	7 (41.2)	42 ± 10
					severe 11					
					moderate 11					
					arthritis 10					
					non-arthritis 12					
2010	Flisiak I (164)	Poland		case-control	chronic plaque 59	16 (27.1)	49.1 ± 2.1	20		
					mild 24					
					moderate 20					
					severe 15					
2007	Fink AM (165)	Austria		case-control	arthritis 28	10 (35.7)	54 ± 13	9	2 (22.2)	56 ± 9
					active 14	4 (28.6)				
					inactive 14	6 (42.9)				
2012	Kaur S (166)	Estonia		case-control	Plaque (PV) 58	23 (39.7)	41.7 ± 12.0	58	30 (51.7)	41.4 ± 12.1
2014	Meki AR (167)	Saudi Arabia		case-control	Plaque (PV)58	22 (37.9)	30.17 ± 10.71	22	11 (50)	29.36 ± 8.83
2020	Midde HS (168)	India		cohort	54	16 (29.6)	41.28 ± 11.83	54	16 (29.6)	41.22 ± 11.77
2002	Nielsen HJ (169)	Denmark		cohort	Plaque (PV)16	9 (56.25)	24–70 years	13		
2008	Nofal A (170)	Egypt		case-control	Plaque (PV)30	11 (37)	42 ± 12.2	10	4 (40)	38.5 ± 11.6
2015	Przepiera-Bedzak H (135)	Poland		case-control	arthritis 69	39 (56.5)	52.0 ± 12.0	29	19 (65.5)	48.2 ± 13.5
2016	Przepiera-Bedzak H (136)	Poland		case-control	arthritis 76	43 (56.6)	50.8 ± 12.7	30	19 (63.3)	43.5 ± 9.4
2013	Przepiera-Bedzak H (171)	Poland		case-control	arthritis 80	43 (53.8)	50.1 ± 12.0	20	12 (60)	48.1 ± 14.0
2016	Shahidi-Dadras M (172)	Iran		case-control	severe chronic plaque psoriasis 60	27 (45)	38.35 ± 14.96	60	27 (45)	39.55 ± 15.24
2016	Shahidi-Dadras M (173)	Iran		case-control	moderate-severe chronic plaque psoriasis 58	27 (46.6)	37.5 ± 14.1	60	27 (45)	39.6 ± 15.2
2009	Takahashi H (174)	Japan		case-control	122	41 (33.6)	47.5 ± 7.6	78	24 (30.8)	38.6 ± 12.25
2017	Zheng YZ (175)	China		case-control	Plaque (PV)194	74 (38.1)	39.5 ± 12.70	175	81 (46.3)	40.2 ± 7.58
**Year**	**Author**		**Country**	**Study type**	**GD**			**HC**		
					**Sample size**	**Female (%)**	**Age (years)**	**Sample size**	**Female (%)**	**Age (years)**
2020	Cheng CW (10)		China	case-control	40	100	40.9 ± 13.5	14	100	44.1 ± 13.8
2009	Figueroa-Vega N (176)		Spain	case-control	44	32 (72.7)	45.11 ± 15.20	22	14 (63.6)	43.47 ± 8.62
					active GO 13	9 (69.2)	46.42 ± 12.58			
					inactive GO 13	10 (76.9)	48.77 ± 19.31			
					No GO 18	13 (72.2)	41.85 ± 10.76			
1998	Iitaka M (177)		Japan	case-control	49	39 (79.6)	34.7 ± 11.9	37	26 (70.3)	35.7 ± 11.2
2014	Kajdaniuk D (178)		Poland	case-control	active GO16	12 (75)	37 ± 9	22		
2016	Rancier M (179)		Tunisia	case-control	21	4 (19.0)	44.84 ± 12.10	55	29 (52.7)	46.36 ± 11.03
2014	Ye X (180)		China	case-control	64			30	20 (66.7)	32.8 ± 10.8
					GD 30	19 (63.3)	34.50 ± 13.45			
					active GO34	23 (67.6)	31.06 ± 15.15			
					inactive GO14	9 (64.3)	30.79 ± 17.80			

SLE, systemic lupus erythematosus; LN, lupus nephritis; HC, healthy control; RA, rheumatoid arthritis; HC, healthy control; SSc, systemic sclerosis; VEDOSS, very early diagnosis of systemic sclerosis; UCTD, undifferentiated connective tissue disease; HC, healthy control; BD, Behcet’s disease; HC, healthy control; KD, Kawasaki disease; HC, healthy control. AS, ankylosing spondylitis; HC, healthy control; IBD, inflammatory bowel disease; CD, Crohn’s disease; UC, ulcerative colitis; HC, healthy control; PsA, psoriasis; PV, psoriasis vulgaris; HC, healthy control; GD, Graves’ disease; GO, Graves’ ophthalmopathy; HC, healthy control.

### Meta-Analysis of the Association Between Circulating VEGF and SLE

**Figure 2:**
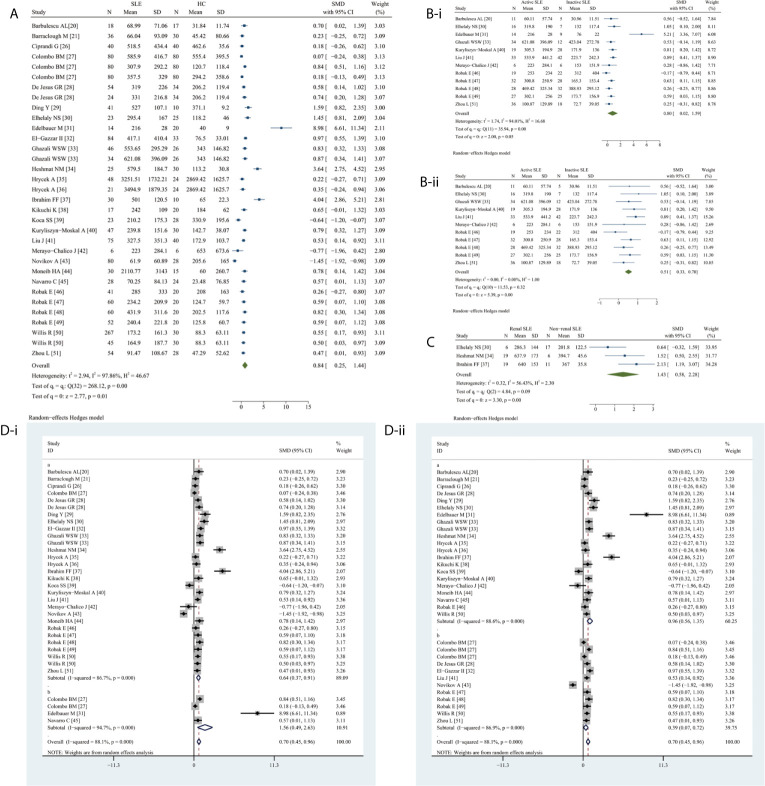
Forest plot of SLE associated with the circulating VEGF. **(A)** SLE vs. HC, forest plot; **(Bi)** Active SLE vs. Inactive SLE; **(ii)** Serum VEGF in active SLE vs. inactive SLE, forest plot; **(C)** Renal SLE vs. Non-renal SLE, forest plot; **(D)** Subgroup analysis: **(i)** Serum vs. Plasma (a for serum and b for plasma); **(ii)** Sample size n≤50 vs. n>50 (a for n≤50 and b for n>50).

Circulating VEGF levels were significantly higher in SLE than in HC (SMD 0.84, 95%CI 0.25–1.44, *P* = 0.0056) (**Figure 2A**). Additionally, circulating VEGF was higher in active SLE than in inactive SLE (SMD 0.80, 95%CI 0.02–1.59, *P* = 0.0454) (**Figure 2B-i**), serum VEGF levels remained remarkable higher in active SLE than in inactive SLE (SMD 0.51, 95% CI 0.33–0.70, *P <*0.0001) (**Figure 2B-ii**), whereas serum VEGF levels were significantly higher in SLE with renal involvement than that without renal involvement (SMD 1.43, 95% CI 0.58–2.28, *P* = 0.0010) (**Figure 2C**). Due to the observed heterogeneity, the sample types were stratified (serum versus plasma); the heterogeneity in serum VEGF levels in active and inactive SLE disappeared after removing studies using plasma (before, I^2^ = 94.04%, *P* = 0.0002; after, I^2^ = 0.00%, *P* = 0.3178).

The subgroup analysis indicated significantly higher serum (SMD 0.64, 95% CI 0.37–0.91, *P <*0.0001) and plasma (SMD 1.56, 95% CI 0.49–2.63, *P* = 0.0040) VEGF levels in SLE (**Figure 2D-i**). Significantly higher circulating VEGF levels were present in small (n ≤50) (SMD 0.96, 95% CI 0.56–1.35, *P <*0.0001) and large (n >50) (SMD 0.39, 95% CI 0.07–0.72, *P* = 0.0170) studies (**Figure 2D-ii**).

Meta-regression analysis adjusted for age and percentage of female patients demonstrated age (*P* = 0.0030) but not sex (*P* = 0.9700) had a significant effect.

### Meta-Analysis of the Association Between Circulating VEGF and RA

**Figure 3:**
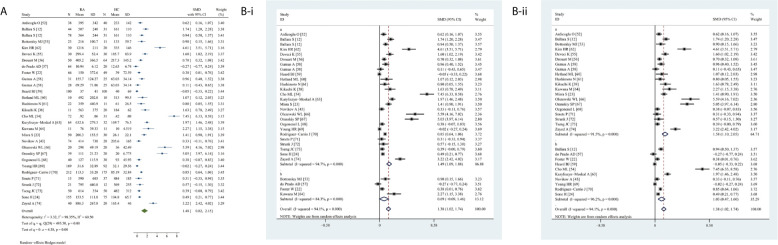
Forest plot of RA associated with the circulating VEGF. **(A)** RA vs. HC, forest plot; **(B)** Subgroup analysis: **(i)** Serum vs. Plasma (a for serum and b for plasma); **(ii)** Sample size n≤50 vs. n>50 (a for n≤50 and b for n>50).

Circulating VEGF levels were significantly higher in RA than in HC (SMD 1.48, 95% CI 0.82–2.15, *P <*0.0001) (**Figure 3A**). Overall heterogeneity was apparent.

The subgroup analysis indicated significantly higher VEGF levels in serum (SMD 1.49, 95% CI 1.09–1.88, *P <*0.0001) but not plasma (*P* = 0.0820) in RA (**Figure 3B-i**). Higher circulating VEGF levels were present in small (n ≤50) (SMD 1.58, 95% CI 1.10–2.05, *P <*0.0001) and large (n >50) (SMD 1.03, 95% CI 0.47–1.60, *P <*0.0001) studies on RA (**Figure 3B-ii**).

Meta-regression analysis adjusted for age and female sex demonstrated neither age (*P* = 0.4090) nor sex (*P* = 0.7570) had a significant effect.

### Meta-Analysis of the Association Between Circulating VEGF and SSc

**Figure 4:**
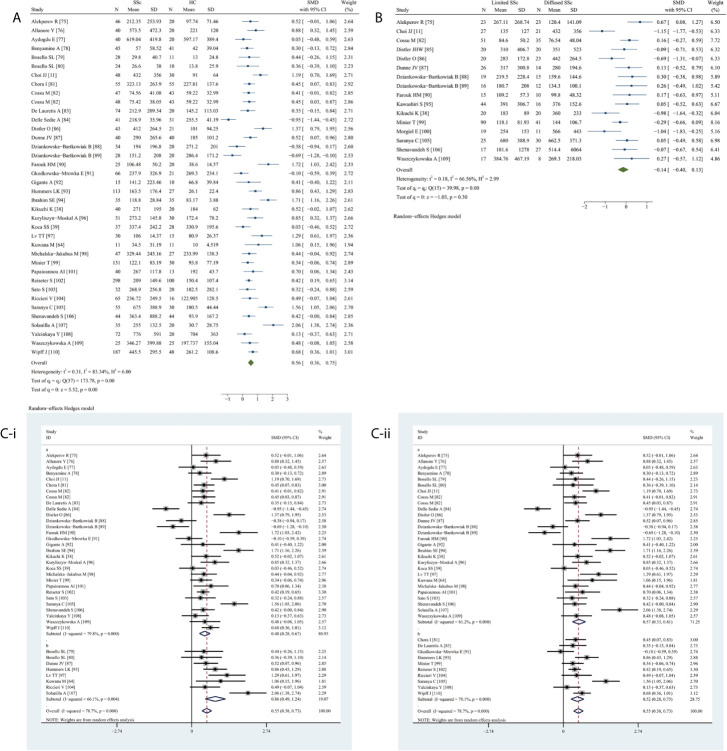
Forest plot of SSc associated with the circulating VEGF. **(A)** SSc vs. HC, forest plot; **(B)** Limited SSc vs. Diffused SSc, forest plot; **(C)** Subgroup analysis: **(i)** Serum vs. Plasma (a for serum and b for plasma); **(ii)** Sample size n≤50 vs. n>50 (a for n≤50 and b for n>50).

Circulating VEGF levels were significantly higher in SSc than in HC (SMD 0.56, 95% CI 0.36–0.75, *P <*0.0001) (**Figure 4A**). The comparison of serum VEGF levels between limited and diffused SSc did not reach statistical significance (*P* = 0.2735) (**Figure 4B**).

The subgroup analysis performed due to the obvious overall heterogeneity (I^2^ = 98.35%, *P <*0.0001) revealed significantly higher VEGF levels in serum (SMD 0.48, 95% CI 0.28–0.67, *P <*0.0001) and plasma (SMD 0.86, 95% CI 0.49–1.24, *P <*0.0001) samples of patients with SSc (**Figure 4C-i**). Elevated circulating VEGF levels were observed in small (n ≤50) (SMD 0.57, 995% CI 0.33–0.81, *P <*0.0001) and large (n >50) (SMD 0.52, 95% CI 0.28–0.75, *P <*0.0001) studies on SSc (**Figure 4C-ii**).

Meta-regression analysis adjusted for age and female sex demonstrated neither age (*P* = 0.2740) nor sex (*P* = 0.7020) had a significant effect.

### Meta-Analysis of the Association Between Circulating VEGF and BD

**Figure 5:**
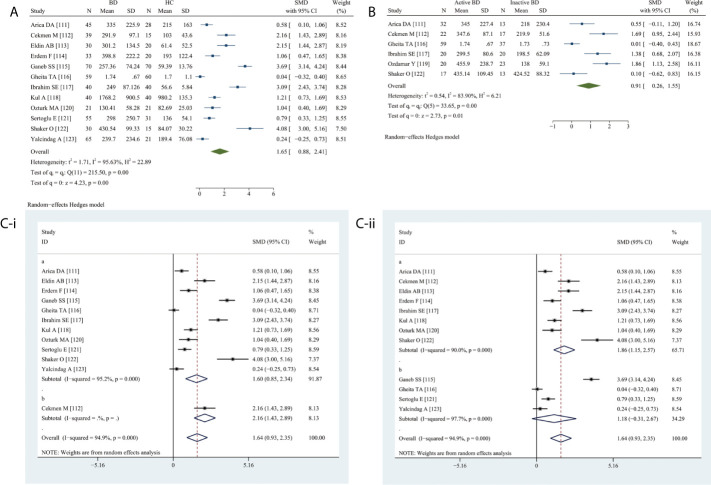
Forest plot of BD associated with the circulating VEGF. **(A)** BD vs. HC, forest plot; **(B)** Active BD vs. Inactive BD, forest plot; **(C)** Subgroup analysis: **(i)** Serum vs. Plasma (a for serum and b for plasma); **(ii)** Sample size n≤50 vs. n>50 (a for n≤50 and b for n>50).

Circulating VEGF levels were significantly higher in BD than in HC (SMD 1.65, 95% CI 0.88–2.41, *P <*0.0001) (**Figure 5A**) as well as in active BD than in inactive BD (SMD 0.91, 95% CI 0.26–1.55, *P* = 0.0064) (**Figure 5B**). Heterogeneity was present.

The subgroup analysis revealed significantly elevated serum VEGF levels (SMD 1.60, 95% CI 0.85–2.34, *P <*0.0001) (**Figure 5C-i**), specifically in small (n ≤50) (SMD 1.86, 95% CI 1.15–2.57, *P <*0.0001) and not in large (n >50) studies (*P* = 0.1200) (**Figure 5C-ii**).

Meta-regression analysis adjusted for age and female sex demonstrated neither age (*P* = 0.2700) nor sex (*P* = 0.0720) had a significant effect.

### Meta-Analysis of the Association Between Circulating VEGF and KD

Circulating VEGF levels were elevated in KD than in HC (SMD 2.41, 95% CI 0.10–4.72, *P* = 0.0406) (**Figure S1A**) and febrile controls (SMD 1.08, 95% CI 0.02–2.14, *P* = 0.0452) (**Figure S1B**). The comparison of serum VEGF levels between acute and convalescent KD revealed no statistical significance (*P* = 0.0831) (**Figure S1C**). Heterogeneity was prominent.

The subgroup analysis indicated serum VEGF levels were higher in KD than in HC (SMD 2.26, 95% CI 0.93–3.58, *P* = 0.0010) (**Figure S1D-i**). Increased circulating VEGF levels were found in small (n ≤50) (SMD 1.36, 95% CI 0.45–2.27, *P* = 0.0030) and large (n >50) studies (SMD 3.19, 95% CI 1.01–5.38, *P* = 0.0040) (**Figure S1D-ii**). Meta-regression analysis adjusted for age and female sex demonstrated female sex (*P* = 0.0100) but not age (*P* = 0.1280) had a significant effect.

### Meta-Analysis of the Association Between Circulating VEGF and AS

Circulating VEGF levels were significantly elevated in AS than in HC (SMD 0.78, 95% CI 0.23–1.33, *P* = 0.0052) (**Figure S2A**). The overall heterogeneity was apparent (I^2^ = 95.68%, *P <*0.0001).

The subgroup analysis revealed significantly higher serum VEGF levels in AS than in HC (SMD 0.60, 95% CI 0.36–0.84, *P <*0.0001) (**Figure S2B-i**). Significantly elevated circulating VEGF levels were found in small (n ≤50) (SMD 1.66, 95% CI 0.35–2.98, *P* = 0.0130) and large (n >50) studies (SMD 0.55, 95% CI 0.29–0.80, *P <*0.0001) on AS (**Figure S2B-ii**).

Meta-regression analysis adjusted for age and female sex demonstrated neither age (*P* = 0.8040) nor sex (*P* = 0.8500) had a significant effect.

### Meta-Analysis of the Association Between Circulating VEGF and IBD

Serum VEGF levels were significantly higher in IBD than in HC (SMD 0.57, 95% CI 0.43–0.71, *P <*0.0001) (**Figure S3A**). The overall heterogeneity was extremely low (I^2^ = 3.12%, *P <*0.0001). Meta-regression analysis adjusted for age or females demonstrated insignificant effect of age (*P* = 0.0760) and sex (*P* = 0.2610).

Serum VEGF levels were significantly higher in ulcerative colitis (UC) than in HC (SMD 0.69, 95% CI 0.21–1.16, *P* = 0.0048) (**Figure S3B-i**). Both the studies on active UC and those that did not specify disease activity reported significantly higher serum VEGF levels in UC (SMD 0.75, 95% CI 0.17–1.34, *P* = 0.0120 and SMD 0.56, 95% CI 0.20–0.93, *P* = 0.0030, respectively) (**Figure S3D-i**). The serum VEGF levels were not significantly different between active and inactive UC (*P* = 0.1658) (**Figure S3C-i**). Meta-regression analysis adjusted for age and female sex demonstrated insignificant effects of age (*P* = 0.8330) and sex (*P* = 0.2150).

Serum VEGF levels were significantly higher in Crohn’s disease (CD) than in HC (SMD 0.72, 95% CI 0.29–1.16, *P* = 0.0011) (**Figure S3B-ii**). Both the studies on active CD and those that did not specify disease activity reported significantly higher serum VEGF levels in CD (SMD 0.62, 95% CI 0.10–1.15, *P* = 0.0200 and SMD 0.78, 95% CI 0.33–1.22, *P* = 0.0010, respectively) (**Figure S3D-ii**). Significantly increased serum VEGF levels were present in small (n ≤50) (SMD 0.86, 95% CI 0.32–1.40, *P* = 0.002) but not in large (n >50) studies (*P* = 0.0600) (**Figure S3D-iii**). Moreover, serum VEGF levels were significantly higher in active CD than in inactive CD (SMD 0.53, 95% CI 0.09–0.96, *P* = 0.0176) (**Figure S3C-ii**). Meta-regression analysis adjusted for age and female sex demonstrated age (*P* = 0.0120) and sex (*P* = 0.0010) had significant effects.

### Meta-Analysis of the Association Between Circulating VEGF and PsA

Circulating VEGF levels were significantly higher in PsA (SMD 0.98, 95% CI 0.62–1.34, *P <*0.0001) (**Figure S4A**), in psoriatic arthritis (SMD 0.72, 95% CI 0.12–1.32, *P* = 0.0192) (**Figure S4B**), and psoriasis with skin involvement (SMD 1.26, 95% CI 0.65–1.86, *P* = 0.0001) than in HC (**Figure S4C**). Heterogeneity was observed in the analyses.

The subgroup analysis indicated significantly higher serum (SMD 1.02, 95% CI 0.50–1.55, *P <*0.0001) and plasma (SMD 0.67, 95% CI 0.37–0.97, *P <*0.0001) VEGF levels in PsA (**Figure S4D-i**). Significantly higher circulating VEGF levels were found in small (n ≤50) (SMD 0.80, 95% CI 0.49–1.11, *P <*0.0001) and large (n >50) (SMD 1.12, 95% CI 0.40–1.83, *P* = 0.0020) studies on PsA (**Figure S4D-ii**). Meta-regression analysis adjusted for age and female sex demonstrated that neither age (*P* = 0.0570) nor sex (*P* = 0.1890) had a significant effect.

### Meta-Analysis of the Association Between Circulating VEGF and GD

Circulating VEGF levels were significantly higher in GD than in HC (SMD 0.69, 95% CI 0.20–1.19, *P* = 0.0056), with considerable heterogeneity (**Figure S5A**). Circulating VEGF levels were higher in active than in inactive Graves’ ophthalmopathy (GO) (SMD 0.80, 95% CI 0.29–1.30, *P* = 0.0019), without any heterogeneity (I^2^ = 0.00%, *P* = 0.7548) (**Figure S5B**).

Serum (SMD 0.77, 95% CI 0.27–1.28, *P* = 0.0020) but not plasma (*P* = 0.3880) VEGF levels were significantly higher in GD than in HC (**Figure S5C**). Meta-regression analysis adjusted for age and female sex demonstrated the significant effect of age (*P* = 0.0070) but not sex (*P* = 0.2420).

### Correlation Analyses Between Circulation VEGF and AD Clinical Features

We explored the potential correlation of VEGF in clinical implications and hematological indicators of ADs. For SLE (**Figure S6**), the summary Fisher’s z showed a positive, moderate correlation between circulating VEGF level and disease activity (SLEDAI/SLAM, ES 0.55, 95% CI 0.29–0.81, *P <*0.0001; summary r = 0.50), erythrocyte sedimentation rate (ESR; ES 0.40, 95% CI 0.18–0.63, *P* = 0.0004; summary r = 0.38). A negative, poor correlation was found for C3 (ES −0.45, 95% CI −0.81 to −0.08, *P* = 0.0162, summary r = −0.42). There was no correlation between circulating VEGF level and platelet count (*P* = 0.1163).

In RA (**Figure S7**), there was a positive, weak correlation between circulating VEGF and disease activity (DAS-28; ES 0.33, 95% CI 0.22–0.44, *P <*0.0001, summary r = 0.32), ESR (ES 0.35, 95% CI 0.18–0.51, *P <*0.0001; summary r = 0.34) as well as C-reactive protein (CRP; ES 0.38, 95% CI 0.24–0.52, *P <*0.0001; summary r = 0.36).

In SSc (**Figure S8**), there was a positive, moderate relationship between circulating VEGF level and pulmonary artery pressure (ES 0.62, 95% CI 0.37–0.87, *P <*0.0001; summary r = 0.55) and Medical Research Council dyspnea score (ES 0.65, 95% CI 0.08–1.22, *P* = 0.0246; summary r = 0.57). There was no relationship between circulating VEGF level and modified Ronan skin score (*P* = 0.3100).

In BD (**Figure S9**), summary correlation coefficients indicated a significant, positive, and strong correlation with disease activity based on Behcet’s disease current activity form score (ES 1.22, 95% CI 0.03–2.41, *P* = 0.0446, summary r = 0.84) and moderate correlation with ESR (ES 0.47, 95% CI 0.11–0.82, *P* = 0.0108, summary r = 0.44).

In AS (**Figure S10**), circulating VEGF level was poorly correlated with disease activity (BASDAI/BASMI; ES 0.35, 95% CI 0.09–0.60, *P* = 0.0080; summary r = 0.34), ESR (ES 0.26, 95% CI 0.17–0.36, *P <*0.0001; summary r = 0.25), and CRP (ES 0.24, 95% CI 0.14–0.35, *P <*0.0001; summary r = 0.24).

In IBD (**Figure S11**), circulating VEGF level exhibited a positive, poor correlation with Crohn’s disease activity index (CDAI; ES 0.34, 95% CI 0.10–0.57, *P* = 0.0053, summary r = 0.33), medium correlation with UC activity index (UDAI; ES 0.57, 95% CI 0.29–0.86, *P* = 0.0001; summary r = 0.52), strong correlation with ESR (ES 0.87, 95% CI 0.63–1.12, *P <*0.0001; summary r = 0.70), and weak correlation with platelet count (ES 0.32, 95% CI 0.16–0.49, *P* = 0.0001; summary r 0.31).

In PsA (**Figure S12**), circulating VEGF level was positively correlated with psoriasis area and severity index score (ES 1.12, 95% CI 0.64–1.60, *P <*0.0001; summary r = 0.81) and had a positive, moderate correlation with disease duration (ES 0.51, 95% CI 0.32–0.69, *P <*0.0001; summary r = 0.47).

### Sensitivity Analysis and Publication Bias

The sensitivity analysis revealed the stability of pooled results (data not shown). For SLE, RA, SSc, KD, and AS, the contour-enhanced funnel plots revealed no publication bias (**Figure S13**), the meta-trim practice demonstrated that all imputed studies fell into the significant region. In contrast, Egger’s test suggested publication bias for SLE, RA, and KD (*P <*0.0001 for all) as well as for AS (*P* = 0.0001). However, there was consistency in publication bias for SSc by Egger’s test (*P* = 0.1413). This remind us to be cautious with using Egger’s test to determine publication bias in small number of studies (<20). There was no publication bias with PsA and GD (*P* = 0.4874 and *P* = 0.5419, respectively), in contrast to that observed with BD (*P* = 0.0006). The imputed studies on IBD fell into the non-significant region, and Egger’s test also represented evidence of it (*P* = 0.0017) in UC; the existence of publication bias was proven by Egger’s test (*P* = 0.0113) in CD.

## Discussion

In the current meta-analysis, we found a close relationship between circulating VEGF level and ADs. First, our analyses revealed significantly increased circulating VEGF levels in SLE, RA, SSc, BD, KD, AS, IBD, PsA, and GD. Additionally, we showed that serum VEGF could distinguish active from inactive SLE and renal from non-renal SLE; it could also discriminate between active and inactive CD. Likewise, circulating VEGF had a strong ability to differentiate active from inactive BD and GO. Serum VEGF exhibited its dipartite boundedness in limited/diffused cutaneous SSc, active/inactive UC, and acute/convalescent KD. Furthermore, we demonstrated the correlation of circulating VEGF levels with metrics of disease activity and severity (SLEDAI/SLAM, DAS-28, MRC dyspnea score, modified Ronan skin score, BD current activity form score, BASDAI/BASMI, CDAI, UDAI, psoriasis area and severity index) as well as with hematological parameters (ESR, CRP, platelet count, pulmonary artery pressure). Overall, these results indicate that circulating VEGF reflects pathogenesis and should be considered as a potent hematological marker for diagnosis and disease progression in ADs.

Structural and functional abnormities in neovasculature may lead to damage in chronic inflammatory diseases. Consecutive angiogenesis and immune-mediated vascular endothelial cell injury and dysfunction as well as persistent inflammation play important pathological roles in SLE (20), whereas expansion and invasion of synovial vessels facilitate inflammation and erosive joint destruction in RA (12). Early generalized microvascular endothelial damage leading to immune activation and defective angiogenesis are significant events in cumulative systemic fibrosis and microangiopathy in SSc (76). Additionally, BD is characterized by systemic vasculitis, inflammatory infiltrates, subsequent vascular lesions, and neovascularization (113, 115), whereas subendothelial edema and fenestrated endothelium constitute acute systemic vasculitis observed in KD (181). Structural changes in vascular endothelium due to inflammation and hypoxia stimulate angiogenesis to permeate vascular and mediate tissue repair in IBD (6). Finally, early psoriatic skin plaque formation is triggered by inappropriate expansion and vascular alterations, pronounced permeability, and endothelial cell proliferation (162). Therefore, angiogenesis and angiopathy are considered as major pathogenic events predisposing to ADs.

VEGF, an increasingly recognized proangiogenic inducer of endothelial proliferation and microvascular hyperpermeability, may reverse the tide of inducers against inhibitors and promote angiogenesis (182). Despite the unclear role of angiogenesis in AS and GD, higher-than-normal VEGF levels support its role in bone and enchondral ossification in AS (183) and increased microvessel density in GD (184). Over the past decades, numerous studies have reported increased VEGF levels in ADs, beyond its well-known role in tumorigenesis. In the present study, our meta-analysis reveals differences in circulating VEGF levels between patients with ADs and HC subjects, providing further evidence for its utility in determining disease activity and severity in ADs.

In the present meta-analysis, there were variations in circulating VEGF levels due to differences in sample collection methods and demographic characteristics across the studies, requiring adjustment for the interpretation of the final laboratory results. Serum VEGF levels are 7–10 times higher than plasma VEGF levels in RA (60). Serum VEGF is a combination of efflux from platelets, neutrophils during coagulation, and circulating VEGF, which rarely occurs *in vivo*; in contrast, plasma VEGF directly reflects circulating VEGF in the absence of coagulation *in vivo*. In support of this difference, the present meta-analysis also revealed that the removal of plasma samples from the analysis led to the disappearance of heterogeneity in serum VEGF levels in active and inactive SLE. Plasma samples with citrate anticoagulants had the lowest VEGF levels, reflecting that that reservation of platelets VEGF releasing is effective and that different anticoagulation procedures should be considered in evaluating variations in VEGF levels across studies. Higher plasma VEGF levels in female patients compared with male patients, increasing VEGF levels with age in adults, and decreasing VEGF levels with age in children illustrate the contributory roles of sex and age to discrepancy (185). The cohort size in specific studies might also impact the mean and standard deviation. Therefore, we addressed these variables in subgroup and meta-regression analyses. The subgroup analyses explored the source of heterogeneity in serum VEGF levels for only studies on active and inactive SLE (before, I^2^ = 94.04%, *P* = 0.0002; after, I^2^ = 0.00%, *P* = 0.3178). We also observed apparent associations of circulating VEGF levels with age and female sex in SLE and CD, with sex in KD, and with age in GD.

There are several limitations in the present meta-analysis. First, although subgroup and meta-regression analyses were performed to explore heterogeneity, much of it remains to be explained and reported. Second, the funnel plots indicated publication bias in studies on BD and IBD, including UC as well as CD, which might have led to the overestimation of pooled SMDs. Third, data could not be fully retrieved, which might have resulted in missing values in meta-regression and the omission of covariates in tests assessing heterogeneity. Availability of complete data on patient inclusion and exclusion criteria, ethnicity, AD treatment details, and exact timing and method of VEGF measurement would greatly reduce the bias in our analyses. Although the existing heterogeneity could be partially explained by age, sex, sample type, and sample size of the individual studies, an exact conclusion could not be drawn due to the lacking explanation for the remaining heterogeneity. Further studies using more comprehensive data should be performed to elucidate the association of circulating VEGF levels with ADs.

In conclusion, our meta-analysis unveiled a close association between circulating VEGF levels and ADs including disease activity and severity as well as clinical hematological manifestations. Serum VEGF is a reliable marker that can distinguish active from inactive in SLE and GO and can potentially differentiate IBD from HC. Early and regular measurement of circulating VEGF levels may be considered as a noninvasive method to monitor vascular involvement and activity in ADs. Future studies should focus on the prognostic and diagnostic utility of circulating VEGF, its role in pathogenesis, and the utility of VEGF-targeted therapeutic strategies in ADs.

## Data Availability Statement

The original contributions presented in the study are included in the article/**Supplementary Material**. Further inquiries can be directed to the corresponding author.

## Author Contributions

YL conceived and designed the research. HZ and HL extracted data and conducted quality assessment. CL, LC, SY, HL, and HZ analyzed the data. HZ wrote the paper. All authors are accountable for all aspects of the study, and attest to the accuracy and integrity of the results. All authors contributed to the article and approved the submitted version.

## Funding

This research was supported by grants from the National Natural Science Foundation of China Grants (81871302) and Beijing Key Clinical Specialty for Laboratory Medicine - Excellent Project (No. ZK201000).

## Conflict of Interest

The authors declare that the research was conducted in the absence of any commercial or financial relationships that could be construed as a potential conflict of interest.
