# Supplementary material for: Association of Circulating Vascular Endothelial Growth Factor Levels With Autoimmune Diseases: A Systematic Review and Meta-Analysis
Source: Front Immunol. 2021 May 27;12:674343. doi: 10.3389/fimmu.2021.674343 (PMC8191579; doi:10.3389/fimmu.2021.674343)
Supplement: Supplementary file 1 [file DataSheet_1.docx]

**Supplementary Materials**

**Supplementary Methodology**

**Search strategy**

#1 Lupus Erythematosus, Systemic [MESH]

#2 “lupus*” OR SLE [Tittle/Abstract]

#3 #1 OR #2

#4 Arthritis, Rheumatoid [MESH]

#5 RA OR ((rheumatoid OR reumatoid OR revmatoid OR rheumatic OR reumatic OR revmatic OR rheumat* OR reumat* OR revmarthrit*) AND (arthrit* OR artrit* OR diseas* OR condition* OR nodule*)) [Tittle/Abstract]

#6 #4 OR #5

#7 Scleroderma, Systemic [MESH]

#8“Scleroderma” OR “Systemic Sclerosis” OR SSc [Tittle/Abstract]

#9 #7 OR #8

#10 Behcet Syndrome [MESH]

#11 BD OR (Behcet* AND (syndrome* OR disease*)) OR ("triple‐complex syndrome*" OR "triple‐complex disease*") [Tittle/Abstract]

#12 #10 OR #11

#13 Mucocutaneous Lymph Node Syndrome [MESH]

#14 kawasaki* OR (mucocutan* AND syndrome*) [Tittle/Abstract]

#15 #13 OR #14

#16 Spondylitis, Ankylosing [MESH]

#17 AS OR ankylos* OR spondyl* OR bechtere* disease* OR marie‐struempell disease* OR rheumatoid spondylitis OR Vertebral Ankylosis [Tittle/Abstract]

#18 #16 OR #17

#19 Inflammatory Bowel Diseases [MESH]

#20 “Inflammatory bowel disease” OR “IBD” OR crohn* OR ileitis OR enteritis OR proctitis [Tittle/Abstract]

#21 #19 OR #20

#22 Psoriasis [MESH]

#23 psoria* OR (palmoplantar* AND pustulosis) OR “pustulosis palmaris et plantaris” OR “pustulosis of palms and soles”

#24 #22 OR #23

#25 Graves Disease [MESH]

#26 (grave* AND (diseas* OR thyrotoxicos* OR hyperthyr* OR orbitopath* OR ophthalmopath*)) OR (basedow* AND (diseas* OR syndrom*)) OR (exophthalmic AND (goiter* OR goitre* OR hyperthyroidism*)) [Tittle/Abstract]

#27 #25 OR #26

#28 Vascular Endothelial Growth Factors [MESH]

#29 “Vascular Endothelial Growth Factor*” OR VEGF OR Vasculotropin OR “Vascular Permeability Factor*” [Tittle/Abstract]

#30 #28 OR #29

#31 #3 OR #6 OR #9 OR #12 OR #15 OR #18 OR #21 OR #24 OR #27

#32 #30 AND #31

**Transformation strategy**

Spearman correlation coefficients were transformed into Pearson’s r, from which Fisher’s z was converted for an approximately normal distribution. Ultimately, the summary fisher’s z undergone a conversion into summary r.

**Formula**

**First**, **Spearman** correlation coefficients **r_s_** were transformed into **Pearson’s** **r** according to:

**r = 2sin(r_s_×**$\frac{\boldsymbol{\pi}}{\mathbf{6}}$**)**

**Second**, **Pearson’s r** was converted into **Fisher’s z** according to:

**Fisher’s z = 0.5×**$\ln\frac{\mathbf{1+}\boldsymbol{r}}{\mathbf{1-}\boldsymbol{r}}$

**Third, Fisher’s z** and **standard error (SE)** were input into STATA 16.0 and then generalized **summary fisher’s z.**

**V_s_ =**$\frac{\boldsymbol{1}}{\boldsymbol{n-3}}$

**S_E_ =**$\sqrt{\boldsymbol{v}_{\boldsymbol{z}}}$

**Ultimately**, **summary fisher’s z** was transformed into **summary r** according to:

**summary r =** $\frac{\boldsymbol{ⅇ}^{\boldsymbol{2}\boldsymbol{z}}\boldsymbol{-1}}{\boldsymbol{ⅇ}^{\boldsymbol{2}\boldsymbol{z}}\boldsymbol{+1}}$ **(i.e. z is summary fisher’s z)**

**Fabs of summary r** ranging from 0.8-1.0 represent extremely relevance between circulating vascular endothelial growth factor levels and disease activity, as well as hematological parameters in AD patients, 0.6-0.8 for highly relevant, 0.4-0.6 for moderately relevant and 0.2-0.4 for poor correlation.

| **Table S1 Circulating VEGF levels of the studies included in the meta-analysis** | | | | | | | | |
| --- | --- | --- | --- | --- | --- | --- | --- | --- |
|  |  | SLE |  |  | Healthy control | | |  |
| Year | Author | N1 | Mean1 | SD1 | N2 | Mean2 | SD2 | Sample type |
| 2015 | Barbulescu AL[20] | 18 | 68.99 | 71.06 | 17 | 31.84 | 11.74 | serum |
| 2019 | Barraclough M [21] | 36 | 66.04 | 93.09 | 30 | 45.42 | 80.66 | —— |
| 2008 | Ciprandi G [26] | 40 | 518.5 | 434.4 | 40 | 462.6 | 35.6 | serum |
| 2009 | Colombo BM [27] | 80 | 585.9 | 416.7 | 80 | 555.4 | 395.5 | serum |
| 2009 | Colombo BM [27] | 80 | 307.9 | 292.2 | 80 | 120.7 | 118.4 | CTAD plasma |
| 2009 | Colombo BM [27] | 80 | 357.5 | 329.0 | 80 | 294.2 | 358.6 | EDTA plasma |
| 2014 | De Jesus GR [28] | 54 | 319.0 | 226.0 | 34 | 206.2 | 119.4 | serum |
| 2014 | De Jesus GR [28] | 24 | 331.0 | 216.8 | 34 | 206.2 | 119.4 | serum |
| 2015 | Ding Y [29] | 41 | 527.0 | 107.1 | 10 | 371.1 | 9.2 | serum |
| 2009 | Elhelaly NS [30] | 23 | 295.4 | 167 | 25 | 118.2 | 46 | serum |
| 2012 | Edelbauer M [31] | 14 | 216 | 28 | 20 | 40 | 9 | plasma |
| 2018 | El-Gazzar II [32] | 84 | 417.1 | 410.4 | 33 | 76.5 | 33.01 | serum |
| 2017 | Ghazali WSW [33] | 46 | 553.65 | 295.29 | 26 | 343.00 | 146.82 | serum |
| 2017 | Ghazali WSW [33] | 34 | 621.08 | 396.09 | 26 | 343.00 | 146.82 | serum |
| 2007 | Heshmat NM [34] | 25 | 579.5 | 184.7 | 30 | 113.2 | 30.8 | serum |
| 2009 | Hrycek A [35] | 48 | 3251.51 | 1732.21 | 24 | 2869.42 | 1625.70 | serum |
| 2009 | Hrycek A [36] | 21 | 3494.90 | 1879.35 | 24 | 2869.42 | 1625.70 | serum |
| 2008 | Ibrahim FF [37] | 30 | 501 | 120.5 | 10 | 65 | 22.3 | serum |
| 1998 | Kikuchi K [38] | 17 | 242 | 109 | 20 | 184 | 62 | serum |
| 2013 | Koca SS [39] | 23 | 210.2 | 175.3 | 28 | 330.9 | 195.6 | serum |
| 2007 | Kuryliszyn-Moskal A [40] | 47 | 239.8 | 151.6 | 30 | 142.7 | 38.07 | serum |
| 2014 | Liu J [41] | 75 | 327.5 | 351.3 | 40 | 172.9 | 103.7 | serum |
| 2018 | Merayo-Chalico J [42] | 6 | 223 | 284.1 | 6 | 653 | 673.6 | serum |
| 2016 | Novikov A [43] | 80 | 61.9 | 60.89 | 28 | 205.6 | 165 | serum |
| 2012 | Moneib HA [44] | 30 | 2110.77 | 3143 | 15 | 60.00 | 260.7 | serum |
| 2002 | Navarro C [45] | 28 | 70.25 | 84.13 | 24 | 23.48 | 76.85 | plasma |
| 2005 | Robak E [46] | 41 | 285 | 333 | 20 | 208 | 163 | serum |
| 2003 | Robak E [47] | 60 | 234.2 | 209.9 | 20 | 124.7 | 59.7 | serum |
| 2013 | Robak E [48] | 60 | 431.9 | 311.6 | 20 | 202.5 | 117.6 | serum |
| 2002 | Robak E [49] | 52 | 240.4 | 221.8 | 20 | 125.8 | 60.7 | serum |
| 2017 | Willis R [50] | 267 | 173.2 | 161.3 | 30 | 88.3 | 63.11 | serum |
| 2017 | Willis R [50] | 45 | 164.9 | 187.7 | 30 | 88.3 | 63.11 | serum |
| 2014 | Zhou L [51] | 54 | 91.47 | 108.67 | 28 | 47.29 | 52.62 | serum |
|  |  | Active SLE | | | Inactive SLE | | |  |
| Year | Author | N1 | Mean1 | SD1 | N2 | Mean2 | SD2 | Sample type |
| 2015 | Barbulescu AL [20] | 11 | 60.11 | 57.74 | 5 | 30.96 | 11.51 | serum |
| 2009 | Elhelaly NS [30] | 16 | 319.8 | 190.0 | 7 | 132 | 117.4 | serum |
| 2012 | Edelbauer M [31] | 14 | 216 | 28 | 9 | 76 | 22 | plasma |
| 2017 | Ghazali WSW [33] | 34 | 621.08 | 396.09 | 12 | 423.04 | 272.78 | serum |
| 2007 | Kuryliszyn-Moskal A [40] | 19 | 305.3 | 194.9 | 28 | 171.9 | 136.0 | serum |
| 2014 | Liu J [41] | 33 | 533.9 | 441.2 | 42 | 223.7 | 242.3 | serum |
| 2018 | Merayo-Chalico J [42] | 6 | 223 | 284.1 | 6 | 153 | 151.9 | serum |
| 2005 | Robak E [46] | 19 | 253 | 234 | 22 | 312 | 404 | serum |
| 2003 | Robak E [47] | 32 | 300.8 | 250.9 | 28 | 165.3 | 153.4 | serum |
| 2013 | Robak E [48] | 28 | 469.42 | 325.34 | 32 | 388.93 | 295.12 | serum |
| 2002 | Robak E [49] | 27 | 302.1 | 256.0 | 25 | 173.7 | 156.9 | serum |
| 2014 | Zhou L [51] | 36 | 100.87 | 129.89 | 18 | 72.70 | 39.05 | serum |

| **Table S1 Circulating VEGF levels of the studies included in the meta-analysis** | | | | | | | | |
| --- | --- | --- | --- | --- | --- | --- | --- | --- |
|  |  | RA |  |  | Healthy control | | |  |
| Year | Author | N1 | Mean1 | SD1 | N2 | Mean2 | SD2 | Sample type |
| 2004 | Ardicoglu O [52] | 38 | 395 | 342 | 40 | 233 | 142 | serum |
| 2001 | Ballara S [12] | 44 | 507 | 240 | 31 | 161 | 110 | serum |
| 2001 | Ballara S [12] | 78 | 364 | 244 | 31 | 161 | 110 | serum |
| 2000 | Bottomley MJ [53] | 23 | 216 | 100.7 | 11 | 133 | 59.7 | EDTA plasma |
| 2005 | Kim HR [62] | 30 | 1216 | 211 | 20 | 335 | 146 | serum |
| 2016 | Deveci K [55] | 30 | 299.4 | 52.4 | 30 | 185.7 | 83.9 | serum |
| 2002 | Drouart M [56] | 50 | 405.2 | 366.5 | 64 | 217.3 | 145.2 | serum |
| 2016 | do Prado AD [57] | 64 | 10.94 | 6.12 | 20 | 12.63 | 6.74 | plasma |
| 2008 | Foster W [22] | 66 | 150 | 372.6 | 49 | 39 | 72.59 | CTAD plasma |
| 2018 | Gumus A [58] | 31 | 155.70 | 124.27 | 25 | 63.03 | 34.14 | serum |
| 2018 | Gumus A [58] | 28 | 69.29 | 71.06 | 25 | 63.03 | 34.14 | serum |
| 2014 | Heard BJ [59] | 100 | 37 | 41 | 100 | 40 | 69 | serum |
| 2008 | Hetland ML [60] | 10 | 492 | 202.8 | 10 | 313 | 99.5 | serum |
| 2003 | Hashimoto N [61] | 22 | 359 | 440.9 | 11 | 61 | 26.5 | serum |
| 1998 | Kikuchi K [38] | 11 | 563 | 375 | 20 | 184 | 62 | serum |
| 2007 | Cho ML [54] | 72 | 0.92 | 0.06 | 31 | 0.42 | 0.08 | serum |
| 2006 | Kuryliszyn-Moskal A [63] | 64 | 632.6 | 279.3 | 32 | 169.7 | 76.3 | serum |
| 2004 | Kuwana M [64] | 11 | 76.0 | 39.33 | 11 | 10.0 | 4.519 | heparinised platelet-poor plasma |
| 2018 | Misra S [23] | 50 | 200.3 | 153.5 | 30 | 26.10 | 22.10 | serum |
| 2016 | Novikov A [43] | 74 | 414.0 | 738 | 28 | 205.6 | 165 | serum |
| 1999 | Olszewski WL [66] | 20 | 298 | 49.19 | 20 | 36.0 | 42.49 | serum |
| 2012 | Oranskiy SP [67] | 39 | 111.0 | 21.23 | 20 | 20 | 6.775 | serum |
| 2010 | Ozgonenel L [68] | 40 | 127 | 115.9 | 38 | 93 | 45.93 | serum |
| 2009 | Young HR [69] | 169 | 31.6 | 32.89 | 92 | 32.1 | 29.56 | serum |
| 2016 | Rodriguez-Carrio J [70] | 212 | 113.30 | 33.28 | 175 | 85.19 | 32.84 | serum |
| 2016 | Smets P [71] | 13 | 590 | 603 | 37 | 484 | 183 | serum |
| 2004 | Strunk J [72] | 21 | 795 | 440.8 | 12 | 569 | 255 | serum |
| 2010 | Tseng JC [73] | 50 | 614 | 354 | 50 | 482 | 312 | serum |
| 2001 | Sone H [24] | 155 | 153.5 | 111.8 | 75 | 104.8 | 65.7 | serum |
| 2007 | Zayed A [74] | 40 | 886.3 | 267.8 | 20 | 165.4 | 46 | serum |

| **Table S1 Circulating VEGF levels of the studies included in the meta-analysis** | | | | | | | | | | | |
| --- | --- | --- | --- | --- | --- | --- | --- | --- | --- | --- | --- |
|  |  | SSc |  |  | Healthy control | | | |  | | |
| Year | Author | N1 | Mean1 | SD1 | N2 | Mean2 | SD2 | | Sample type | | |
| 2018 | Alekperov R [75] | 46 | 212.35 | 253.93 | 20 | 97.74 | 71.46 | serum | | |  |
| 2004 | Allanore Y [76] | 40 | 573.5 | 472.3 | 20 | 221 | 120 | serum | | |  |
| 2013 | Aydogdu E [77] | 40 | 619.04 | 419.8 | 20 | 597.17 | 389.4 | serum | | |  |
| 2017 | Benyamine A [78] | 45 | 57 | 58.52 | 41 | 42 | 39.04 | serum | | |  |
| 2014 | Bosello SL [79] | 28 | 29.8 | 40.7 | 11 | 13.0 | 24.8 | plasma | | |  |
| 2014 | Bosello SL [80] | 24 | 26.6 | 38 | 10 | 13.8 | 25.9 | plasma | | |  |
| 2002 | Choi JJ [11] | 48 | 432 | 356 | 30 | 91 | 64 | serum | | |  |
| 2017 | Chora I [81] | 55 | 323.13 | 263.9 | 55 | 227.81 | 137.6 | serum | | |  |
| 2016 | Cossu M [82] | 47 | 74.56 | 41.08 | 43 | 59.22 | 32.99 | serum | | |  |
| 2016 | Cossu M [82] | 48 | 75.42 | 38.05 | 43 | 59.22 | 32.99 | serum | | |  |
| 2013 | De Lauretis A [83] | 74 | 212.9 | 209.34 | 20 | 145.2 | 113.03 | serum | | |  |
| 2017 | Delle Sedie A [84] | 41 | 218.9 | 35.96 | 31 | 255.5 | 41.19 | serum | | |  |
| 2002 | Distler O [86] | 43 | 412 | 264.5 | 21 | 101 | 94.25 | serum | | |  |
| 2012 | Dunne JV [87] | 40 | 290 | 265.6 | 40 | 185 | 101.2 | plasma | | |  |
| 2005 | Dziankowska-Bartkowiak B [88] | 34 | 194.0 | 196.8 | 20 | 271.2 | 201.0 | serum | | |  |
| 2006 | Dziankowska-Bartkowiak B [89] | 28 | 151.2 | 208 | 20 | 286.4 | 171.2 | serum | | |  |
| 2013 | Farouk HM [90] | 25 | 106.48 | 50.2 | 20 | 38.6 | 14.57 | serum | | |  |
| 2014 | Gkodkowska-Mrowka E [91] | 66 | 237.9 | 326.9 | 21 | 269.3 | 234.1 | serum | | |  |
| 2018 | Gigante A [92] | 15 | 141.2 | 223.46 | 10 | 66.8 | 39.84 | serum | | |  |
| 2008 | Hummers LK [93] | 113 | 163.5 | 176.4 | 27 | 26.1 | 22.4 | plasma | | |  |
| 2017 | Ibrahim SE [94] | 35 | 118.80 | 28.84 | 35 | 83.17 | 3.88 | serum | | |  |
| 1998 | Kikuchi K [38] | 40 | 271 | 195 | 20 | 184 | 62 | serum | | |  |
| 2004 | Kuryliszyn-Moskal A [96] | 31 | 273.2 | 145.8 | 30 | 172.4 | 78.2 | serum | | |  |
| 2013 | Koca SS [39] | 37 | 337.4 | 242.2 | 28 | 330.9 | 195.6 | serum | | |  |
| 2020 | Lv TT [97] | 30 | 106.0 | 14.37 | 15 | 80.9 | 26.37 | plasma | | |  |
| 2004 | Kuwana M [64] | 11 | 34.5 | 31.19 | 11 | 10.0 | 4.519 | heparinised platelet-poor plasma | | |  |
| 2019 | Michalska-Jakubus M [98] | 47 | 329.44 | 245.16 | 27 | 233.99 | 138.30 | serum | | |  |
| 2010 | Minier T [99] | 131 | 122.1 | 83.19 | 30 | 93.8 | 77.19 | serum | | |  |
| 2009 | Papaioannou AI [101] | 40 | 267 | 117.8 | 13 | 192 | 43.7 | serum | | |  |
| 2015 | Reiseter S [102] | 298 | 209.0 | 149.6 | 100 | 150.4 | 107.4 | serum | | |  |
| 2001 | Sato S [103] | 32 | 268.9 | 256.8 | 20 | 182.5 | 282.1 | serum | | |  |
| 2010 | Riccieri V [104] | 65 | 236.72 | 249.5 | 16 | 122.905 | 128.5 | plasma | | |  |
| 2017 | Saranya C [105] | 55 | 675 | 388.9 | 30 | 180.5 | 44.44 | serum | | |  |
| 2016 | Shenavandeh S [106] | 44 | 363.4 | 888.2 | 44 | 93.9 | 167.2 | serum | | |  |
| 2009 | Solanilla A [107] | 35 | 255 | 132.5 | 20 | 30.7 | 28.75 | EDTA plasma | | |  |
| 2016 | Yalcinkaya Y [108] | 72 | 776 | 591 | 20 | 704 | 363 | serum | | |  |
| 2020 | Waszczykowska A [109] | 25 | 346.27 | 399.88 | 25 | 197.737 | 155.04 | serum | | |  |
| 2008 | Wipff J [110] | 187 | 445.5 | 295.5 | 48 | 261.2 | 108.6 | serum | | |  |
|  |  | Limited SSc | | | Diffused SSc | | | |  |  |  |
| Year | Author | N1 | Mean1 | SD1 | N2 | Mean2 | SD2 | | Sample type |  |  |
| 2018 | Alekperov R [75] | 23 | 267.11 | 268.74 | 23 | 120.40 | 141.09 | | serum |  |  |
| 2002 | Choi JJ [11] | 27 | 135 | 127 | 21 | 432 | 356 | | serum |  |  |
| 2016 | Cossu M [82] | 51 | 84.6 | 50.2 | 35 | 76.54 | 48.04 | | serum |  |  |
| 2011 | Distler JHW [85] | 20 | 310 | 406.7 | 20 | 351 | 523 | | serum |  |  |
| 2002 | Distler O [86] | 20 | 283 | 172.8 | 23 | 442 | 264.5 | | serum |  |  |
| 2012 | Dunne JV [87] | 26 | 317 | 300.8 | 14 | 280 | 194.6 | | plasma |  |  |
| 2005 | Dziankowska-Bartkowiak B [88] | 19 | 219.5 | 228.4 | 15 | 159.6 | 144.6 | | serum |  |  |
| 2006 | Dziankowska-Bartkowiak B [89] | 16 | 180.7 | 208 | 12 | 134.3 | 100.1 | | serum |  |  |
| 2013 | Farouk HM [90] | 15 | 109.2 | 57.30 | 10 | 99.80 | 48.32 | | serum |  |  |
| 2018 | Kawashiri S [95] | 44 | 391 | 306.7 | 16 | 376 | 152.6 | | serum |  |  |
| 1998 | Kikuchi K [38] | 20 | 183 | 89 | 20 | 360 | 233 | | serum |  |  |
| 2010 | Minier T [99] | 90 | 118.1 | 81.93 | 41 | 144.0 | 106.7 | | serum |  |  |
| 2012 | Morgiel E [100] | 19 | 254 | 153 | 11 | 566 | 443 | | serum |  |  |
| 2017 | Saranya C [105] | 25 | 680 | 388.9 | 30 | 662.5 | 371.3 | | serum |  |  |
| 2016 | Shenavandeh S [106] | 17 | 181.6 | 1278 | 27 | 514.4 | 6064 | | serum |  |  |
| 2020 | Waszczykowska A [109] | 17 | 384.76 | 467.19 | 8 | 269.30 | 218.03 | | serum |  |  |

| **Table S1 Circulating VEGF levels of the studies included in the meta-analysis** | | | | | | | | |
| --- | --- | --- | --- | --- | --- | --- | --- | --- |
|  |  | BD | | | HC | | |  |
| Year | Author | N1 | Mean1 | SD1 | N2 | Mean2 | SD2 | Sample type |
| 2018 | Arica DA [111] | 45 | 335 | 225.9 | 28 | 215 | 163 | serum |
| 2003 | Cekmen M [112] | 39 | 291.9 | 97.1 | 15 | 103.0 | 43.6 | plasma |
| 2013 | Eldin AB [113] | 30 | 301.2 | 134.5 | 20 | 61.4 | 52.5 | serum |
| 2003 | Erdem F [114] | 33 | 398.8 | 222.2 | 20 | 193.0 | 122.4 | serum |
| 2012 | Ganeb SS [115] | 70 | 257.36 | 74.24 | 70 | 59.39 | 13.76 | serum |
| 2019 | Gheita TA [116] | 59 | 1.74 | 0.67 | 60 | 1.7 | 1.1 | serum |
| 2011 | Ibrahim SE [117] | 40 | 249 | 87.126 | 40 | 56.6 | 5.84 | serum |
| 2017 | Kul A [118] | 40 | 1768.2 | 900.5 | 40 | 980.2 | 135.3 | serum |
| 2007 | Ozturk MA [120] | 21 | 130.41 | 58.28 | 21 | 82.69 | 25.03 | serum |
| 2018 | Sertoglu E [121] | 55 | 298 | 250.7 | 31 | 136 | 54.1 | serum |
| 2006 | Shaker O [122] | 30 | 430.54 | 99.33 | 15 | 84.07 | 30.22 | serum |
| 2013 | Yalcindag A [123] | 65 | 239.7 | 234.6 | 21 | 189.4 | 76.08 | serum |
|  |  | Active BD | |  | Inactive BD | | |  |
| Year | Author | N1 | Mean1 | SD1 | N2 | Mean2 | SD2 | Sample type |
| 2018 | Arica DA [111] | 32 | 345 | 227.4 | 13 | 218 | 230.4 | serum |
| 2003 | Cekmen M [112] | 22 | 347.6 | 87.1 | 17 | 219.9 | 51.6 | plasma |
| 2019 | Gheita TA [116] | 59 | 1.74 | 0.67 | 37 | 1.73 | 0.73 | serum |
| 2011 | Ibrahim SE [117] | 20 | 299.5 | 80.6 | 20 | 198.5 | 62.09 | serum |
| 2009 | Ozdamar Y [119] | 20 | 455.9 | 238.7 | 23 | 138.0 | 59.1 | serum |
| 2006 | Shaker O [122] | 17 | 435.14 | 109.45 | 13 | 424.52 | 88.32 | serum |

| **Table S1 Circulating VEGF levels of the studies included in the meta-analysis** | | | | | | | | |
| --- | --- | --- | --- | --- | --- | --- | --- | --- |
|  |  | KD | | | HC | | |  |
| Year | Author | N1 | Mean1 | SD1 | N2 | Mean2 | SD2 | Sample type |
| 2011 | Breunis WB [124] | 101 | 811 | 532.6 | 18 | 411 | 271.5 | serum |
| 2001 | Hamamichi Y [125] | 49 | 281 | 126 | 38 | 74 | 80.1 | serum |
| 1998 | Maeno N [126] | 20 | 321.9 | 140.3 | 19 | 117.4 | 37.83 | serum |
| 1998 | Maeno N [126] | 13 | 320.1 | 200.2 | 19 | 117.4 | 37.83 | serum |
| 1999 | Ohno T [18] | 66 | 59.87 | 500.9 | 18 | 7.75 | 12.45 | serum |
| 1999 | Ohno T [18] | 31 | 62.50 | 500.9 | 18 | 7.75 | 12.45 | serum |
| 2019 | Su Y [128] | 90 | 270.01 | 15.80 | 60 | 106.81 | 10.98 | serum |
| 2009 | Ueno K [129] | 80 | 964 | 551 | 26 | 439 | 189 | serum |
| 2016 | Zeng H [130] | 52 | 231.26 | 75.31 | 28 | 43.94 | 19.58 | plasma |
|  |  | KD | |  | FC | | |  |
| Year | Author | N1 | Mean1 | SD1 | N2 | Mean2 | SD2 | Sample type |
| 2011 | Breunis WB [124] | 101 | 811 | 532.6 | 20 | 554 | 411.4 | serum |
| 1998 | Maeno N [126] | 20 | 321.9 | 140.3 | 22 | 184.1 | 88.55 | serum |
| 1998 | Maeno N [126] | 13 | 320.1 | 200.2 | 22 | 184.1 | 88.55 | serum |
| 1999 | Ohno T [18] | 66 | 59.87 | 500.9 | 18 | 8.1 | 11.3 | serum |
| 1999 | Ohno T [18] | 31 | 62.50 | 500.9 | 18 | 8.1 | 11.3 | serum |
| 2019 | Su Y [128] | 90 | 270.01 | 15.80 | 40 | 205.83 | 21.04 | serum |
|  |  | Acute KD | | | Convalescent KD | | |  |
| Year | Author | N1 | Mean1 | SD1 | N2 | Mean2 | SD2 | Sample type |
| 2001 | Hamamichi Y [125] | 49 | 281 | 126 | 30 | 52 | 54.8 | serum |
| 1998 | Maeno N [126] | 20 | 321.9 | 140.3 | 15 | 156.7 | 77.48 | serum |
| 1999 | Ohno T [18] | 31 | 62.50 | 500.9 | 31 | 26.90 | 36.63 | serum |
| 2002 | Ohno T [18] | 41 | 68.0 | 500.9 | 41 | 58.4 | 82.18 | serum |

| **Table S1 Circulating VEGF levels of the studies included in the meta-analysis** | | | | | | | | |
| --- | --- | --- | --- | --- | --- | --- | --- | --- |
|  |  | AS | | | HC | | |  |
| Year | Author | N1 | Mean1 | SD1 | N2 | Mean2 | SD2 | Sample type |
| 2016 | Akar S [131] | 98 | 1591.71 | 753.55 | 49 | 995.70 | 611.23 | serum |
| 2016 | Deveci K [55] | 30 | 316.4 | 97.1 | 30 | 185.7 | 83.9 | serum |
| 2002 | Goldberger C [132] | 16 | 75.3 | 19.0 | 8 | 13.8 | 4.7 | EDTA plasma |
| 2015 | Lin TT [133] | 140 | 311.6 | 284.0 | 90 | 94.0 | 84.4 | serum |
| 2016 | Przepiera-Bedzak H [134] | 80 | 396.1 | 336.6 | 21 | 238.1 | 156.6 | serum |
| 2015 | Przepiera-Bedzak H [[135] | 61 | 395.2 | 340.7 | 29 | 300.1 | 163.4 | serum |
| 2016 | Przepiera-Bedzak H [136] | 81 | 351.2 | 258.4 | 30 | 270.0 | 196.3 | serum |
| 2016 | Sakellariou GT [137] | 57 | 326.5 | 117.0 | 34 | 289.1 | 86.9 | serum |
| 2015 | Solmaz D [138] | 98 | 1591.71 | 753.55 | 49 | 995.70 | 611.23 | serum |
| 2018 | Solmaz D [139] | 97 | 1391.7 | 745.0 | 48 | 855.6 | 561.0 | serum |
| 2019 | Torres L [140] | 204 | 338.8 | 231.9 | 80 | 378.0 | 3584 | serum |
| 2010 | Tseng JC [73] | 50 | 616 | 310 | 50 | 482 | 312 | serum |

| **Table S1 Circulating VEGF levels of the studies included in the meta-analysis** | | | | | | | | |
| --- | --- | --- | --- | --- | --- | --- | --- | --- |
|  |  | IBD | | | HC | | |  |
| Year | Author | N1 | Mean1 | SD1 | N2 | Mean2 | SD2 | Sample type |
| 2014 | Algaba A [142] | 37 | 511.5 | 255.6 | 40 | 395.5 | 256.4 | serum |
| 2006 | Ferrante M [145] | 792 | 197 | 157.8 | 263 | 107 | 95.56 | serum |
| 2015 | Kleiner G [150] | 26 | 91.71 | 114.7 | 37 | 82.2 | 57.91 | serum |
| 2020 | deZoeten EF [155] | 17 | 276.4 | 302.6 | 17 | 99.0 | 210.3 | serum |
|  |  | Active CD | |  | Inactive CD | | |  |
| Year | Author | N1 | Mean1 | SD1 | N2 | Mean2 | SD2 | Sample type |
| 1999 | Griga T [146] | 12 | 501.2 | 273.8 | 7 | 309 | 212 | serum |
| 1998 | Griga T [147] | 17 | 698.0 | 349.8 | 14 | 248.2 | 100.7 | serum |
| 2003 | Kapsoritakis A [149] | 26 | 313 | 280.4 | 18 | 203 | 233.3 | serum |
| 2004 | Magro F [151] | 22 | 165.4 | 259.8 | 59 | 72.9 | 223.5 | serum |
| 2004 | Magro F [151] | 64 | 136.0 | 269.6 | 59 | 72.9 | 223.5 | serum |
| 1997 | Schurer-Maly CC [154] | 14 | 290.9 | 248.4 | 10 | 274.9 | 179.6 | serum |
|  |  | Active UC | | | Inactive UC | | |  |
| Year | Author | N1 | Mean1 | SD1 | N2 | Mean2 | SD2 | Sample type |
| 1999 | Griga T [146] | 4 | 1957.2 | 1426.3 | 5 | 440.5 | 118.5 | serum |
| 1998 | Griga T [147] | 5 | 624.2 | 314.7 | 10 | 113.0 | 80.5 | serum |
| 2003 | Kapsoritakis A [149] | 27 | 228 | 187.1 | 23 | 302 | 239.8 | serum |
| 1997 | Schurer-Maly CC [154] | 12 | 303.4 | 192.6 | 11 | 162.4 | 80.26 | serum |
| 2004 | Magro F [151] | 25 | 127.4 | 348.5 | 27 | 98.8 | 392.3 | serum |
| 2004 | Magro F [151] | 21 | 100.2 | 249.3 | 27 | 98.8 | 392.3 | serum |

| **Table S1 Circulating VEGF levels of the studies included in the meta-analysis** | | | | | | | | |
| --- | --- | --- | --- | --- | --- | --- | --- | --- |
|  |  | PsA | | | HC | | |  |
| Year | Author | N1 | Mean1 | SD1 | N2 | Mean2 | SD2 | Sample type |
| 2009 | Ablin JN [14] | 10 | 509.37 | 357.88 | 15 | 363.66 | 278.91 | Not mentioned |
| 2009 | Ablin JN [14] | 22 | 409.03 | 313.22 | 15 | 363.66 | 278.91 | Not mentioned |
| 2007 | Akman A [157] | 46 | 388.3 | 249.9 | 20 | 357.7 | 321.1 | serum |
| 2010 | Anderson KS [158] | 14 | 275.45 | 351 | 14 | 140.85 | 112.8 | serum |
| 2001 | Ballara S [12] | 13 | 378 | 186 | 31 | 161 | 110 | serum |
| 2016 | Batycka-Baran A [159] | 24 | 188.6 | 127.6 | 26 | 94.45 | 47.77 | Not mentioned |
| 2016 | Batycka-Baran A [160] | 63 | 135.5 | 100.0 | 31 | 92.0 | 47.77 | plasma |
| 2016 | Capkin AA [161] | 48 | 346.74 | 213 | 48 | 243.8 | 118 | serum |
| 1999 | Bhushan M [162] | 15 | 16.57 | 15.53 | 13 | 7.81 | 3.71 | serum |
| 2002 | Creamer D [163] | 22 | 257 | 229.8 | 17 | 24.7 | 27.6 | plasma |
| 2010 | Flisiak I [164] | 59 | 444.9 | 314.9 | 20 | 228.1 | 82.3 | serum |
| 2007 | Fink AM [165] | 28 | 394 | 171.8 | 9 | 214.3 | 162.1 | serum |
| 2012 | Kaur S [166] | 58 | 193.26 | 124.29 | 58 | 121.82 | 69.90 | serum |
| 2014 | Meki AR [167] | 58 | 357.5 | 114.54 | 22 | 229.3 | 78.89 | serum |
| 2020 | Midde HS [168] | 54 | 211.36 | 26.67 | 54 | 183.07 | 59.94 | plasma |
| 2002 | Nielsen HJ [169] | 16 | 51 | 44.07 | 13 | 33 | 4.444 | plasma |
| 2008 | Nofal A [170] | 30 | 327 | 66.2 | 10 | 178 | 83.4 | serum |
| 2015 | Przepiera-Bedzak H [135] | 69 | 291.4 | 331.1 | 29 | 300.1 | 163.4 | serum |
| 2016 | Przepiera-Bedzak H [136] | 76 | 343.5 | 330.5 | 30 | 270.0 | 196.3 | serum |
| 2013 | Przepiera-Bedzak H [171] | 80 | 288.6 | 265 | 20 | 300.1 | 163.4 | serum |
| 2016 | Shahidi-Dadras M [172] | 60 | 78.7 | 61.01 | 60 | 12.65 | 7.733 | serum |
| 2016 | Shahidi-Dadras M [173] | 58 | 73.7 | 57.04 | 60 | 12.7 | 7.704 | serum |
| 2009 | Takahashi H [174] | 122 | 211 | 30.5 | 78 | 144 | 20.3 | serum |
| 2017 | Zheng YZ [175] | 194 | 332.56 | 82.15 | 175 | 94.63 | 17.58 | serum |
|  |  | Psoriasis athritis | | | HC | | |  |
| Year | Author | N1 | Mean1 | SD1 | N2 | Mean2 | SD2 | Sample type |
| 2009 | Ablin JN [14] | 22 | 409.03 | 313.22 | 15 | 363.66 | 278.91 | Not mentioned |
| 2001 | Ballara S [12] | 13 | 378 | 186 | 31 | 161 | 110 | serum |
| 2016 | Batycka-Baran A [159] | 24 | 188.6 | 127.6 | 26 | 94.45 | 47.77 | Not mentioned |
| 2002 | Creamer D [163] | 10 | 277 | 167.6 | 17 | 24.7 | 27.6 | plasma |
| 2007 | Fink AM [165] | 14 | 394 | 171.8 | 9 | 214.3 | 162.1 | serum |
| 2015 | Przepiera-Bedzak H [135] | 69 | 291.4 | 331.1 | 29 | 300.1 | 163.4 | serum |
| 2016 | Przepiera-Bedzak H [136] | 76 | 343.5 | 330.5 | 30 | 270.0 | 196.3 | serum |
| 2013 | Przepiera-Bedzak H [171] | 80 | 288.6 | 265 | 20 | 300.1 | 163.4 | serum |
|  |  | Skin psoriasis | | | HC | | |  |
| Year | Author | N1 | Mean1 | SD1 | N2 | Mean2 | SD2 | Sample type |
| 2009 | Ablin JN [14] | 10 | 509.37 | 357.88 | 15 | 363.66 | 278.91 | Not mentioned |
| 2010 | Anderson KS [158] | 14 | 275.45 | 351 | 14 | 140.85 | 112.8 | serum |
| 2016 | Batycka-Baran A [160] | 63 | 135.5 | 100.0 | 31 | 92.0 | 47.77 | plasma |
| 2010 | Flisiak I [164] | 59 | 444.9 | 314.9 | 20 | 228.1 | 82.3 | serum |
| 2012 | Kaur S [166] | 58 | 193.26 | 124.29 | 58 | 121.82 | 69.90 | serum |
| 2014 | Meki AR [167] | 58 | 357.5 | 114.54 | 22 | 229.3 | 78.89 | serum |
| 2002 | Nielsen HJ [169] | 16 | 51 | 44.07 | 13 | 33 | 4.444 | plasma |
| 2008 | Nofal A [170] | 30 | 327 | 66.2 | 10 | 178 | 83.4 | serum |
| 2016 | Shahidi-Dadras M [172] | 60 | 78.7 | 61.01 | 60 | 12.65 | 7.733 | serum |
| 2016 | Shahidi-Dadras M [173] | 58 | 73.7 | 57.04 | 60 | 12.7 | 7.704 | serum |
| 2017 | Zheng YZ [175] | 194 | 332.56 | 82.15 | 175 | 94.63 | 17.58 | serum |

| **Table S1 Circulating VEGF levels of the studies included in the meta-analysis** | | | | | | | | |
| --- | --- | --- | --- | --- | --- | --- | --- | --- |
|  |  | GD | | | HC | | |  |
| Year | Author | N1 | Mean1 | SD1 | N2 | Mean2 | SD2 | Sample type |
| 2020 | Cheng CW [10] | 40 | 54.18 | 112.56 | 14 | 56.45 | 84.58 | serum |
| 2009 | Figueroa-Vega N [176] | 44 | 377.75 | 334.81 | 22 | 308.9 | 170.9 | serum |
| 1998 | Iitaka M [177] | 49 | 261 | 157 | 37 | 130 | 85 | serum |
| 1998 | Iitaka M [177] | 49 | 196 | 124 | 37 | 130 | 85 | serum |
| 2014 | Kajdaniuk D [178] | 16 | 463.12 | 330.45 | 22 | 236.11 | 106.68 | serum |
| 2016 | Rancier M [179] | 21 | 108.00 | 171.63 | 55 | 87.98 | 5.50 | plasma |
| 2014 | Ye X [180] | 30 | 125.46 | 34.82 | 30 | 76.45 | 6.81 | serum |
|  |  | Active GO | |  | Inactive GO | | |  |
| Year | Author | N1 | Mean1 | SD1 | N2 | Mean2 | SD2 | Sample type |
| 2009 | Figueroa-Vega N [176] | 13 | 589.70 | 517.59 | 13 | 235.23 | 152.50 | serum |
| 2014 | Ye X [180] | 34 | 182.76 | 75.17 | 14 | 132.34 | 42.19 | serum |

| **Table S2 Correlation analysis of the studies included in the SLE** | | | | | | | |
| --- | --- | --- | --- | --- | --- | --- | --- |
| Variable | Year | Author | n | Spearman r | Pearson r | Fisher's Z | SE |
| SLEDAI | 2012 | Edelbauer M [31] | 23 | 0.486 | 0.503 | 0.554 | 0.224 |
|  | 2018 | El-Gazzar II [32] | 84 | 0.340 | 0.354 | 0.370 | 0.111 |
|  | 2017 | Willis R [50] | 267 |  | 0.330 | 0.343 | 0.062 |
|  | 2014 | Zhou L [51] | 54 |  | 0.385 | 0.406 | 0.140 |
|  | 2007 | Heshmat NM [34] | 25 |  | 0.860 | 1.293 | 0.213 |
| SLAM | 2002 | Robak E [49] | 52 | 0.275 | 0.287 | 0.295 | 0.143 |
|  | 2009 | Elhelaly NS [30] | 23 |  | 0.670 | 0.811 | 0.224 |
|  |  |  |  |  |  |  |  |
| C3 | 2009 | Elhelaly NS [30] | 23 |  | -0.540 | -0.604 | 0.224 |
|  | 2007 | Heshmat NM [34] | 25 |  | -0.620 | -0.725 | 0.213 |
|  | 2014 | Zhou L [51] | 54 | -0.108 | -0.113 | -0.114 | 0.140 |
|  |  |  |  |  |  |  |  |
| ESR | 2009 | Elhelaly NS [30] | 23 |  | 0.490 | 0.536 | 0.224 |
|  | 2014 | Guoyuan Lu[51] | 54 |  | 0.527 | 0.586 | 0.140 |
|  | 2018 | El-Gazzar II [32] | 84 | 0.190 | 0.199 | 0.201 | 0.111 |
|  |  |  |  |  |  |  |  |
| Platelet count | 2009 | Elhelaly NS [30] | 23 |  | -0.510 | -0.563 | 0.224 |
|  | 2007 | Heshmat NM [34] | 25 |  | -0.480 | -0.523 | 0.213 |
|  | 2018 | El-Gazzar II [32] | 84 | 0.040 | 0.042 | 0.042 | 0.111 |

| Variable | Year | Author | n | Spearman r | Pearson r | Fisher's Z | SE |
| --- | --- | --- | --- | --- | --- | --- | --- |
| DAS-28 | 2008 | Foster W [22] | 66 | 0.577 | 0.595 | 0.685 | 0.126 |
|  | 2006 | Kuryliszyn-Moskal A [63] | 64 | 0.390 | 0.406 | 0.430 | 0.128 |
|  | 2001 | Ballara S [12] | 44 |  | 0.404 | 0.428 | 0.156 |
|  | 2010 | Milman N [65] | 47 | 0.142 | 0.149 | 0.150 | 0.151 |
|  | 2010 | Milman N [65] | 47 | 0.241 | 0.252 | 0.257 | 0.151 |
|  | 2010 | Milman N [65] | 47 | 0.228 | 0.238 | 0.243 | 0.151 |
|  | 2010 | Milman N [65] | 47 | 0.275 | 0.287 | 0.295 | 0.151 |
|  | 2016 | do Prado AD [57] | 64 | 0.180 | 0.188 | 0.190 | 0.128 |
|  | 2016 | do Prado AD [57] | 57 | 0.180 | 0.188 | 0.190 | 0.136 |
|  |  |  |  |  |  |  |  |
| CRP | 2008 | Foster W [22] | 66 | 0.250 | 0.261 | 0.267 | 0.126 |
|  | 2016 | Gumus A [58] | 59 | 0.370 | 0.385 | 0.406 | 0.134 |
|  | 2001 | Ballara S [12] | 78 |  | 0.499 | 0.548 | 0.115 |
|  | 2001 | Ballara S [12] | 44 | 0.287 | 0.299 | 0.309 | 0.156 |
|  | 2001 | Sone H [24] | 155 |  | 0.580 | 0.662 | 0.081 |
|  | 2010 | Milman N [65] | 47 | 0.140 | 0.146 | 0.148 | 0.151 |
|  | 2016 | do Prado AD [57] | 57 | 0.140 | 0.146 | 0.148 | 0.136 |
|  |  |  |  |  |  |  |  |
| ESR | 2008 | Foster W [22] | 66 | 0.230 | 0.240 | 0.245 | 0.126 |
|  | 2016 | Gumus A [58] | 59 | 0.330 | 0.344 | 0.358 | 0.134 |
|  | 2006 | Kuryliszyn-Moskal A [63] | 64 | 0.564 | 0.582 | 0.666 | 0.128 |
|  | 2001 | Ballara S [12] | 44 |  | 0.258 | 0.264 | 0.156 |
|  | 2001 | Sone H [24] | 155 |  | 0.534 | 0.596 | 0.081 |
|  | 2010 | Milman N [65] | 47 | 0.006 | 0.006 | 0.006 | 0.151 |
|  | 2016 | do Prado AD [57] | 64 | 0.070 | 0.073 | 0.073 | 0.128 |
|  | 2010 | Ozgonenel L [68] | 40 |  | 0.445 | 0.478 | 0.164 |

**Table S2 Correlation analysis of the studies included in the RA**

| **Table S2 Correlation analysis of the studies included in the SSc** | | | | | | | |
| --- | --- | --- | --- | --- | --- | --- | --- |
| Variable | Year | Author | n | Spearman r | Pearson r | Fisher's Z | SE |
| mRSS | 2016 | Shenavandeh S [106] | 44 |  | 0.310 | 0.321 | 0.156 |
|  | 2017 | Saranya C [105] | 55 | 0.717 | 0.733 | 0.935 | 0.139 |
|  | 2013 | Farouk HM [90] | 25 | 0.290 | 0.303 | 0.312 | 0.213 |
|  | 2012 | Dunne JV [87] | 14 | -0.700 | -0.717 | -0.901 | 0.302 |
|  | 2002 | Choi JJ [11] | 48 | 0.656 | 0.674 | 0.817 | 0.149 |
|  |  |  |  |  |  |  |  |
| PAP | 2009 | Papaioannou AI [101] | 40 |  | 0.578 | 0.659 | 0.164 |
|  | 2012 | Dunne JV [87] | 26 | 0.490 | 0.508 | 0.559 | 0.209 |
|  |  |  |  |  |  |  |  |
| MRC dyspnea score | 2017 | Saranya C [105] | 55 | 0.717 | 0.733 | 0.936 | 0.139 |
|  | 2009 | Papaioannou AI [101] | 40 |  | 0.341 | 0.355 | 0.164 |

| **Table S2 Correlation analysis of the studies included in the BD** | | | | | | | |
| --- | --- | --- | --- | --- | --- | --- | --- |
| Variable | Year | Author | n | Spearman r | Pearson r | Fisher's Z | SE |
| BDCAF | 2013 | Eldin AB [113] | 18 | 0.783 | 0.797 | 1.091 | 0.258 |
|  | 2013 | Eldin AB [113] | 12 | 0.981 | 0.983 | 2.371 | 0.333 |
|  | 2012 | Ganeb SS [115] | 70 |  | 0.278 | 0.286 | 0.122 |
|  |  |  |  |  |  |  |  |
| ESR |  |  |  |  |  |  |  |
| p＞0.05 | 2012 | Ganeb SS [115] | 70 |  | 0.290 | 0.299 | 0.122 |
|  | 2011 | Ibrahim SE [117] | 40 |  | 0.582 | 0.665 | 0.164 |

| **Table S2 Correlation analysis of the studies included in the AS** | | | | | | | |
| --- | --- | --- | --- | --- | --- | --- | --- |
| Variable | Year | Author | n | Spearman r | Pearson r | Fisher's Z | SE |
| CRP | 2016 | Sakellariou GT [137] | 57 | 0.285 | 0.297 | 0.307 | 0.136 |
|  | 2018 | Solmaz D [139] | 97 | 0.284 | 0.296 | 0.305 | 0.103 |
|  | 2019 | L. Torres[140] | 204 | 0.188 | 0.197 | 0.199 | 0.071 |
|  |  |  |  |  |  |  |  |
| ESR | 2016 | Przepiera-Bedzak H [134] | 80 | 0.210 | 0.219 | 0.223 | 0.114 |
|  | 2016 | Sakellariou GT [137] | 57 | 0.284 | 0.296 | 0.305 | 0.136 |
|  | 2018 | Solmaz D [139] | 97 | 0.270 | 0.282 | 0.290 | 0.103 |
|  | 2019 | Torres L [140] | 204 | 0.236 | 0.247 | 0.252 | 0.071 |
|  |  |  |  |  |  |  |  |
| BASDAI | 2016 | Sakellariou GT [137] | 57 | 0.349 | 0.363 | 0.381 | 0.136 |
| BASMI | 2002 | Goldberger C [132] | 13 | 0.665 | 0.682 | 0.834 | 0.316 |
| BASMI | 2016 | Przepiera-Bedzak H [134] | 80 | 0.210 | 0.219 | 0.223 | 0.114 |
| ASDAS-CRP | 2018 | Solmaz D [139] | 97 | 0.235 | 0.245 | 0.251 | 0.103 |

| **Table S2 Correlation analysis of the studies included in the IBD** | | | | | | | | |
| --- | --- | --- | --- | --- | --- | --- | --- | --- |
| Variable | Year | Author | n | Spearman r | Pearson r | Fisher's Z | | SE |
| UCAI | 2018 | Aksoy EK [141] | 39 | 0.459 | 0.476 | 0.518 | 0.167 | |
|  | 1998 | Griga T [147] | 15 |  | 0.630 | 0.741 | 0.289 | |
|  |  |  |  |  |  |  |  | |
| CDAI | 2004 | Di Sabatino A [143] | 25 | 0.510 | 0.528 | 0.587 | 0.213 | |
|  | 1998 | Griga T [147] | 31 |  | 0.210 | 0.213 | 0.189 | |
| CDAI/CAI | 1997 | Schurer-Maly CC [154] | 24 |  | 0.230 | 0.234 | 0.218 | |
|  |  |  |  |  |  |  |  | |
| platelet count | 2018 | Aksoy EK [141] | 39 | 0.542 | 0.560 | 0.633 | 0.167 | |
|  | 2003 | Kapsoritakis A [149] | 50 |  | 0.260 | 0.266 | 0.146 | |
|  | 2003 | Kapsoritakis A [149] | 44 |  | 0.140 | 0.141 | 0.156 | |
|  | 2003 | Kapsoritakis A [149] | 50 |  | 0.080 | 0.080 | 0.146 | |
|  | 2003 | Kapsoritakis A [149] | 44 |  | 0.400 | 0.424 | 0.156 | |
|  | 2007 | Pousa ID [153] | 70 |  | 0.390 | 0.412 | 0.122 | |
|  |  |  |  |  |  |  |  | |
| ESR | 2018 | Aksoy EK [141] | 39 | 0.703 | 0.720 | 0.907 | 0.167 | |
|  | 1997 | Schurer-Maly CC [154] | 23 |  | 0.790 | 1.071 | 0.224 | |
|  | 1997 | Schurer-Maly CC [154] | 24 |  | 0.560 | 0.633 | 0.218 | |

| **Table S2 Correlation analysis of the studies included in the PsA** | | | | | | | |
| --- | --- | --- | --- | --- | --- | --- | --- |
| Variable | Year | Author | n | Spearman r | Pearson r | Fisher's Z | SE |
| PASI |  |  |  |  |  |  |  |
|  | 2009 | Ablin JN [14] | 32 |  | 0.218 | 0.222 | 0.186 |
|  | 2010 | Flisiak I [164] | 59 |  | 0.657 | 0.788 | 0.134 |
|  | 2014 | Meki AR [167] | 58 | 0.650 | 0.668 | 0.806 | 0.135 |
|  | 2020 | Midde HS [168] | 54 | 0.740 | 0.756 | 0.986 | 0.140 |
|  | 2008 | Nofal A [170] | 30 |  | 0.770 | 1.020 | 0.192 |
|  | 2008 | Nofal A [170] | 30 |  | 0.590 | 0.678 | 0.192 |
|  | 2016 | Shahidi-Dadras M [172] | 60 | 0.980 | 0.982 | 2.345 | 0.132 |
|  | 2016 | Shahidi-Dadras M [173] | 58 | 0.980 | 0.982 | 2.345 | 0.135 |
|  | 2009 | Takahashi H [174] | 122 | 0.671 | 0.688 | 0.845 | 0.092 |
|  |  |  |  |  |  |  |  |
| Duration of disease | 2016 | Shahidi-Dadras M [173] | 58 | 0.440 | 0.457 | 0.493 | 0.135 |
|  | 2016 | Shahidi-Dadras M [172] | 60 | 0.460 | 0.477 | 0.519 | 0.132 |


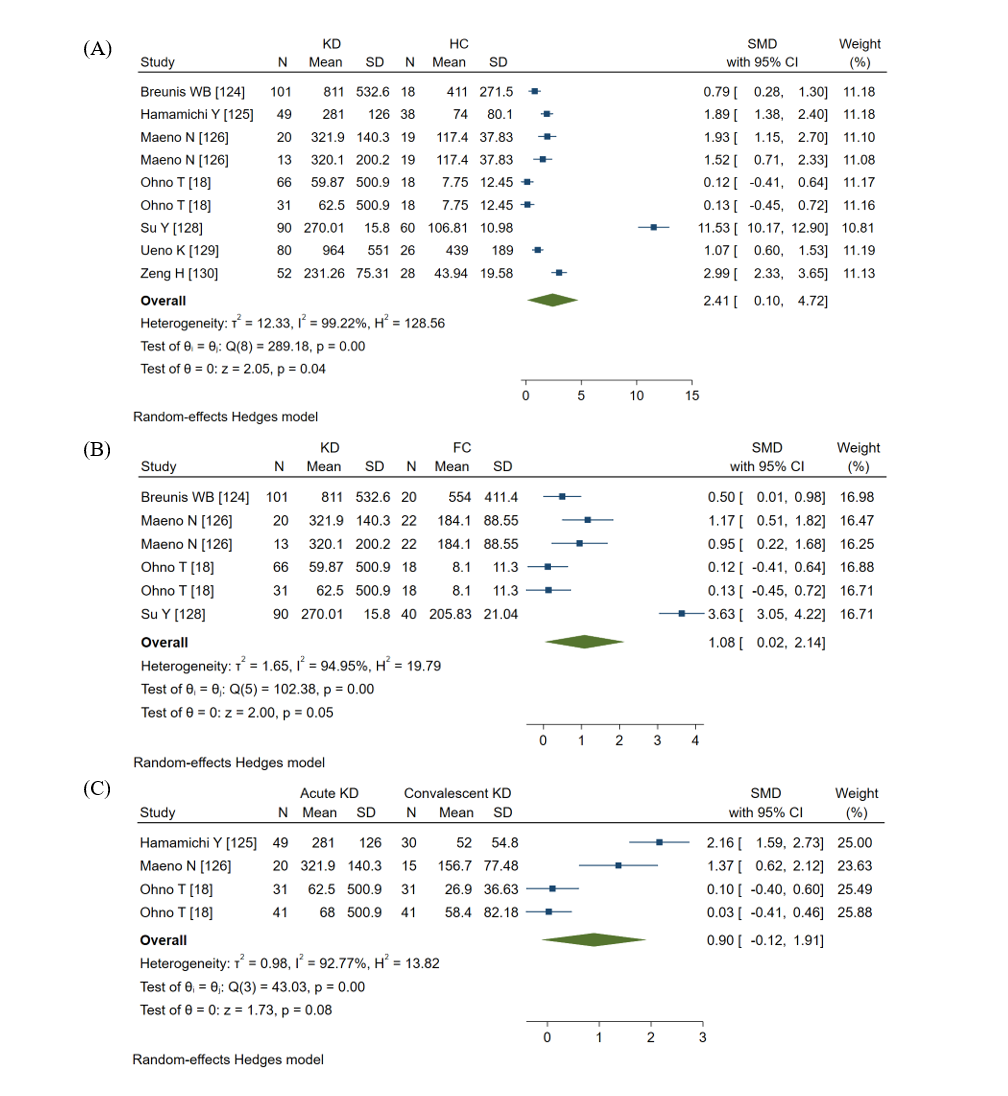

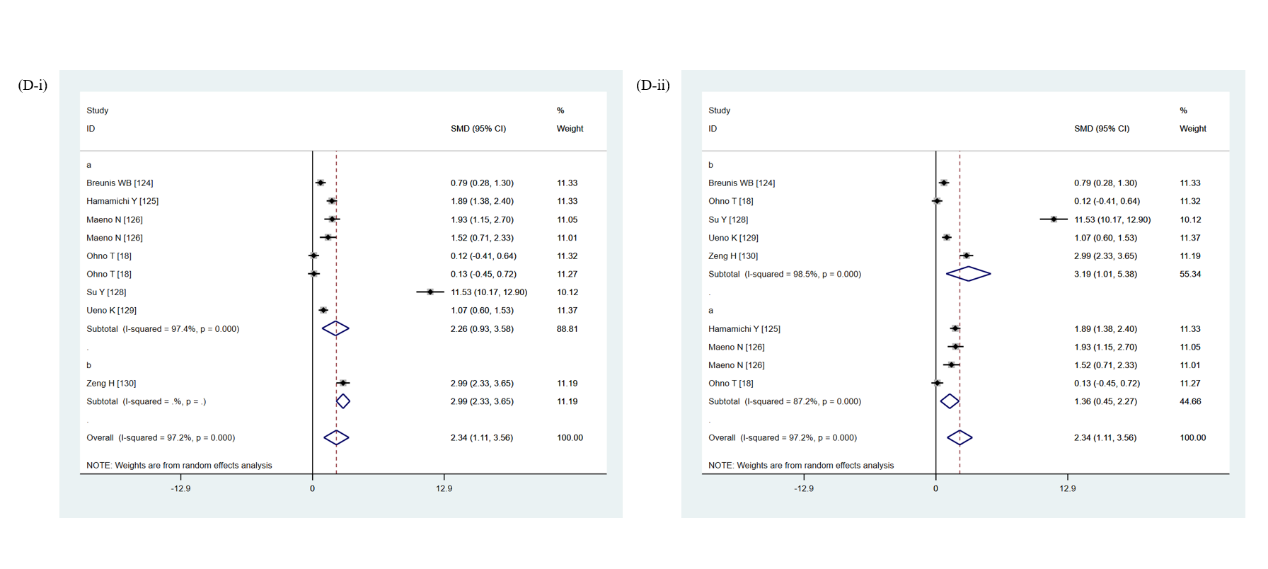


**Figure S1** Forest plot of KD associated with the circulating VEGF. **(A)** KD vs. HC, forest plot; **(B)** KD vs. FC, forest plot; **(C)** Acute KD vs. Convalescent KD, forest plot; **(D)** Subgroup analysis: **i.** Serum vs. Plasma (a for serum and b for plasma); **ii.** Sample size n≤50 vs. n>50 (a for n≤50 and b for n>50)


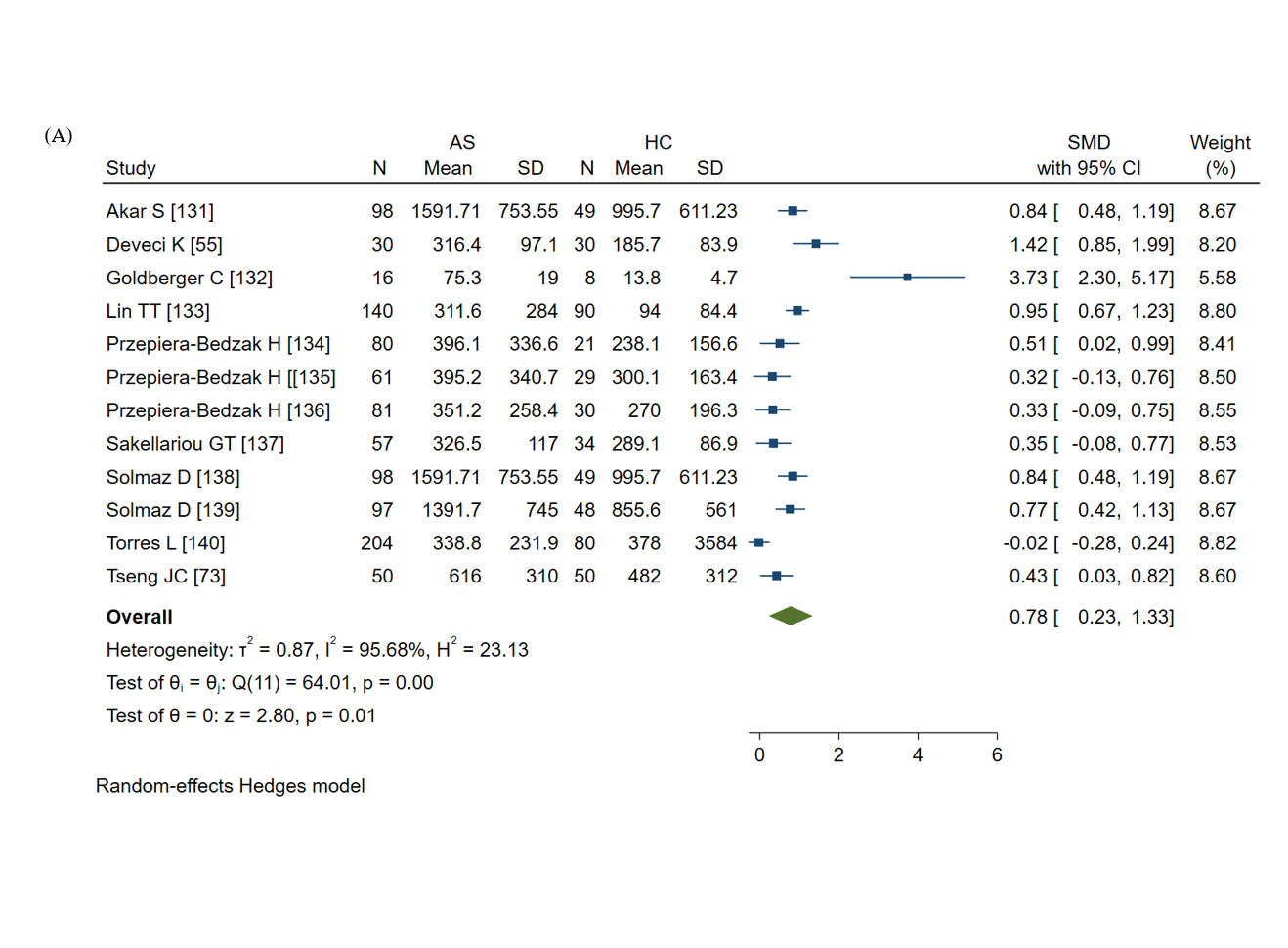


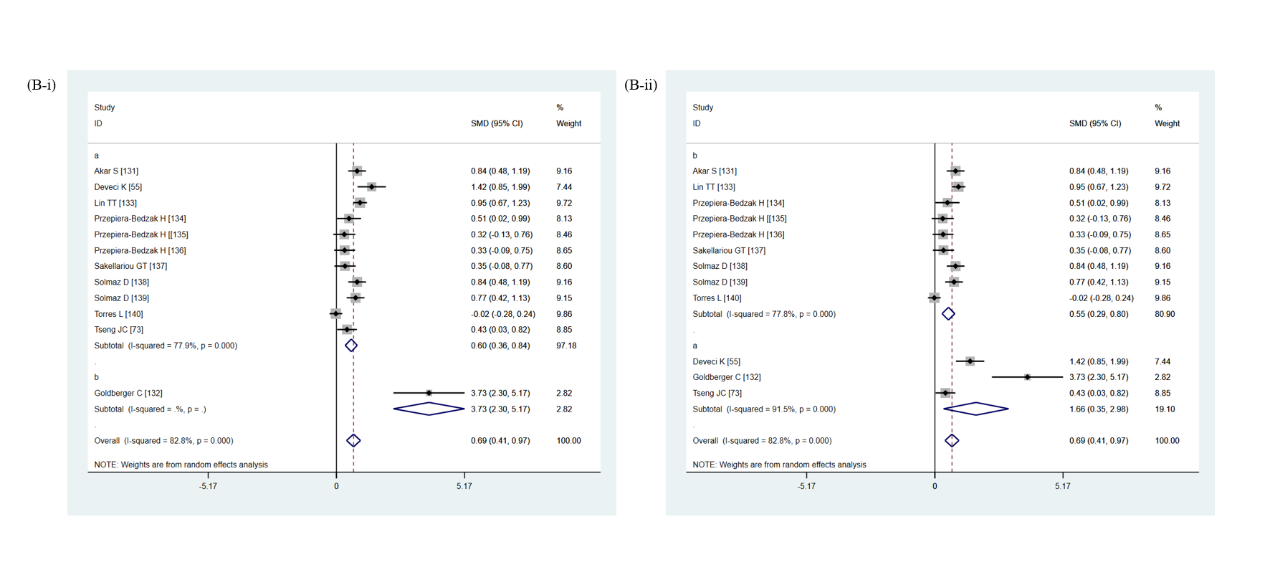


**Figure S2** Forest plot of AS associated with the circulating VEGF. **(A)** AS vs. HC, forest plot; **(B)** Subgroup analysis: **i.** Serum vs. Plasma (a for serum and b for plasma); **ii.** Sample size n≤50 vs. n>50 (a for n≤50 and b for n>50)


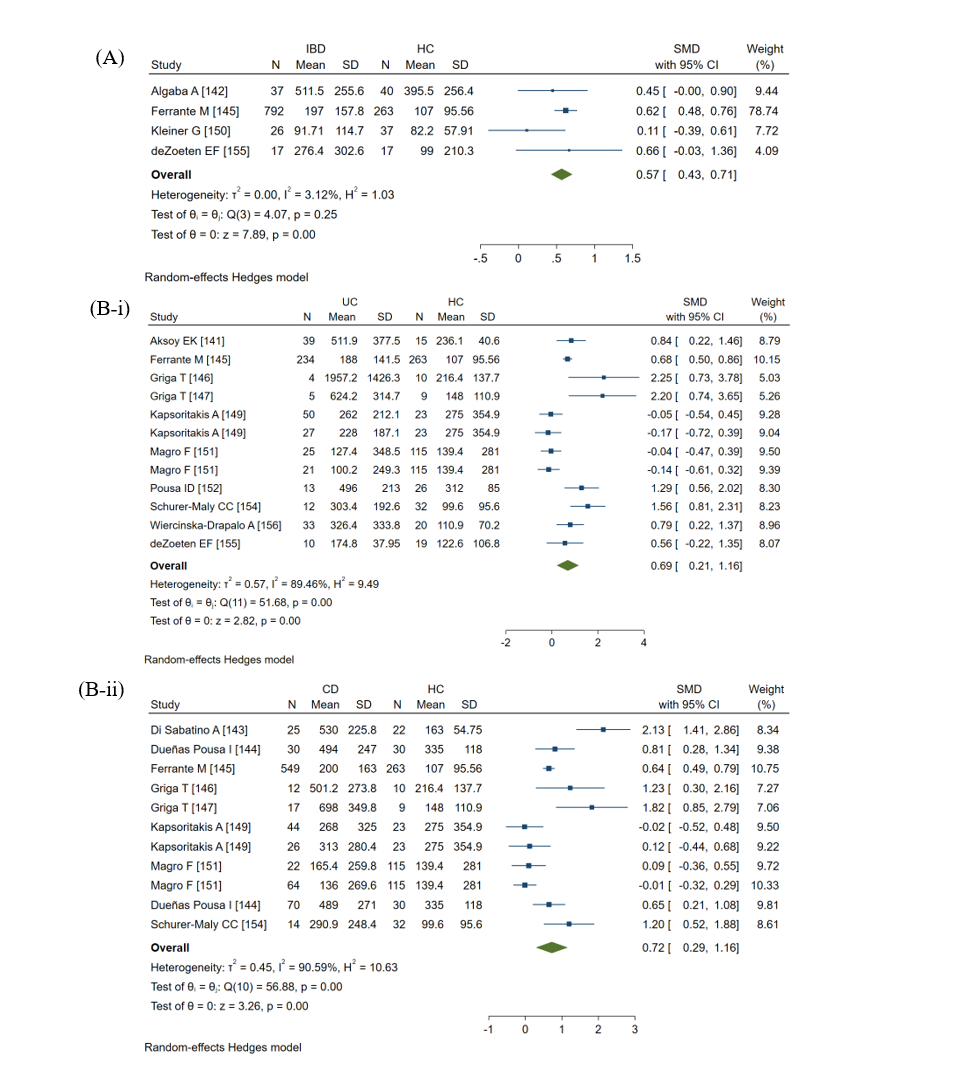

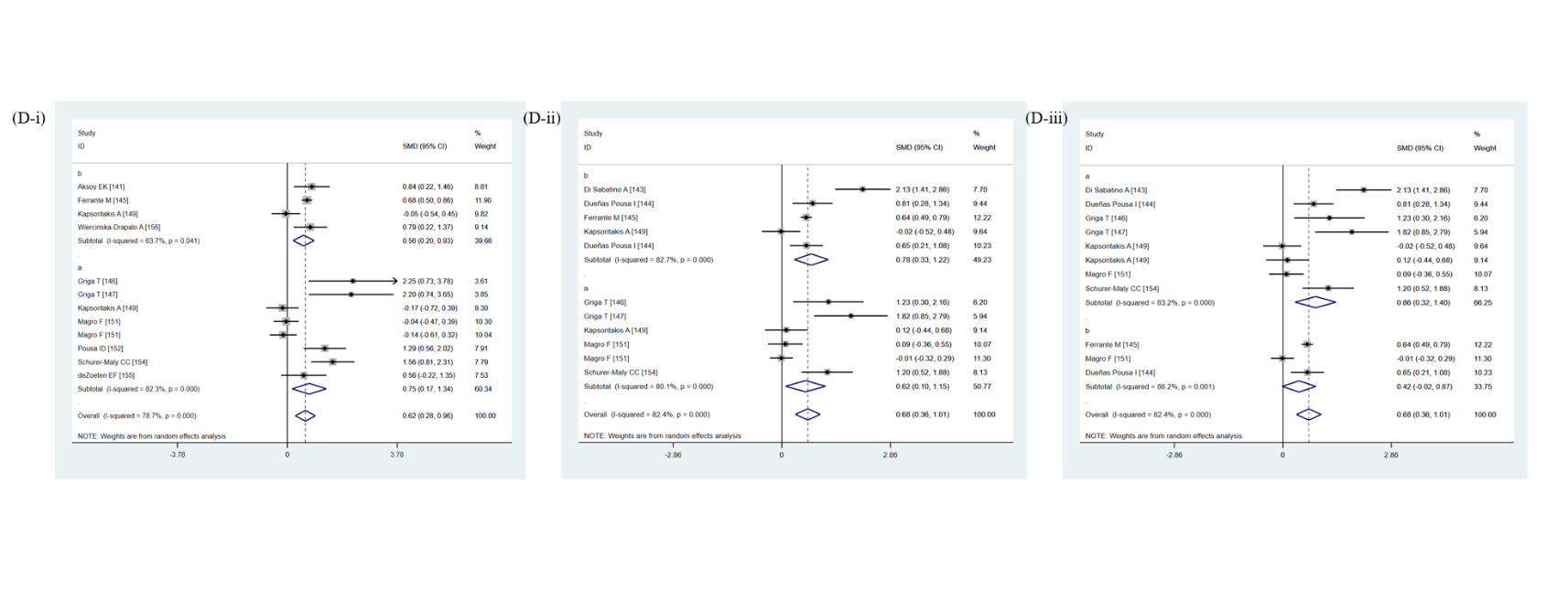

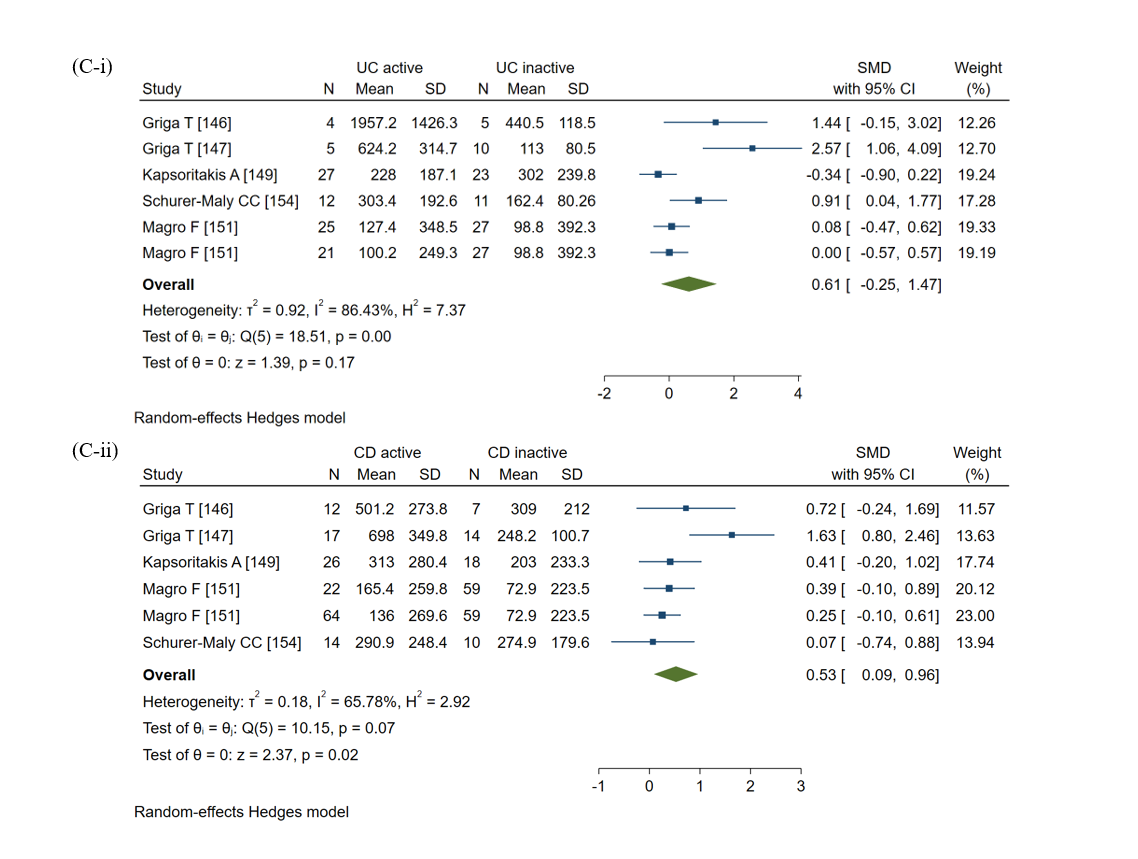


**Figure S3** Forest plot of IBD associated with the circulating VEGF. **(A)** IBD vs. HC, forest plot; **(B) i.** UC vs. HC; ii. CD vs. HC, forest plot; **(C) i.** UC active vs. inactive; **ii.** CD active vs. inactive, forest plot; **(D)** Subgroup analysis: **i.** Active UC vs. UC group without mentioning activity (a for active UC and b for UC group without mentioning activity); **ii.** Active CD vs. CD group without mentioning activity (a for active CD and b for CD group without mentioning activity); **iii.** Sample size n≤50 vs. n>50 (a for n≤50 and b for n>50)


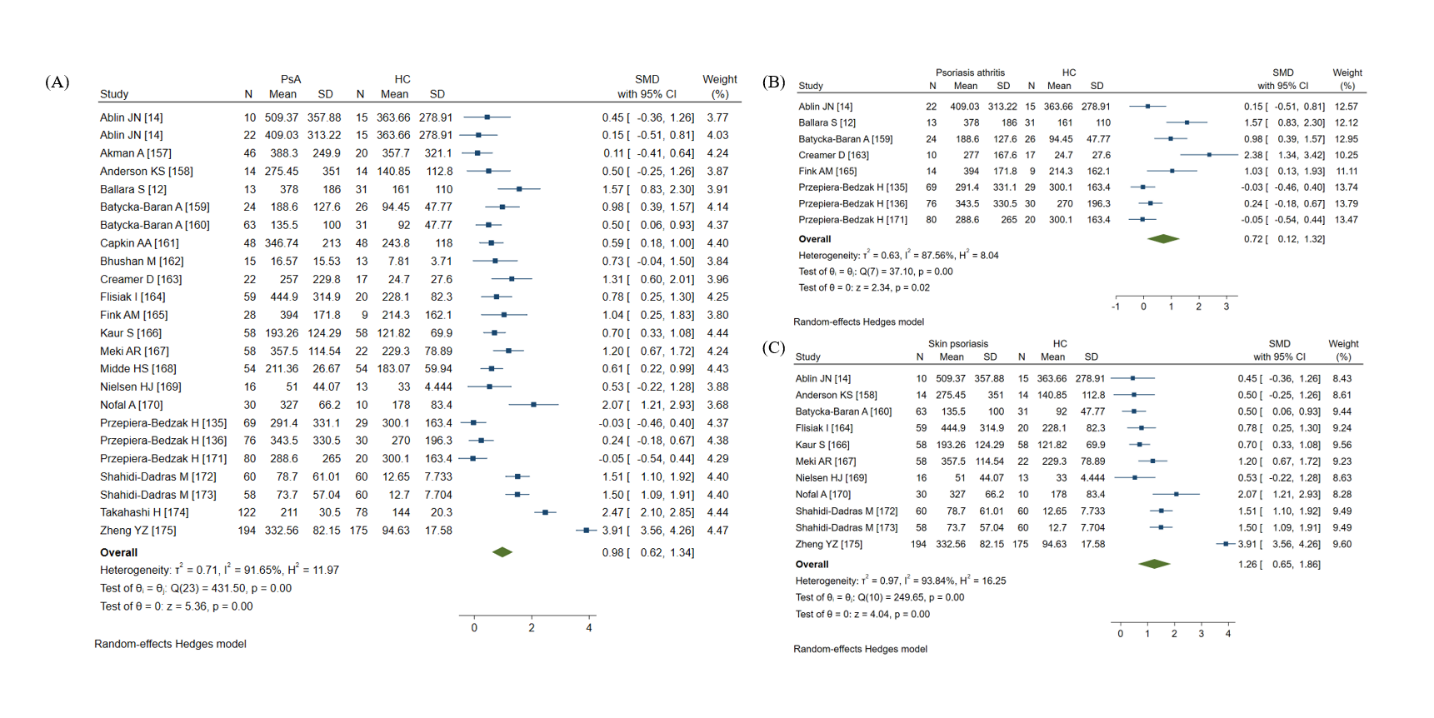

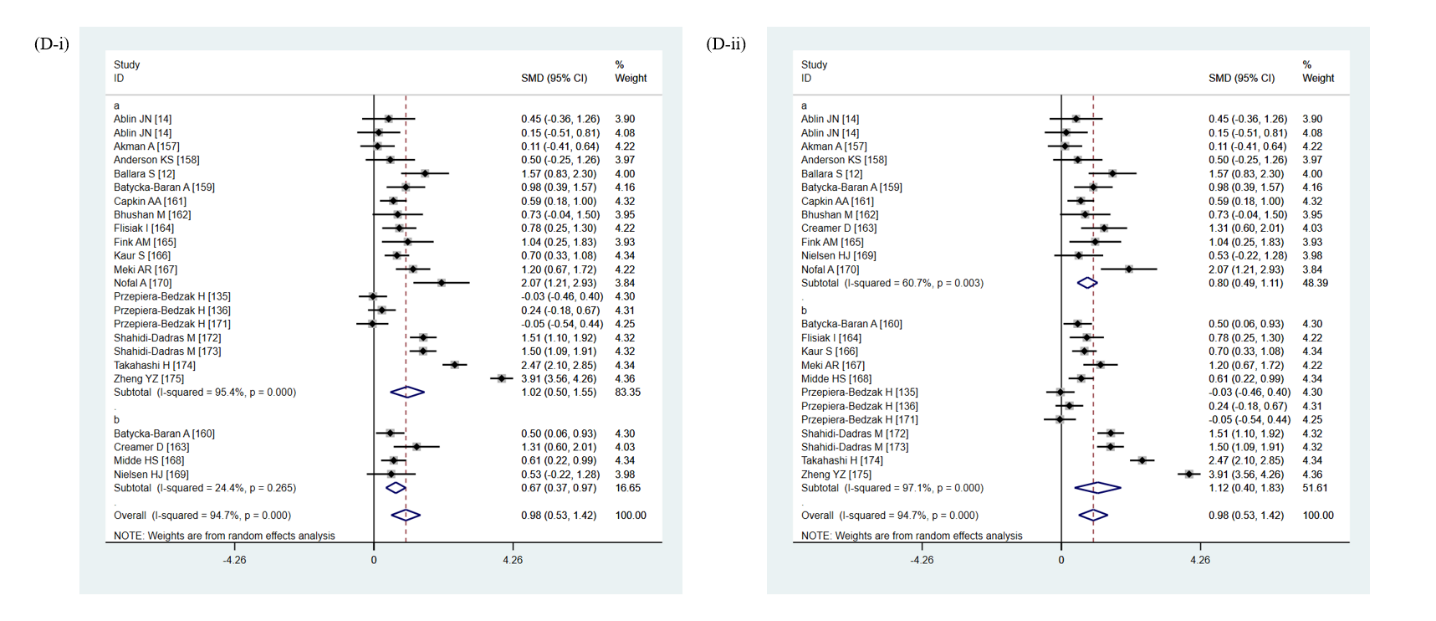


**Figure S4** Forest plot of PsA associated with the circulating VEGF. **(A)** PsA vs. HC, forest plot; **(B)** Psoriasis arthritis vs. HC, forest plot; **(C)** Skin psoriasis vs. HC, forest plot; **(D)** Subgroup analysis: **i.** Serum vs. Plasma (a for serum and b for plasma); **ii.** Sample size n≤50 vs. n>50 (a for n≤50 and b for n>50)


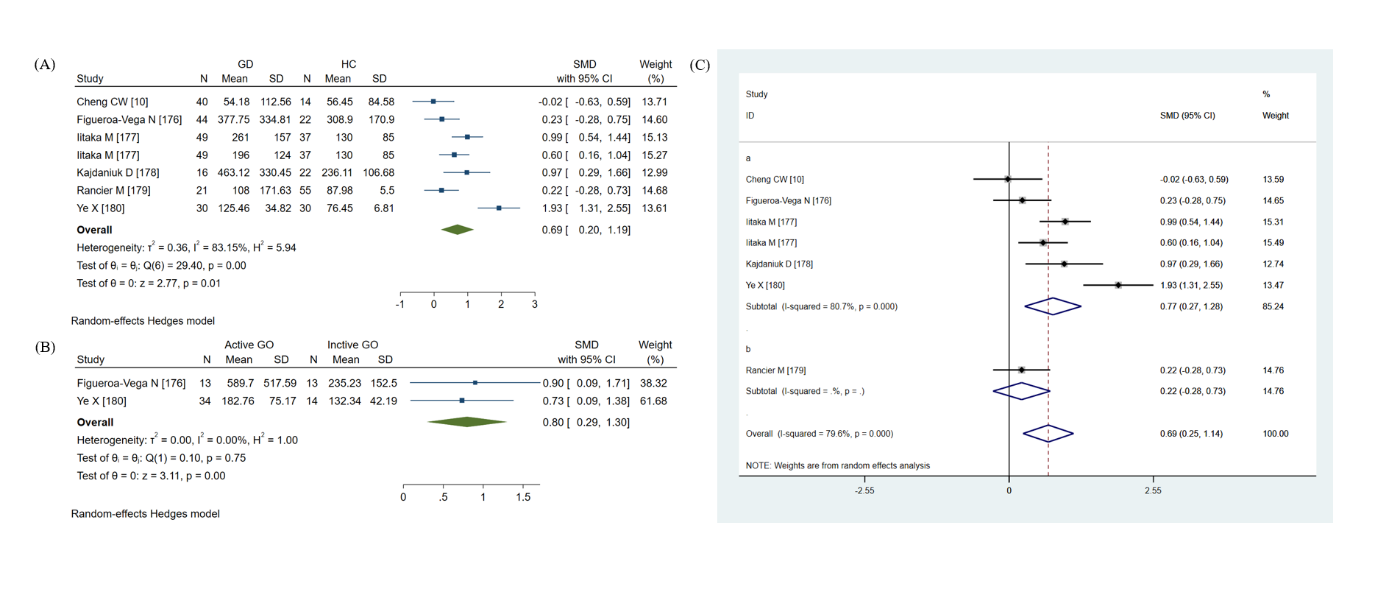


**Figure S5** Forest plot of GD associated with the circulating VEGF. **(A)** GD vs. HC, forest plot; **(B)** Active GO vs. Inactive GO, forest plot; **(C)** Subgroup analysis: Serum vs. Plasma (a for serum and b for plasma)

**
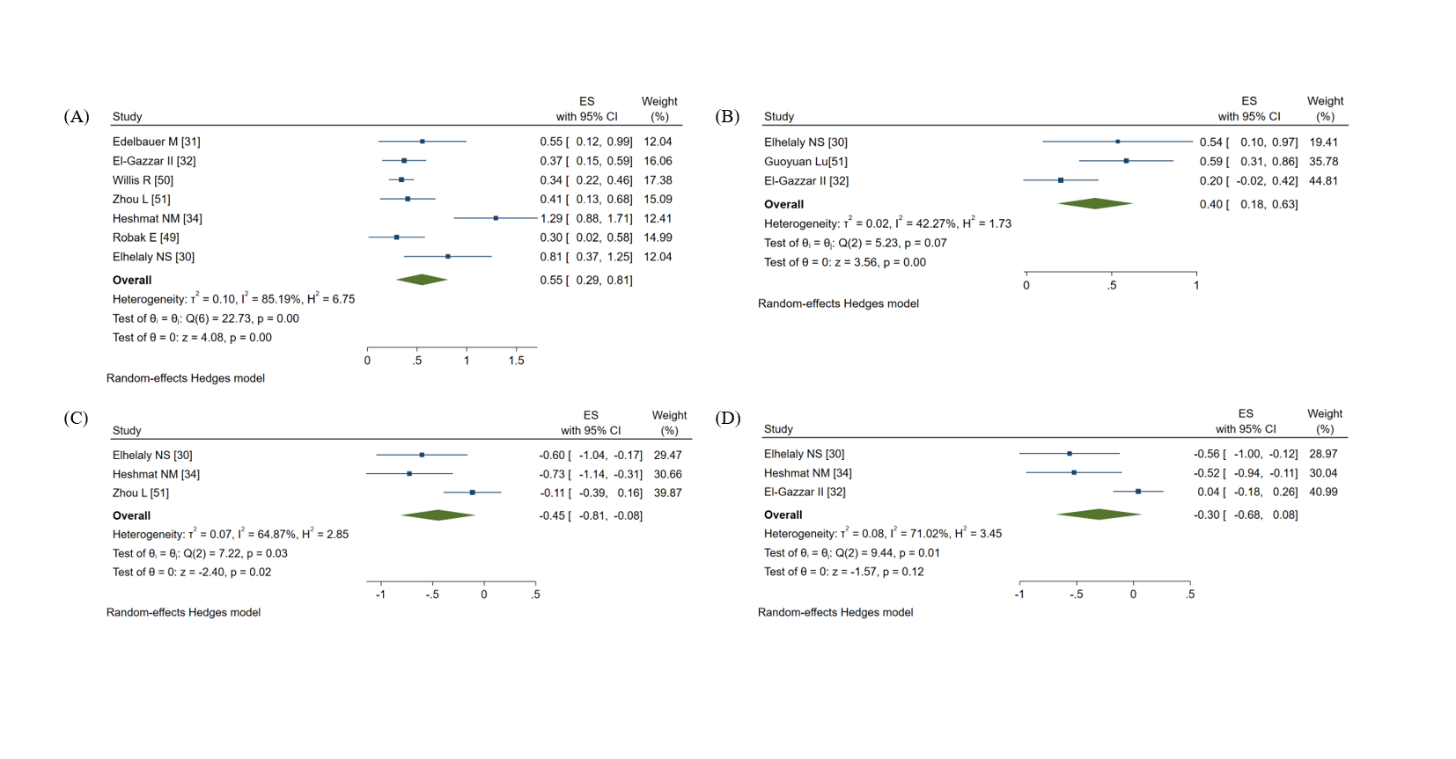
**

**Figure S6** Correlation analysis of SLE associated with the circulating VEGF. **(A)** disease activity: SLEDAI/SLAM, forest plot; **(B)** ESR, forest plot; **(C)** C3, forest plot; **(D)** platelet count, forest plot. ES: fisher’s z.


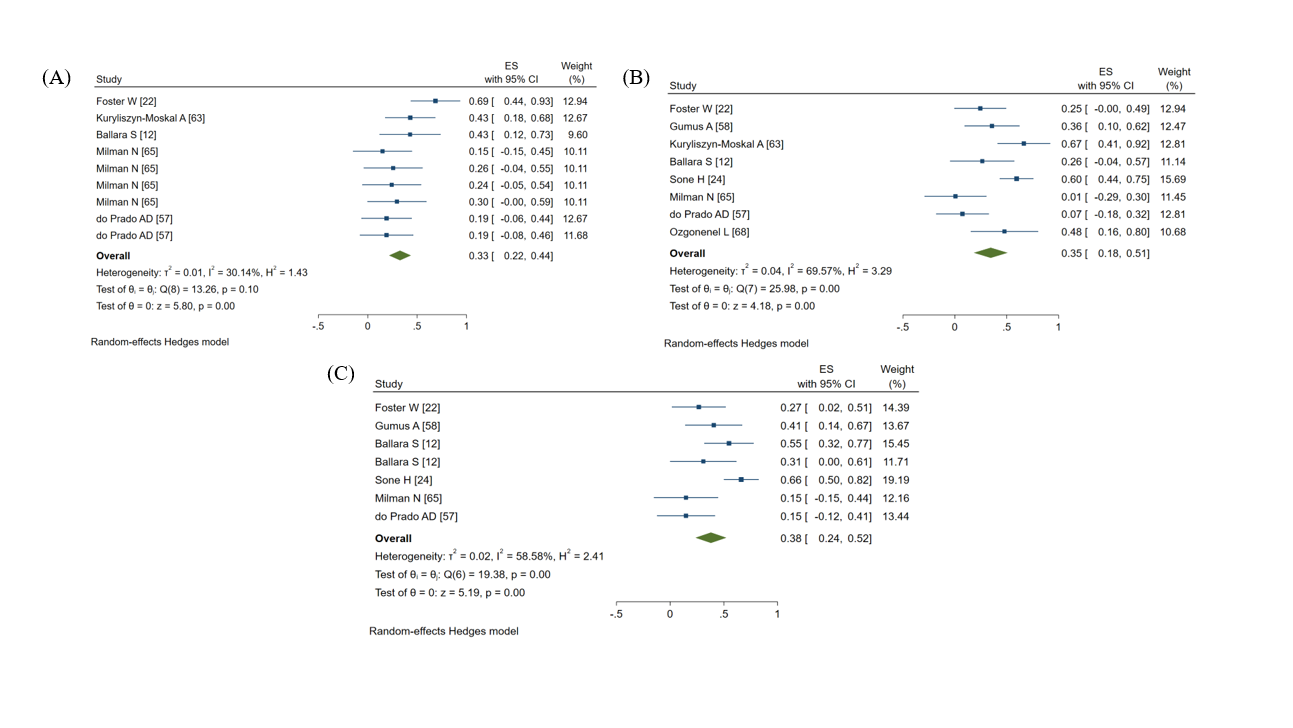


**Figure S7** Correlation analysis of RA associated with the circulating VEGF. **(A)** disease activity: DAS-28, forest plot; **(B)** ESR, forest plot; **(C)** CRP, forest plot. ES: fisher’s z.


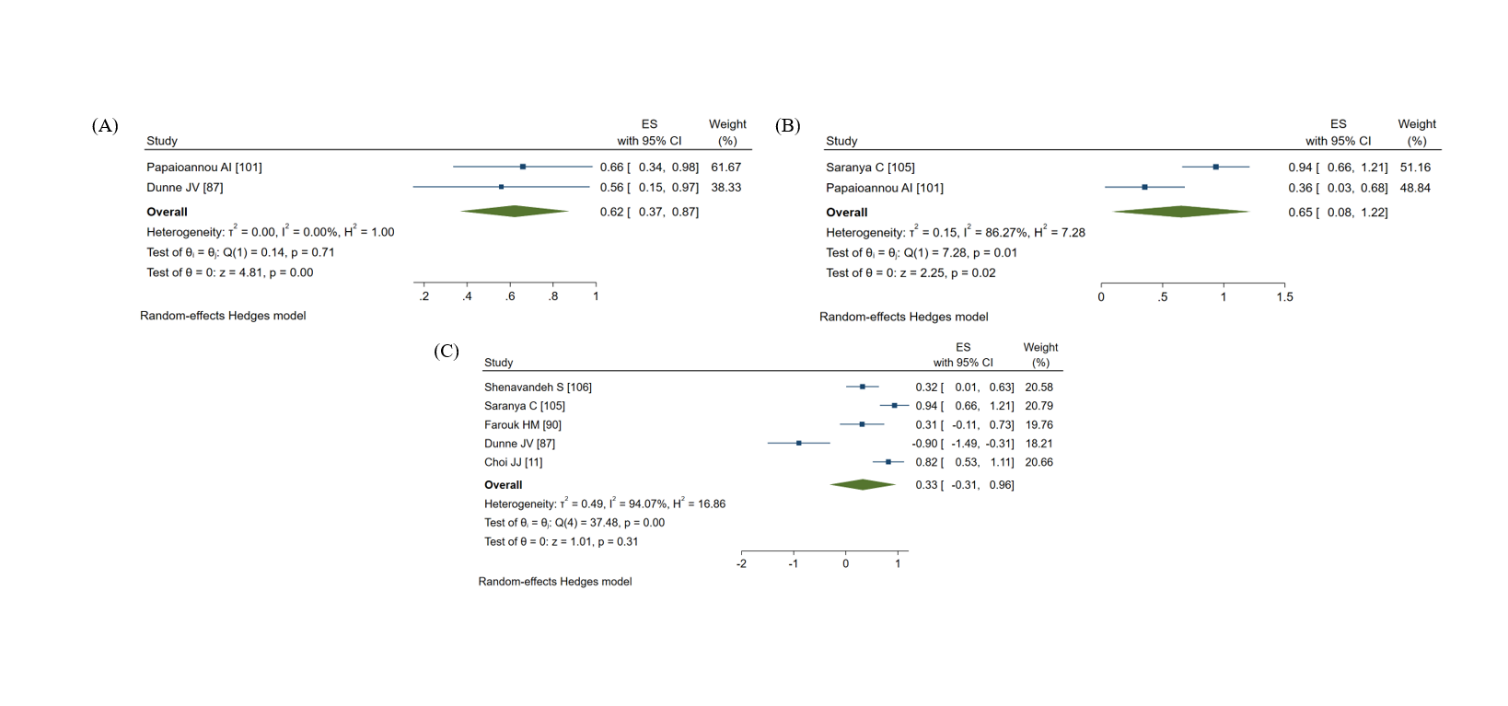


**Figure S8** Correlation analysis of SSc associated with the circulating VEGF. **(A)** pulmonary artery pressure (PAP), forest plot; **(B)** medical research council dyspnea score (MRC dyspnea score), forest plot; **(C)** modified Ronan skin score (mRSS), forest plot. ES: fisher’s z.


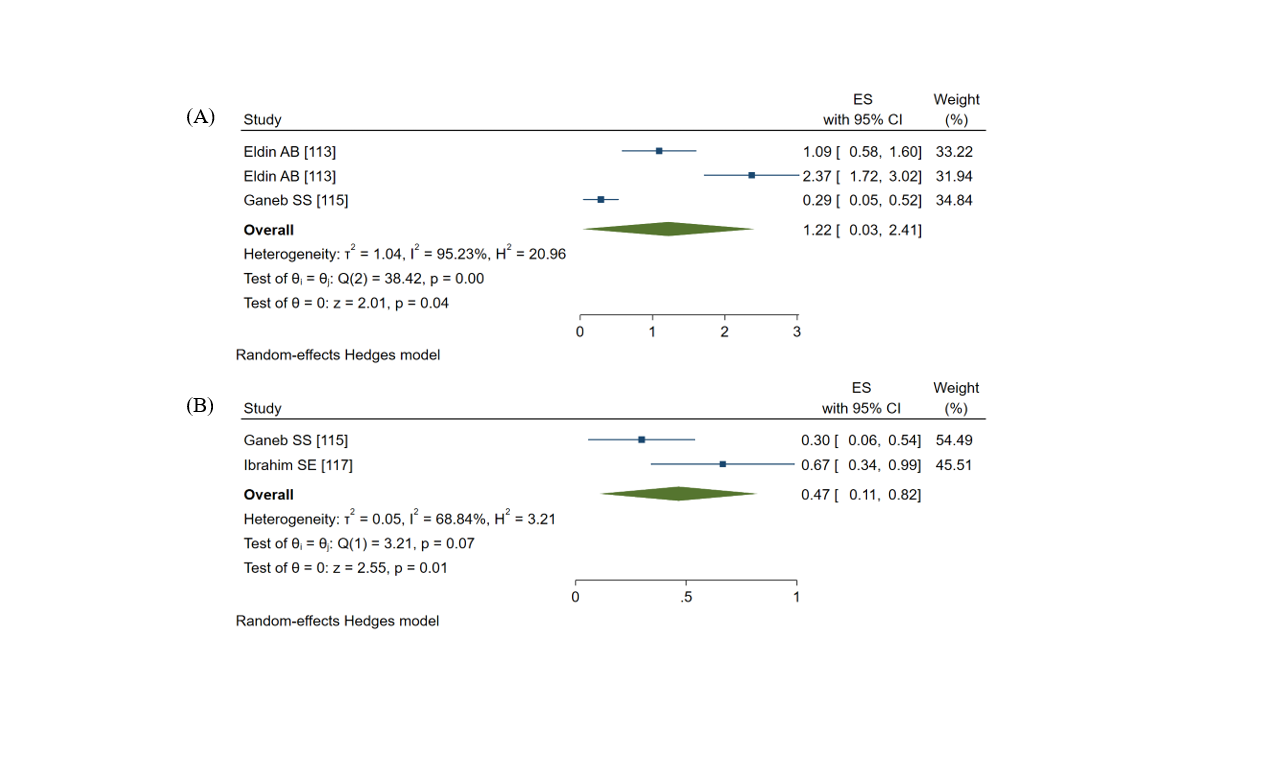


**Figure S9** Correlation analysis of BD associated with the circulating VEGF. **(A)** disease activity: BDCAF, forest plot; **(B)** ESR, forest plot. ES: fisher’s z.


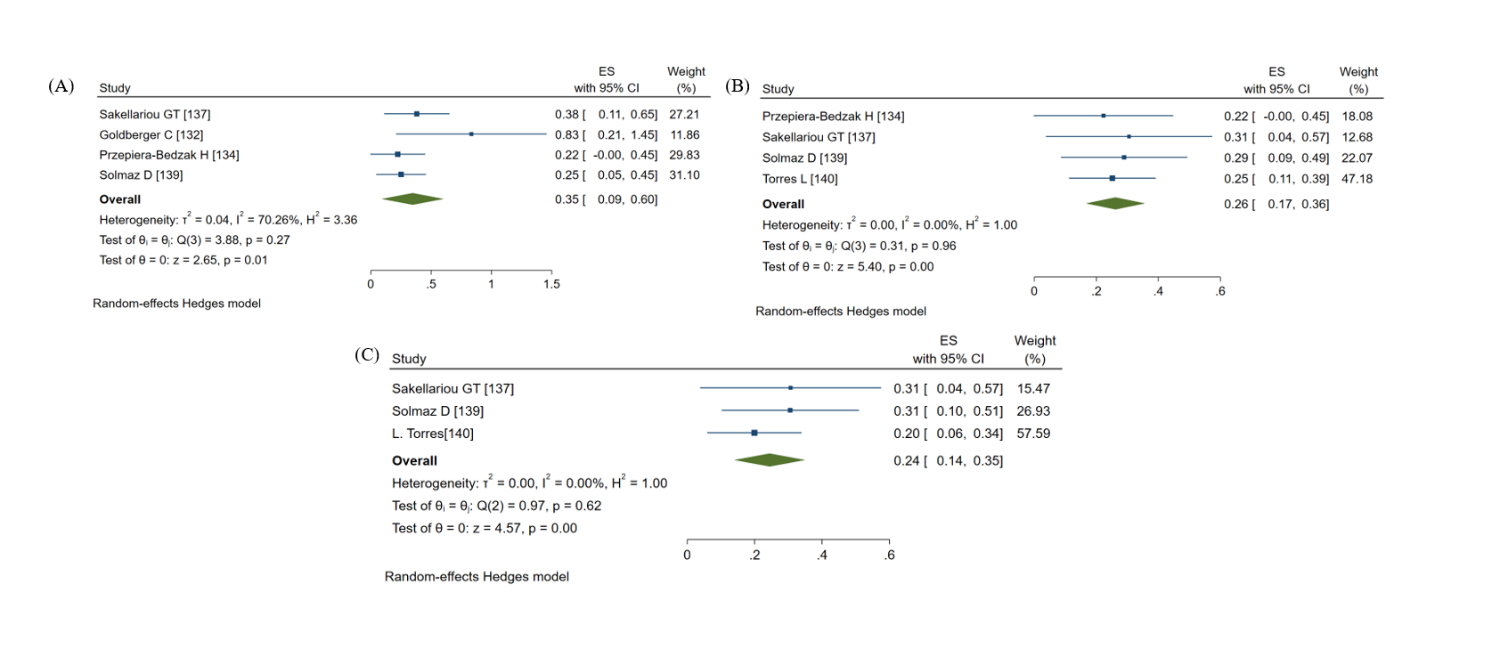


**Figure S10** Correlation analysis of AS associated with the circulating VEGF. **(A)** disease activity：BASDAI/BASMI, forest plot; **(B)** ESR, forest plot; **(C)** CRP, forest plot. ES: fisher’s z.


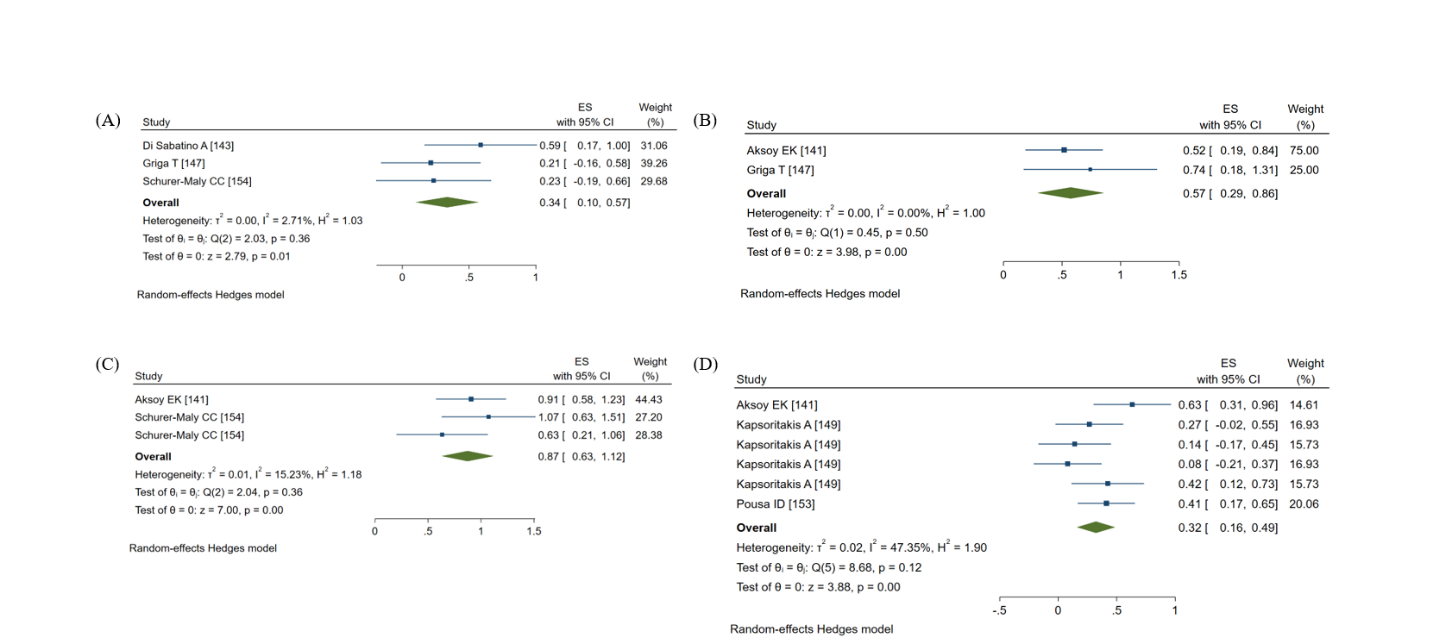


**Figure S11** Correlation analysis of IBD associated with the circulating VEGF. **(A)** disease activity: CDAI, forest plot; **(B)** disease activity: UDAI, forest plot; **(C)** ESR, forest plot; **(D)** platelet count, forest plot. ES: fisher’s z.


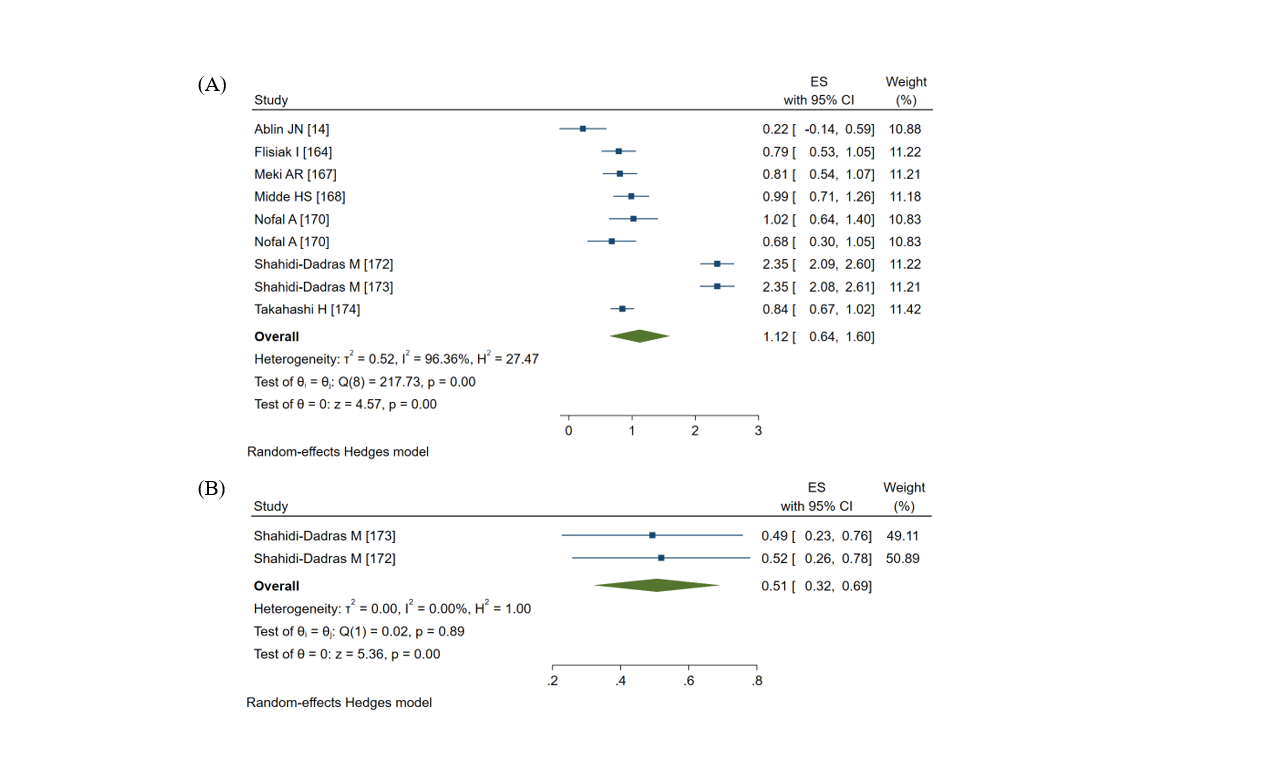


**Figure S12** Correlation analysis of PsA associated with the circulating VEGF. **(A)** disease activity: PASI, forest plot; **(B)** disease duration, forest plot. ES: fisher’s z.


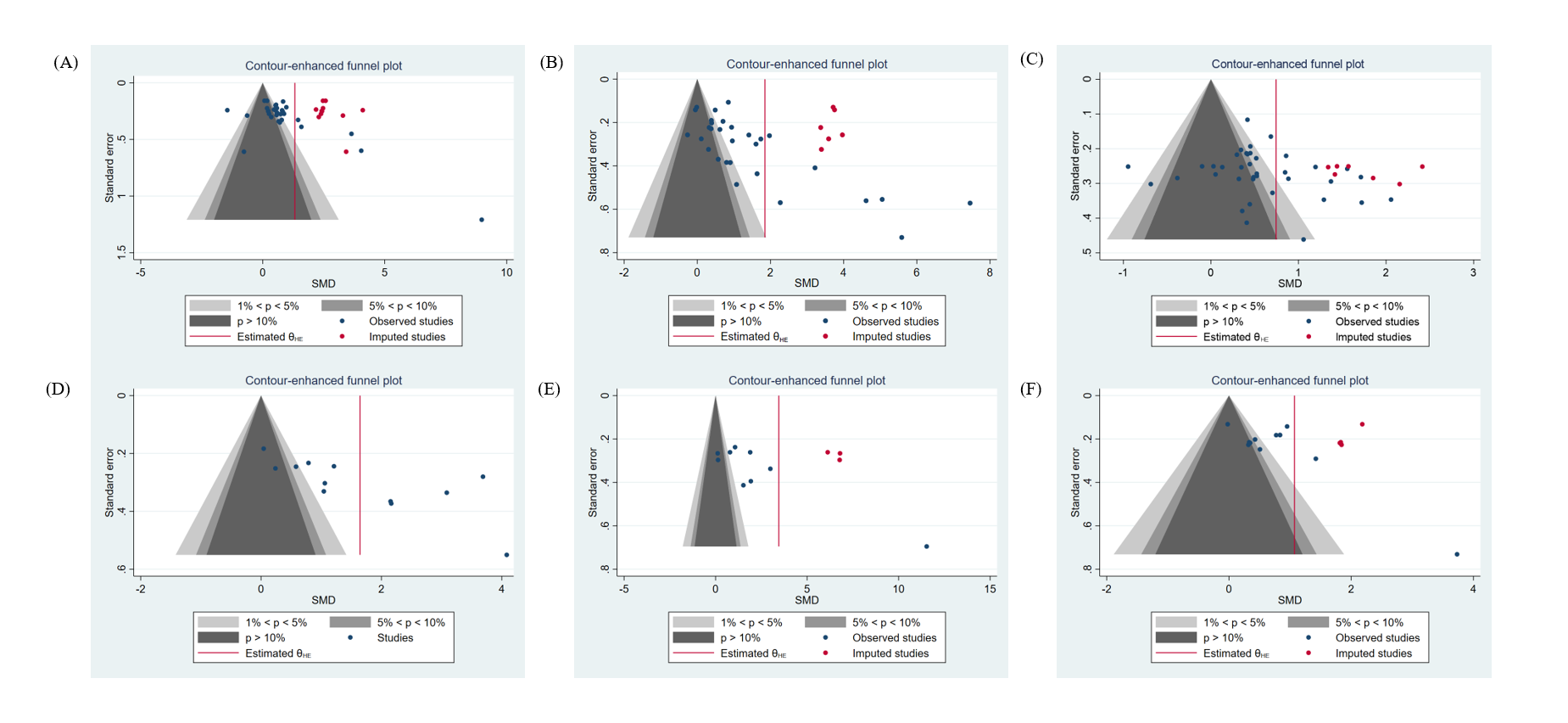


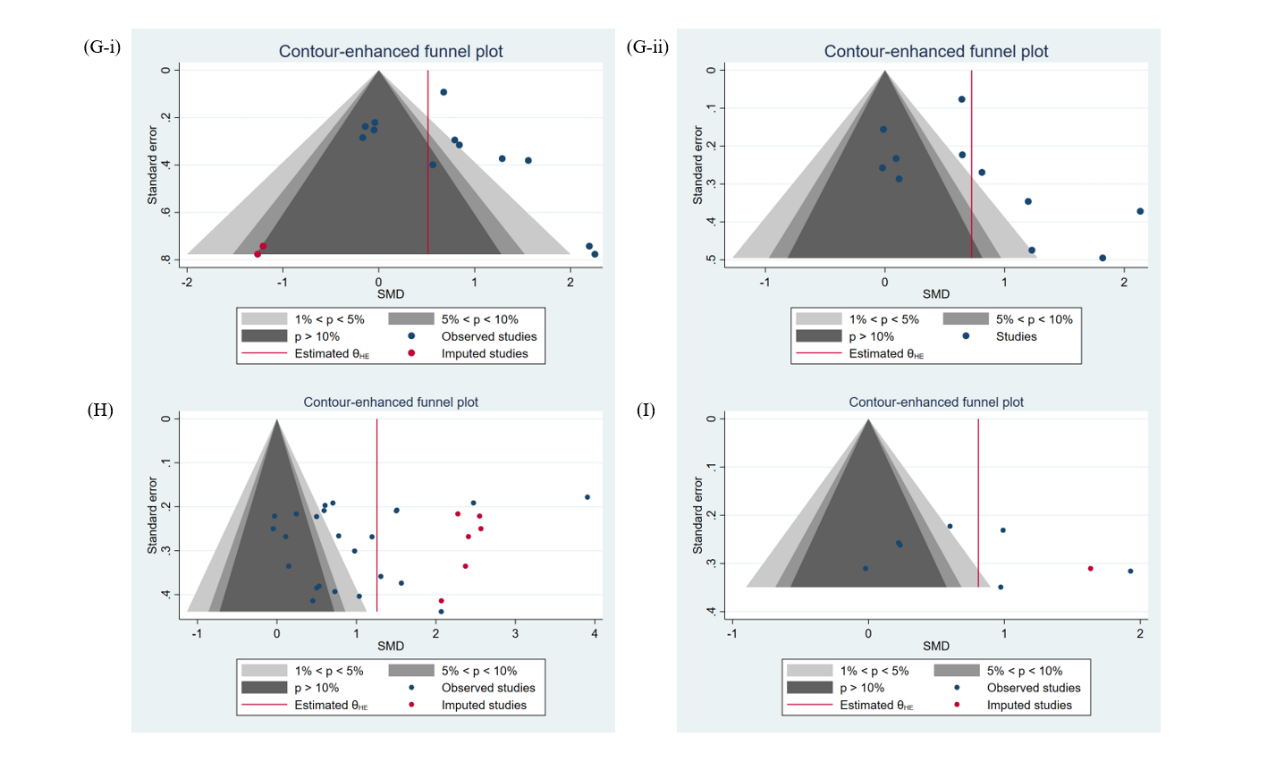


**Figure S13** Contoured-enhanced funnel plot and meta-trim analysis. **(A)** for SLE; **(B)** for RA; **(C) f**or SSc; **(D)** for BD; **(E)** for KD; **(F)** for AS; **(G-i)** for UC; **(G-ii)** for CD; **(H)** for PsA; **(I)** for GD

| **Appendix 1 Study characteristics in the meta-analysis: SLE** | | | | | |
| --- | --- | --- | --- | --- | --- |
| Year | Author | Index test method | Treatment | Diagnostic criteria | In/Exclusion criteria |
| 2015 | Barbulescu AL[20] | ELISA | Glucocorticoid; Hydroxychloroquine; Immunosuppressive agents  [n (%)]: GC 16 (88.88); HCQ 18(100); Immunosuppressive agents 14 (77.77) | (American College of Rheumatology) ACR 1982 | Control subjects were without any acute or chronic inflammatory disease, history of connective tissue disease or peripheral vascular diseases. |
| 2019 | Barraclough M [21] |  | Oral corticosteroids n (%) 12 (33.3); Average daily corticosteroid dose (mg) (n=12) median (IQR) 8.75 (6.25, 11.25); Current immunosuppressant use n (%) 15 (41.7); Current antimalarial use n (%) 22 (61.1); Biological medication n (%) 3 (8.3). | ACR 1997 /Systemic Lupus International Collaborating Clinics (SLICC) | Participants with a history of epilepsy, stroke, severe depression/psychiatric conditions or certain central nervous system (CNS)- acting medications were excluded. |
| 2008 | Ciprandi G [26] | ELISA | All SLE patients were treated with systemic glucocorticosteroids for a mean duration of 115 months. All of them had inactive SLE and were receiving therapy | SLE disease activity index score (SLEDAI) |  |
| 2009 | Colombo BM [27] | ELISA | The mean duration of corticosteroid use was 156.3 ± 124 months and all SLE patients were under prednisone therapy (＜0.5 mg/kg/bw/day) in the last month. | ACR/SLEDAI | Exclusion criteria were age＜18 or ＞65 years, pregnancy and serum creatinine level ≥3.0 mg/dL. No patient had pre-existing clinical cardiovascular or cerebrovascular events (angina, myocardial infarction, transient ischemic attack or stroke). |
| 2014 | De Jesus GR [28] |  |  | ACR | Patients with SLE (according to ACR classification criteria) followed at an outpatient clinic of rheumatology were included. They had no other autoimmune disease diagnosed and were divided according to disease activity. |
| 2015 | Ding Y [29] | ELISA |  | ACR /International Society of Nephrology/Renal Pathology Society (ISN/RPS) 2003 classification system | Patients were with SLE and biopsy-proven active LN. |
| 2009 | Elhelaly NS [30] | ELISA | Patients were not given antibiotics or any other antibacterial or antiviral medications for at least 4 weeks prior to blood collection. 20 patients had been treated with prednisone in a dose of 0.5-2mg/kg/day or every other day with a maximum dose of 60 mg/day during the corse of the disease of them only 16 patients were receiving he drug during blood sampling and 4 patients were receiving combined therapy with prednisone and cyclophosphamide. The remaining 3 SLE patients were treated with only by non-steroidal anti-inflammatory drugs. | American Rheumatism Association(ARA)/Systemic Lupus Activity Measure(SLAM) | Neither the patients or controls showed any clinical signs of infection or neoplastic disease. |
| 2012 | Edelbauer M [31] | ELISA | In the active disease group, 29% were receiving no medication, 57% were receiving corticosteroids (>10 mg per day), 43% mycophenolate mofetil (>1 g/day), 7% intravenous cyclophosphamide (750 mg/m2); among those with inactive disease, 67% were receiving corticosteroids (≤10 mg /day), 89% mycophenolate mofetil (≥1 g / d a y ) and 22% cyclosporine (>150 mg/day). | ACR/ISN/RPS 2003/ Systemic Lupus Erythematosus Disease Activity Index 2000 update (SLEDAI-2K). |  |
| 2018 | El-Gazzar II [32] | ELISA | All patients with APS were on low dose aspirin or oral anticoagulants. | Systemic Lupus International Collaborating Clinics (SLICC) /SLEDAI/Systemic Lupus International Collaborating Clinics Damage Index (SLICC DI). |  |
| 2017 | Ghazali WSW [33] | ELISA | LN group: 27 (59%) high-dose steroid medication; 17 (37%) low-dose steroid medication (prednisolone＜=10 mg/day); 2 (4%) no steroid medication. Non-LN group: 11 (24%) high-dose steroids, 23 (50%) low-dose steroids; 12 (26%) no steroid therapy.  LN group: 13 (28%) azathioprine; 33 (72%) no azathioprine. Non-LN group: 9 (20%) azathioprine; 37 (80%) no azathioprine.  LN group: 26 (57%) hydroxychloroquine. Non-LN group: 38 (83%) hydroxychloroquine.  LN group:2(4%) mycophenolate mofetil. Non-LN group: 0.  LN group: 8 (17%) pulse cyclophosphamide therapy. 1 (2%) for non-LN group. | 1997 American Rheumatism Association (ARA)/renal subscale of the British Isles Lupus Assessment Group (rBILAG, 2004)/ ACR diagnostic criteria for LN. | Patients with systemic sepsis, malignancy, diabetes mellitus, pregnancy, other autoimmune disease, SLE-like conditions such as drug induced lupus or skin lupus (who did not fulfill the 1997 ARA criteria), urinary tract infection or those who were menstruating while on haemodialysis were excluded. |
| 2007 | Heshmat NM [34] | Enzyme immunoassay (EIA) | All SLE patients were receiving oral corticosteroids in a dose ranging between 0.5 and 2 mg/ kg/day or every other day with a maximum dose of 60 mg/day. Eight patients (32%) were receiving non-steroidal anti-inflammatory drugs in the form of ibuprofen. Seven patients (28%) were receiving pulsed intravenous cyclophosphamide in a standard protocol of 600 mg/m2monthly for 7 months followed by every 3 months for an additional 30 months. | 1982 ACR/ SLEDAI | This study was conducted on children and adolescents. |
| 2009 | Hrycek A [35] | ELISA | Thirty-seven patients had already been treated (several months to several years), and 11 were newly diagnosed untreated patients in whom treatment was promptly administered. The following drugs were applied as single or in various combinations—prednisone (7.5 – 15 mg per day), immunosuppressive drugs— azathioprine (50 – 100 mg per day), cyclophosphamide (in cyclic courses 200 mg per day), cyclosporine A (50 –100 mg per day) and prolactin-suppressive drug, dopamine agonist—quinagolide (Norprolac 25 – 50mg per day). Patients were allowed to take nonsteroidal anti-inflammatory drugs and analgesics when necessary. | 1982ACR/SLEDAI | None of the patients had severe renal or hepatic dysfunction. |
| 2009 | Hrycek A [36] | ELISA | All patients had already been treated from several months to several years. In the examined persons any pulmonary toxic medications have not been applied prior to the current therapy.  Pharmacotherapy included the following drugs applied as a single or in various combinations: prednisone (5–10 mg daily), azathioprine (50–100 mg daily), cyclophosphamide (periodic administration 200 mg daily), cyclosporine A (50–100 mg daily) and prolactin-suppressive drug-dopamine agonist quinagolide (25–50 lg daily) | 1982 ARA/SLEDAI | The patients with occupational exposures for crystalline silica, solvents and pesticides were excluded. The control group without pharmacotherapy whose blood tests were within normal range. |
| 2008 | Ibrahim FF [37] | ELISA |  | 1982 ARA/SLEDAI |  |
| 1998 | Kikuchi K [38] | ELISA | No patients with SLE had received corticosteroid or immunosuppressant drugs at the time of serum sampling. |  |  |
| 2013 | Koca SS [39] | ELISA | Glucocorticoid (GC) and disease-modifying antirheumatic drug (DMARD) usages were recorded. GC dosage (mg/day) 5.6±4.2; GC usage, n (%) 15 (65.2); Azathioprine, n (%) 12 (52.2); Cyclophosphamide, n (%)1 (4.3); Hydroxychloroquine, n (%) 9 (39.1); Methotrexate, n (%) 1 (4.3); Ciclosporin, n (%) 2 (8.7). | 1982 ACR/SLEDAI/SLICC/ ACR damage index |  |
| 2007 | Kuryliszyn-Moskal A  [40] | ELISA | Twenty-one patients were treated with prednisolone at a low dosage (≤10 mg/day), 26 patients were receiving higher doses of steroids (>10 mg/day), 21 patients hydroxychloroquine, and 6 patients immunosuppressive agents (cyclophosphamide). Higher doses of steroids and immunosuppressive drugs were not used during the two months prior to blood examination. | 1982 ACR/SLEDAI |  |
| 2014 | Liu J [41] | ELISA | No patient had received corticosteroid or immunosuppressant drugs at the time of sampling. | 1982 ACR/SLEDAI | Individuals were excluded from the study if they were breastfeeding or pregnant; had a history of malignant cancer or diabetes mellitus; had a history of infections; had active central nervous system (CNS) or peripheral neurologic disease; and/or had a presence or history of vasculitis. |
| 2018 | Merayo-Chalico J [42] | ELISA | Active SLE (n, %): Immunosuppressive treatment 4 (66); Prednisone 3 (50); Prednisone dose (mean ± SEM) (mg) 26 ± 11.6 ; Cyclophosphamide 1 (16) ; Azathioprine 3 (50) ; Mycophenolate Mofetil 0 (0) . Remission SLE: none.  Patients had no immunosuppressive treatment in the 12-month period before the blood draw. | ACR/SLEDAI | Subjects were excluded with active infections or chronic infections (HIV, viral hepatitis, etc.), pregnancy, SLE associated CNS activity and the diagnosis of other concomitant autoimmune diseases. We also excluded patients with other altered laboratory parameters or image studies that could explain their neurological symptoms (i.e. serum electrolytes, thyroid function tests, CT with findings different from PRES, EEG with epileptogenic focus, cerebrospinal fluid suggestive of meningitis) or with a previous history of SLE-associated CNS activity. |
| 2016 | Novikov A [43] | Bio-Plex^®^technology (Human Grp I Cytokine 27-plex panel). |  |  |  |
| 2012 | Moneib HA [44] | ELISA | All patients were on treatment with systemic steroids, and⁄or hydroxychloroquine and topical sunscreens. | 1982 ACR/ Systemic Lupus Activity Measure (SLAM) score | Patients with diabetes, drug-induced lupus, other connective tissue diseases, lupus anticoagulants, thrombotic complications, cardiac comorbidities apart from pericarditis, manual workers and those with any injuries or traumas affecting the nail fold were excluded from the study. |
| 2002 | Navarro C [45] | ELISA |  | ACR |  |
| 2005 | Robak E [46] | ELISA | Twenty-five patients were treated with steroids and/or other immunosuppressive agents. The patients with SLE and controls showed no clinical signs of infection or neoplastic disease and received neither antibiotics nor other medications for at least 4 weeks prior to blood donation. | ARA/SLAM | The patients with SLE and controls showed no clinical signs of infection or neoplastic disease and received neither antibiotics nor other medications for at least 4 weeks prior to blood donation. |
| 2003 | Robak E [47] | ELISA | Twenty-four patients were never treated with steroids or any other immunosuppressive agents. Fourteen patients were treated with prednisone at a dose of 5mg/20 mg/day during the study and five patients with prednisone and azatioprine or cyclophosphamide. | ARA/SLAM | The patients with SLE and controls showed no clinical signs of infections or neoplastic disease and were not given antibiotics or any other antibacterial or antiviral medication for at least 4 weeks prior to blood donation. |
| 2013 | Robak E [48] | ELISA | Nineteen patients had not been treated with immunosuppressive agents.Thirty nine patients were treated with prednisoneat a dose of 5-30 mg/day during the study; five of them were additionally treated with azathioprine at a daily dose 50-150 mg. At the time of examination and collection of blood samples, the patients, as well as the control group, showed no clinical signs of infection or neoplastic disease, and had received no other medications for at least four weeks prior to blood donation. | ACR/SLEDAI-2K | At the time of examination and collection of blood samples, the patients, as well as the control group, showed no clinical signs of infection or neoplastic disease, and had received no other medications for at least four weeks prior to blood donation. |
| 2002 | Robak E [49] | ELISA | Twenty patients had never been treated with steroids or any other immunosuppressive agents. Twelve patients were treated with prednisone at a dose of 5 – 20 mg/day during the blood collection. Twenty patients were treated with prednisone and three of them also with azatioprine at some time during the course of the disease, but all of them had not been treated for at least 4 weeks before the study. | ARA/SLAM |  |
| 2017 | Willis R [50] | Luminex x-MAP | Prednisone were taking low doses (less than 10mg daily). Cohort 1:77/267 (28.8); Cohort 2:30/45 (66.7) | SELENA-SLEDAI /physician global assessment score (PGA)/BILAG index score | Patients that, at the time of testing, were receiving immunosuppressants such as azathioprine, cyclophosphamide, mycophenalate mofetil or prednisone doses above 10 mg daily were excluded as these agents may affect cytokine levels. Similarly, patients that had significant renal disease defined as a glomerular filtration rate of less than 60 mL/min/1.73 m2, which may affect vitamin D levels, were also excluded. |
| 2014 | Zhou L [51] | ELISA |  | 1982 ACR/SLEDAI | Those patients with primary vasculitis, cerebrovascular accident, primary renal disease, tumor, and any recent infections were excluded. |

| **Appendix 1 Study characteristics in the meta-analysis: RA** | | | | | |
| --- | --- | --- | --- | --- | --- |
| Year | Author | Index test method | Treatment | Diagnostic criteria | In/Exclusion criteria |
| 2004 | Ardicoglu O [52] | ELISA |  |  |  |
| 2001 | Ballara S [12] | ELISA | Patients were treated with methotrexate, sulfasalazine, or hydroxychloroquine as monotherapies or, if the disease was severe, with a combination of these agents.  Disease modifying antirheumatic drug (DMARD) therapy was initiated if clinical synovitis was observed in ＞3 joints for ＞3 months of follow up.  DMARD therapy was commenced if there were symptoms or signs of clinically active disease combined with erosions at presentation. Intramuscular steroids were given as bridge therapy for patients with polyarthralgia. Intraarticular steroids were administered to patients in whom a single inflamed joint was the dominant clinical problem | ACR/Disease Activity Score (DAS) | Patients with longstanding RA had been diagnosed for at least 3 years. |
| 2000 | Bottomley MJ [53] | ELISA |  | ACR |  |
| 2005 | Kim HR [62] | ELISA |  | 1987 ACR |  |
| 2016 | Deveci K [55] | ELISA |  |  |  |
| 2002 | Drouart M [56] | ELISA |  | 1987 ACR | The controls were healthy Caucasians with no history of inflammatory joint disease or osteoarthritis. |
| 2016 | do Prado AD [57] | Flow cytometry | Two third of patients were on oral steroids, and more than 80% were on methotrexate.  Drugs: prednisone; methotrexate; leflunomide; antimalarials; sulfasalazine.  RA patients were treated exclusively with nonbiologic disease-modifying antirheumatic drugs (DMARDs). | 1987 ACR/Disease Activity Score in 28 joints (DAS28) |  |
| 2008 | Foster W [22] | ELISA | Drug therapy (% of patients treated with this agent or class): NSAIDs 62; Methotrexate 67; Steroids 33. | ARA / DAS |  |
| 2016 | Gumus A [58] | ELISA | All of the patients were using disease-modifying anti-rheumatic drugs (a combination of methotrexate and sulfasalazine). |  | People who had predisposing factors for angiogenesis, such as hypertension, a history of smoking and other inflammatory conditions, were excluded from the study. |
| 2014 | Heard BJ [59] | Luminex | All RA patients were being managed with various immune suppressive drugs at the time of sample collection. | ACR/ X-ray changes consistent with cartilage changes within the knee joint | Inclusion criteria were an age of 40 years or older, and a diagnosis based on the American College of Rheumatology criteria with evidence of X-ray changes consistent with cartilage changes within the knee joint. All control normal participants showed no physical signs of OA or RA, and were questioned about both personal and family histories of arthritis or any autoimmune diseases. Patients were excluded from the study if they had a personal or family history of RA, systemic lupus erythematosus, systemic sclerosis, inflammatory myopathy, vasculitis, spondyloarthropathies, inflammatory bowel disease, diabetes mellitus type I and/or thyroid disease. |
| 2008 | Hetland ML [60] | ELISA |  |  |  |
| 2003 | Hashimoto N [61] | ELISA | RA patients received various therapeutic agents including nonsteroid anti-inflammatory drugs, disease-modifying antirheumatic drugs, or prednisolone (＜=10 mg/day). | ACR |  |
| 1998 | Kikuchi K [38] | ELISA | No patients with RA had received corticosteroid or immunosuppressant drugs at the time of serum sampling. |  |  |
| 2007 | Cho ML [54] | ELISA | Current prednisolone dose 4.8 ± 0.3 mg/day. | 1987 ACR |  |
| 2006 | Kuryliszyn-Moskal A [63] | ELISA | Thirty-four patients were treated with prednisolone at low dosage (<10 mg/day), 60 patients were receiving nonsteroidal anti-inflammatory drugs (NSAIDs) with or without disease-modifying anti-rheumatic drugs (DMARDs): methotrexate (MTX), 50 patients; sulphasalazine, 9 patients and intramuscular gold, 1 patient. Four patients were without medication at the time of the study. No patient had received immunosuppressant drugs at the time of sampling. | 1987 ACR |  |
| 2004 | Kuwana M [64] | ELISA | No patient with rheumatoid arthritis had received cytotoxic drugs or ciclosporin at any time during their illness, but five with rheumatoid arthritis were receiving low-dose corticosteroids (<10 mg daily) at the time of blood sampling. | ACR /evidence of active disease according to the published criteria. | No participant had diabetes mellitus or coronary-artery disease or was taking statins. Patients with symptoms that overlapped those of other connective-tissue diseases were excluded. |
| 2010 | Milman N [65] | Immunoassay biochip microarray | RA patients were treated with DMARDs (e.g. azathioprine, sulfasalazine, and methotrexate) and/or one of biologic agents (abatacept, adalimumab, anakinra, etanercept or infliximab)  Biologics (±DMARDs), N (%) 35 (74.5)  Etanercept 17; Adalimumab 13; Infliximab 3; Abatacept 1; Anakinra 1  DMARDs alone, N (%) 12 (25.5)  Methotrexate 10; Hydroxych1oroquine 10; Leflunomide 3; Sulfasalazine 1; Prednisone 1 | European League Against Rheumatism (EULAR) /DAS28 | When only patients on DMARDs were registered at the time of the recruitment, consecutive patients on DMARDs were selected. |
| 2018 | Misra S [23] | ELISA | No prior use of disease modifying antirheumatic drugs and corticosteroids. | ACR/ EULAR | All patients fulfilled the following inclusion criteria: (1) Diagnosed as RA by a rheumatologist as per ACR/ EULAR classification criteria for RA 2010.  (2) Disease activity moderate and high (DAS-28 CRP).  (3) Disease duration of less than 6 months.  (4) No prior use of disease modifying antirheumatic drugs and corticosteroids. (5) Clinical evidence of wrist joint involvement. |
| 2016 | Novikov A [43] | BioPlex^®^techn-ology |  |  |  |
| 1999 | Olszewski WL [66] | ELISA | Patients had not been treated with antirheumatic drugs for at least 3 months prior to the study, with the exception of occasional administration of nonsteroidal anti-inflammatory drugs to relieve symptoms, and had not received glucocorticoids or immunosuppressive agents for the previous 6 months. | 1987 ACR | Patients with evident cardiovascular, pulmonary, gastrointestinal, renal, or hematologic changes were excluded. Control subjects were without any history of RA who were undergoing studies of lymph lipids. |
| 2012 | Oranskiy SP [67] | ELISA | The majority of RA patients received methotrexate as a disease-modifying drug, without differences in dosages and duration of therapy between RA groups (prednisone, NSAIDs).  There were no differences in duration, dosage of corticosteroids, or NSAIDs treatment.  Current methotrexate, n (%) 30.0 (77.0); Current prednisone, n (%) 24.0 (62.0);  Current NSAIDs, n (%) 15.0 (38.0). | WHO criteria. | We excluded the patients with concomitant infectious, oncological diseases, purulent conditions of any localization, and those with renal, hepatic, or cardiac insufficiency. |
| 2010 | Ozgonenel L [68] | ELISA | Ninety percent of patients (n=36) were taking disease-modifying antirheumatic drugs (DMARDs). | ACR/DAS28 | Subjects with these comorbidities associated with neovascularization, such as hypertension, malignancy, diabetes mellitus, or pulmonary disease, smokers and pregnant subjects were excluded from the study. |
| 2009 | Young HR [69] | ELISA |  | ACR | Subjects were ＞18 years. |
| 2016 | Rodriguez-Carrio J [70] | Cytometric Bead Array Flex Set | All patients were in concomitant methotrexate and 10 (76.9 %) were also receiving low-dose glucocorticoid therapy.  None or NSAIDs 47 (22.1%); Glucocorticoids 103 (48.5%); Methotrexate 139 (65.5%); TNFα blockers 48 (22.6%); Tocilizumab 12 (5.6%); Statins 24 (11.3%) | 2010 ACR/ EULAR/DAS28) |  |
| 2016 | Smets P [71] | ELISA |  | 1987 ACR and 2010 ACR/ EULAR | We excluded patients receiving steroidal anti-inflammatory and/or immunosuppressive drugs and/or biotherapy before the determination of VEGF. |
| 2004 | Strunk J [72] | ELISA | Ten patients received no treatment, other than non-steroidal anti-inflammatory drugs at the time of examination (non-treated patients), whereas the others had already been treated with glucocorticoids and/or disease-modifying anti-rheumatic drugs over a long period (treated patients). | ACR |  |
| 2010 | Tseng JC [73] | ELISA |  | The rheumatoid arthritis patients had active disease defined as ESR328 mm/h or CRP31.0 mg/dL. |  |
| 2001 | Sone H [24] | ELISA | NSAIDs, non-steroidal anti-inflammatory drugs; DMARDs, disease modifying anti-rheumatic drugs; PSL, prednisolone).  NSAIDs (n) 120; DMARDs 111 ; PSL 70. | 1987 ACR. /radiographic examinations by Steinbroker’s criteria for determination of RA progression | Control with no evidence of joint damage or joint pain were obtained. Control samples revealing any biochemical or serological abnormality were excluded from the study. |
| 2007 | Zayed A [74] | ELISA |  | 1988ACR /Richie Articular index score |  |

| **Appendix 1 Study characteristics in the meta-analysis: SSc** | | | | | |
| --- | --- | --- | --- | --- | --- |
| Year | Author | Index test method | Treatment | Diagnostic criteria | In/Exclusion criteria |
| 2018 | Alekperov R [75] | ELISA |  | clinical examination/ pulmonary function tests /echocardiography. |  |
| 2004 | Allanore Y [76] | ELISA | Ongoing low-dose prednisone (no. patients) (mean mg/day ± SD) 14 (7.4 ± 2); Ongoing angiotensin-converting enzyme inhibitors (no. patients) 7; Ongoing low-dose aspirin therapy (no. patients) 25. Patients were asked to stop taking calcium-channel blockers 3 days before hospitalisation. | Limited/diffuse cutaneous according to the criteria of LeRoy and colleagues | The exclusion criteria were the impossibility of stopping vasodilator therapy, pregnancy, current cigarette smoking, diabetes, association with severe diseases (cardiac or hepatic failure, cancer, gangrene), and immunosuppressive therapy. A three month period of stable current treatment was required for inclusion. |
| 2013 | Aydogdu E [77] | ELISA | Drug usage was defined as every usage during the course of the disease.  Cyclophosphamide usage, n (%)  6 (15); Methotrexate usage, n (%) 10 (25); Low dose steroid usage, n (%) 22 (55); Iloprost usage, n (%) 5 (12.5); Bosentan usage, n (%) 4 (10). | ACR | Patients with a collagen tissue disease other than SSc were not included into the study. Individuals with a collagen tissue disease or a chronic disease (hypertension, diabetes mellitus, chronic renal disease, cerebrovascular disease, and coronary artery disease) were excluded from the control group. |
| 2017 | Benyamine A [78] | ELISA | Ongoing treatments in SSc patients; Number of SSc patients (%): Bosentan 9 (20.0%); Iloprost 5 (11.1%); Corticosteroid 1 (2.2%); Methotrexate 3 (6.7%); Mycophenolate Mofetil 3 (6.7%); Azathioprine 1 (2.2%); Calcium-channel blocker 20 (7.1%); Angiotensin-converting enzyme inhibitor or Angiotensin II Receptor Blocker 7 (15.6%). | 2013 ACR/EULAR/ limited cutaneous SSc or diffuse cutaneous SSc according to the criteria established by van den Hoogen et al. |  |
| 2014 | Bosello SL [79] | ELISA |  | 1987 ACR |  |
| 2014 | Bosello SL [80] | ELISA |  |  | 11 out of 24 (45.8%) SSc patients presented history of ulcers |
| 2002 | Choi JJ [11] | ELISA | Most patients had received medications including D-penicillamine, prednisolone, or colchicine, but all medications were stopped 48 h before blood sampling to minimize the effects of medications. | ACR/ dSSc or lSSc according to the criteria of LeRoy and colleagues |  |
| 2017 | Chora I [81] | ELISA | Patients were not receiving immunosuppressive medications, corticosteroids, or other disease-modifying drugs. Before blood sampling, patients were washed out for 10 days from oral vasodilating drugs and for 2 months from intravenous prostanoids. | 2013 ACR/EULAR | Inclusion criteria consisted of being classified as SSc or VEDOSS, having clinical information available for chart review (demographic, clinical manifestations, imaging, and immunology), and being able to give written informed consent for chart review and for performing blood tests. Patients with a concomitant autoimmune disease were excluded. The presence of primary RP was an exclusion criterion for healthy controls (HC). |
| 2016 | Cossu M [82] | Luminex | Use of immunosoppressants, n (%) SSC: 1 (2.1);  lcSSc 1 (13.7); dcSSc12 (34.3). | 2013 ACR/EULAR criteria and lcSSc or dcSSc according to LeRoy et al. /(UCTD/SSc) or early SSc according to LeRoy and Medsger Where skin fibrosis was present, patients were categorized as having lcSSc or dcSSc according to LeRoy et al defSSc patients according to the 2013 ACR/EULAR |  |
| 2013 | De Lauretis A [83] | Luminex | Treatment was defined as corticosteroid (prednisolone ≥ 1 mg/day) and/or immunosuppressant (cyclophosphamide, azathioprine, mycophenolate) therapy. | ACR | Patients were excluded if they had overt concurrent malignant disease, severe heart disease, and severe pulmonary hypertension associated with right heart failure. Exclusion of those with “overlap” connective tissue disorders. |
| 2017 | Delle Sedie A [84] | ELISA |  | ACR/EULAR /double-contrast esophagography/high-resolution computed tomography (HRCT) scans/pulmonary function tests (PFTs), two-dimensional echocardiogram and thoracic ultrasonography. |  |
| 2011 | Distler JHW [85] | ELISA |  | All patients fulfilled the criteria of LeRoy. | Patients fulfilling classification criteria for other connective tissue diseases were excluded. |
| 2002 | Distler O [86] | ELISA | Concomitant treatment of SSc patients included angiotensin-converting enzyme inhibitors, calcium channel blockers, proton-pump inhibitors, clebopride and topical glyceryl trinitrate.  Patients with pre-SSc were treated with calcium channel blockers and topical glyceryl trinitrate.  All patients had received therapy with intravenous prostanoids.  None of the study patients received corticosteroids, methotrexate, cyclophosphamide, D-penicillamine or other potentially disease-modifying drugs. | ACR/ dSSc or lSSc according to the criteria of LeRoy and colleagues | Patients with overlap symptoms to other connective tissue diseases were excluded from the study. All patients and controls were of Caucasian origin. |
| 2012 | Dunne JV [87] | Searchlight Custom Arrays |  | ACR/ dSSc or lSSc according to the criteria of LeRoy and colleagues | None had known coronary artery disease or diabetes mellitus. None of controls had hypertension, known coronary artery disease, diabetes or lung disease. |
| 2005 | Dziankowska-Bartkowiak B [88] | ELISA | Because of the illness course, all dSSc subjects were on disease-modifying drugs.  Cyclophosphamide (50 mg daily), prednisone (15–20 mg daily) or combination of both agents in the same doses as in monotherapies (treatment duration: 3–168 months, mean: 27 ± 48 months).  The remaining 19 patients never received the above medication. Patients from both subgroups were treated with nifedipine (10 mg daily) and vitamin E (400 mg daily) for at least 5 months. | ACR  The degree of skin sclerosis was assessed using the total skin score (TSS) according to Kahaleh et al (0–66 points). |  |
| 2006 | Dziankowska-Bartkowiak B [89] | ELISA | Concomitant treatment of SSc patients included calcium channel blockers (nifedipine 10 mg daily) and vitamin E (400 mg daily). All patients with dcSSc (n = 12), were on cyclophosphamide 50 mg daily and or prednisone 15-20 mg daily (treatment duration, median 10 months; range 7129 months) due to ILD (n = 8) and musculoskeletal involvement (n = 4). | ACR/ dSSc or lSSc according to the criteria of LeRoy and colleagues | Candidates were required to fulfil the following inclusion criteria: (1) age ≥ 18 years, (2) negative pregnancy test (women of childbearing age), and (3) disease duration ≥ 1 year.  The exclusion criteria were as follows (1) cigarette smoking; (2) pregnancy; (3) active alcohol or drug abuse; (4) presence of any coexistent chronic disease not related to SSc pathology and (5) history of any infectious disease within 3 months prior to the study. Healthy control subjects were free of any medication and fulfilled all above listed inclusion and exclusion criteria except those relating to SSc. |
| 2013 | Farouk HM [90] | ELISA |  | ACR/ dSSc or lSSc according to the criteria of LeRoy and colleagues |  |
| 2014 | Gkodkowska-Mrowka E [91] | ELISA | All patients were subjected to disease-modifying (cyclophosphamide) or symptomatic therapy, however, their disease was considered progressive. | 1980 ACR | The control group consisted of healthy volunteers without systemic hypertension and any signs of SSc. |
| 2018 | Gigante A [92] | ELISA | All SSc patients underwent treatment with calcium channel blockers (nifedipine 30 mg/day). Five patients were treated with bosentan at a dose of 125 mg twice daily. Seven SSc women were given intravenous iloprost (0.5–2 ng/kg/ min for 3 consecutive days) for healing DUs. None of the patients was treated with immunosuppressive agents (e.g. cyclophosphamide or mycophenolate mofetil or corticosteroids therapy at an equivalent dose of prednisone ⩾ 10 mg/ day), angiotensin-converting enzyme (ACE) inhibitors or angiotensin receptor antagonists, phosphodiesterase 5 inhibitors. | ACR/EULAR | Inclusion criteria were diagnosis of SSc by no less than 12 months before the study enrollment, minimum age of 18 years, sexual activity.  Exclusion criteria were menopausal status, pregnancy, nursing, psychiatric diseases, use of psychoactive drugs, congenital or iatrogenic female genital tract diseases, endocrine dysfunction not related to SSc, pulmonary arterial hypertension, scleroderma renal crisis, systemic hypertension, hyperlipidemia, intima-media thickness (IMT) >0.65 mm, cardiac and hepatic failure, diabetes, peripheral vascular diseases, and coagulopathy. |
| 2008 | Hummers LK [93] | ELISA |  | ACR/ dSSc or lSSc according to the criteria of LeRoy and colleagues |  |
| 2017 | Ibrahim SE [94] | ELISA |  | 2013 ACR/EULAR / dSSc or lSSc according to the criteria of LeRoy and colleagues | Patients with malignancy, hematological abnormality, acute or chronic infection, granulomatous chronic disease, a metabolic disease and other autoimmune diseases were excluded from the study. |
| 2018 | Kawashiri S [95] | ELISA | Five patients had been treated with low dose corticosteroids (prednisolone <10 mg daily).  None of the patients had received immunosuppressants or vasodilators such as endothelin receptor antagonists and phosphodiesterase type 5 (PDE5) inhibitors.  Ten patients treated with Calcium channel blockers against hypertension and nine patients were treated with antiagrregants. | 2013 ACR/ dSSc or lSSc according to the criteria of LeRoy and colleagues |  |
| 1998 | Kikuchi K [38] | ELISA | No patient had received corticosteroid or immunosuppressant drugs at the time of sampling.  Ten patients were receiving treatment with low dose calcium channel blocking drugs. Clinical and laboratory data were obtained at the same time as the serum samples. | ACR/ dSSc or lSSc according to the criteria of LeRoy and colleagues |  |
| 2004 | Kuryliszyn-Moskal A [96] | ELISA | Nine patients were treated with prednisolone at low dosage (<5 mg/day), 20 patients were receiving vasodilators and 7 patients were without medication at the time of the study. No patient had received immunosuppressant drugs at the time of sampling. Two patients were under calcium antagonist treatment, due to Raynaud’s disease. In these patients, before blood sampling, an adequate pharmacological washout period was observed. | ACR |  |
| 2013 | Koca SS [39] | ELISA | Glucocorticoid (GC) and disease-modifying antirheumatic drug (DMARD) usages were recorded. GC dosage (mg/day) 7.1±6.2; GC usage, n (%) 17 (45.9); Azathioprine, n (%) 9 (24.3); Cyclophosphomide, n (%) 7 (18.9); Methotrexate, n (%) 2 (5.4); Ciclosporin, n (%) 1 (2.7). | American Rheumatism Association Diagnostic and Therapeutic Criteria Committee (1980)/Valentini disease activity index/Medsger disease severity scale/Rodnan skin score (mRSS) |  |
| 2020 | Lv TT [97] | Luminex | At the sampling time, patients were not taking corticosteroids, immunosuppressive agents, or other disease modifying anti-rheumatic drugs. | 2013 ACR/EULAR/ dSSc or lSSc according to the criteria of LeRoy and colleagues | The exclusion criteria were the following: SSc patients with concomitant connective tissue diseases; patients with diabetes, pregnancy, dyslipidemia, liver disease, or malignancies; and patients who recently used drugs that might influence vasodilation. |
| 2004 | Kuwana M [64] | ELISA | No patient with systemic sclerosis had received cytotoxic drugs or ciclosporin at any time during their illness, but five patients with systemic sclerosis and five with rheumatoid arthritis were receiving low-dose corticosteroids (<10 mg daily) at the time of blood sampling. | ACR | No participant had diabetes mellitus or coronary-artery disease or was taking statins. Patients with symptoms that overlapped those of other connective-tissue diseases were excluded. |
| 2019 | Michalska-Jakubus M [98] | ELISA | All patients were on stable treatment regimen for at least 6 months, including low-dose prednisone (≤10 mg/day), vasodilators (calcium channel blockers or angiotensin-converting enzyme inhibitors), pentoxifylline (800 mg/day) and 15 of them had received pulsed intravenous cyclophosphamide (CYC) every month; however, blood samples were drawn at least 2 months after the last course of the pulsed CYC therapy. No patient was treated with prostacyclin at the time of the study and none of the patients was on endothelin receptor antagonists. | 2013 ACR/EULAR /1980 ARA | Patients with overlap syndromes, diabetes mellitus, hyperlipidaemia, thrombosis, pregnancy, neoplastic diseases and those with habitual cigarette smoking and alcohol drinking were excluded from the study. |
| 2010 | Minier T [99] | ELISA |  | Based on the criteria proposed by LeRoy et al. |  |
| 2012 | Morgiel E [100] | ELISA |  | ARA/nail-fold capillaroscopy |  |
| 2009 | Papaioannou AI [101] | ELISA | Patients who received any kind of medication for pulmonary hypertension or had a known history of arterial hypertension receiving antihypertensive drugs, except calcium channel blockers used for Raynaud's symptoms were not included in the study. | Based on the Preliminary Criteria for the Classification of Systemic Sclerosis. | Patients with a smoking history (current smokers or ex-smokers), patients with a history of coronary artery disease as well as subjects with diffuse left ventricular (LV) systolic dysfunction (Ejection Fraction <55%) or segmental abnormalities revealed by echocardiography, patients with mitral or aortic valve disorder, patients in which a satisfactory envelope of tricuspid regurgitation (TR) could not be detected by Doppler even with the use of infusion of agitated saline, patients with pattern consistent with interstitial lung disease (ILD) (i.e. subpleural opacities, parenchymal bands, thickened interlobular septae, an irregular pleural interface, honeycomb lung) in high resolution computed tomography, as those patients were prone to develop PH secondary to chronic respiratory disease were excluded. |
| 2015 | Reiseter S [102] | ELISA |  | 2013 ACR/EULAR |  |
| 2001 | Sato S [103] | ELISA | Only one patient had been treated with 15 mg/day of prednisolone. None of the patients received D-penicillamine or immunosuppressive therapy. | ACR/ according to the classification system proposed by LeRoy et al. /modified Rodnan TSS scoring technique. |  |
| 2010 | Riccieri V [104] | ELISA |  | ACR |  |
| 2017 | Saranya C [105] | ELISA | All the patients were treatment naïve, which was defined as a patient not started on any medications for SSc. | 2013 ACR/EULAR/mRSS | Patients with a history of smoking, systemic hypertension, diabetes mellitus, and valvular and coronary heart disease were excluded. SSc patients who were already on immunosuppressants and other medications were also excluded from the study. Of the total 70 SSc patients, 15 were excluded and 55 treatment-naïve SSc patients were taken as the study group. |
| 2016 | Shenavandeh S [106] | ELISA | Even patients with late SSc pattern by capillaroscopy were not receiving any previous medications. | ACR/ dSSc or lSSc according to the criteria of a proposed classification system. | Cases with overlap syndrome, impaired renal function (GFR < 60 ml/min/1.73 m2), smokers, diabetes mellitus, active infection and receiving immunosuppressives as cyclophosphamide, azathioprine, mycophenolate mofetil and methotrexate were excluded; patients using prednisolone ﹥﹦7.5 mg were also excluded from the study. healthy controls without any rheumatic diseases were also included. Patients were above 16 years and selected through random sampling. |
| 2009 | Solanilla A [107] | ELISA | Iloprost; Glucocorticoid; calcium channel blockers; Immunosuppressant; ACE inhibitor; proton pump inhibitor; anti-arrhythmic (amiodarone)；Immunosuppressant; ET-1 receptor antagonist; corticosteroids or no treatment. | ACR/ dSSc or lSSc according to the criteria of LeRoy et al. |  |
| 2016 | Yalcinkaya Y [108] | Flow Cytomix Simplex non-magnetic beads kits | No treatment 16 (22%);  CCB-PPI 55 (76%);  Acetylsalicylic acid 43 (60%);  Iloprost 3 (4%); Immunosupressives 41 (57%); Steroids(＜7.5 mg/day) 36(50%) | 2013 ACR/EULAR /mRSS (Valentini et al., 2003) /disease severity scores (Medsger et al., 2003) /NVC |  |
| 2020 | Waszczykowska A [109] | ELISA | Patients with limited systemic sclerosis received vasodilating agents (calcium channel antagonists, benzodiazepines, or angiotensin receptor antagonists, sometimes together with pentoxifylline) and vitamin E. Patients with diffuse systemic sclerosis received immunosuppressive therapy (low-dose corticosteroids-prednisone at 0.5 mg/kg bw/day) in monotherapy or in combination with a cytostatic (cyclophosphamide at 1.5 mg/kg bw/day), as well as vasodilating agents, similar to the lSSc patients. | 1980 ACR and 2013 ACR/EULAR | Controls were excluded from the interview for chronic general diseases or ophthalmologic diseases or who were taking medication. Exclusion criteria for all study participants included active infections of the anterior segment of the eye, contact lens usage, ocular surgery in the previous six months, glaucoma, allergies, and usage of local ophthalmic drugs. |
| 2008 | Wipff J [110] | ELISA |  | Cutaneous dSSc or lSSc by the criteria of LeRoy et al. | The control group were subjects with French Caucasian origin who have no autoimmune diseases. |

| **Appendix 1 Study characteristics in the meta-analysis: BD** | | | | | |
| --- | --- | --- | --- | --- | --- |
| Year | Author | Index test method | Treatment | Diagnostic criteria | In/Exclusion criteria |
| 2018 | Arica DA [111] | ELISA | A total of 32 patients were on chronic colchicine therapy, of whom 16 were receiving a minimum of one extra immunosuppressive drug (azathioprine, corticosteroid, or cyclosporine). | International Study Group | Exclusion criteria for the control group were use of any medication, signs and symptoms of any clinical infection or systemic disease, and any invasive procedure during the month before blood collection. In the patient group, individuals with a diagnosis of any chronic illness other than BD or symptoms or history of coronary artery disease, malignancy, and pregnancy were excluded. |
| 2003 | Cekmen M [112] | ELISA | None of the patients or controls had received any topical or systemic medication at least 2 weeks prior to blood collection. | International Study Group criteria/International Uveitis Study Group | Patients satisfying International Study Group criteria (International Study Group for Behçet’s Disease. Criteria for diagnosis of Behçet’s Disease) were included |
| 2013 | Eldin AB [113] | ELISA | One patient with right ventricular thrombus and RBBB was treated with oral anticoagulant, steroids, azathioprine and cyclosporine therapy with complete resolution of the thrombus within 2 months. | International Study Group / Behc¸et’s Disease Current Activity Form (BDCAF). | Patients with evidence of pre-existing cardiac and/or vascular affection due to diseases other than Behc¸et’s disease, together with cases of diabetes mellitus were excluded from this study. |
| 2003 | Erdem F [114] | ELISA | Twenty-five were taking only colchicine, and the others were receiving nonsteroidal anti-inflammatory drugs (NSAID), sulphasalazine, steroid, or interferon. | International Study Group | All subjects were nonsmokers, and those with histories of diabetes, hypertension, ischaemic heart disease, or acute or chronic neurological disorders were not included. |
| 2012 | Ganeb SS [115] | ELISA | One patient (1.4%) was on steroid use of 20 mg/d for the last one month. Thirty-one (44.3%) patients were on the colchicine therapy (ranging between 1 and 1.5 mg/d), 11 (15.7%) were also on NSAIDs together with colchicine and two (2.9%) of them were taking low-dose steroids along with colchicine. Twenty-seven (38.6%) patients were on NSAIDs only. None of the patients were on the statin therapy. | International Study Group criteria (ISGC, 1990). /BDCAF score. | Exclusion criteria: patients were excluded from the study if they had other illnesses that might affect the results of the study (or are risk factor for atherosclerosis) such as diabetes mellitus or hypertension, hyperlipidemia, alcoholics, smokers, family history of atherosclerosis, manifest cardiac diseases (infarction, stent or coronary bypass), cerebrovascular events or the statin therapy as well as patients with other rheumatological diseases. |
| 2019 | Gheita TA [116] | ELISA | The blood samples were taken before modifying the treatments in patients with activity of the disease. Oral corticosteroids n (%):  79 (82.3%) Colchicine 74 (77%) Azathioprine 56 (58.3%) Cyclophosphamide 42 (43.7%) Cyclosporine A 34 (35.4%) Methotrexate 9 (9.3%) Infliximab 13 (13.5%) Low-dose aspirin 15 (15.6%) Warfarin 34 (35.4%).  There was a significant difference between active BD patients and those in remission regarding the current oral corticosteroid dose (median of 15 mg vs 10 mg, respectively) (p = 0.035). | International Study Group criteria /BDCAF score. | Adult BD patients. Patients and controls with (1) concomitant autoimmune or autoinflammatory disease, (2) acute or chronic infection, (3) malignancy, (4) systemic disease, such as diabetes mellitus, (5) pregnancy or up to 6 months postpartum, and (6) liver, kidney, and heart failure were excluded from the study. |
| 2011 | Ibrahim SE [117] | ELISA |  | International Study Group criteria | Exclusion criteria for study and control groups included hyperlipidaemia, diabetes, hypertension, renal impairment, hepatic affection, malignancy, and thyroid diseases. |
| 2017 | Kul A [118] | ELISA | Patients who were receiving systemic agents for treatment of BD, which may potentially interfere with measurement of endocan and endothelial dysfunction are excluded | International Study Group criteria | The exclusion criteria were presence of obesity (body mass index >30 kg/m2), metabolic syndrome, diabetes mellitus, thyroid dysfunction, alcohol consumption, smoking, hyperlipidemia, hypertension, heart failure, coronary artery disease, valvular heart disease, renal failure, hepatic failure, active hepatobiliary disease, active infectious disease, malignancy and immunological disorder, patients who were receiving systemic agents for treatment of BD, which may potentially interfere with measurement of endocan and endothelial dysfunction. |
| 2009 | Ozdamar Y [119] | ELISA | After collecting the blood samples, the patients in Group A were treated with systemic steroids and immunosuppressive agents including azathioprine and/or cyclosporine A.  Nine of the patients in Group B were already receiving cyclosporine A (2–3 mg/kg/per day) at the time of the collection of blood samples, while 14 of them were not using immunosuppressives as they had no recurrences within the last 2 years. | International Study Group criteria | Patients with severe systemic manifestations of BD such as neurologic, visceral, and large vessel involvement were excluded from the study, while ones with skin lesions and oral aphtha were included if they were in inactive stage during the collection of blood sample.  Patients with other chronic diseases, including diabetes, hypertension, ischemic heart disease, neurologic disorders, hepatic or renal failure, and patients on any systemic medication (e.g., vitamins, minerals, or nonsteroidal anti-inflammatory drugs), were also excluded from the study. |
| 2007 | Ozturk MA [120] | ELISA | None of the patients had ever used statins. Eleven patients were on colchicine treatment, eight patients on low dose aspirin, and seven patients on intramuscular benzathine penicillin every 3 weeks. Four patients had been treated with steroids for less than 6 months. Four patients were taking interferon alpha, one patient azathioprine, two patients a combination of azathioprine and cyclosporine A, and one patient salazopyrine at the time of the study visit. Six patients were free of any medication. | International Study Group criteria | Subjects with diabetes mellitus, hypertension, or evidence cerebrovascular disease, and patients on longterm steroids (i.e., >6 months) were excluded from the study. Patients with active disease manifestations during blood sampling were also excluded. |
| 2018 | Sertoglu E [121] | ELISA | Most of the patients were receiving combined therapy including any two of cortisone, colchicine and azothioprine. | International Study Group criteria /BDCAF score. | Patients and controls with any known concomitant autoimmune or autoinflammatory disease, acute or chronic local/systemic infection, presence of anemia, chronic metabolic diseases (T2DM, dyslipidemia, hypertension, metabolic syndrome) and accompanying relevant concomitant medication (anti-diabetic agents, statins, hormonal replacement therapy, etc.), cardiovascular and cerebrovascular disease, any abnormality in renal or hepatic function (creatinine >1.2 mg/dL; aspartate aminotransferase and alanine transaminase more than twice the upper limit of normal), thyroid disease, malignancy, pregnancy or postpartum 6 months, and endothelial dysfunction (e.g. angiotensin-converting enzyme inhibitors and statins) and also individuals taking any medication that can potentially interfere with measurement of VEGF, sVEGFR-1 were excluded from the study. |
| 2006 | Shaker O [122] | ELISA |  | International Study Group criteria |  |
| 2013 | Yalcindag A [123] | ELISA |  | International Study Group criteria |  |

| **Appendix 1 Study characteristics in the meta-analysis: KD** | | | | | |
| --- | --- | --- | --- | --- | --- |
| Year | Author | Index test method | Treatment | Diagnostic criteria | In/Exclusion criteria |
| 2011 | Breunis WB [124] | ELISA | Serum VEGF-A was measured in acute KD prior to treatment. | Presence of fever for at least 3 days, together with at least 4 of the 5 classic diagnostic criteria or 2–3 criteria in the presence of coronary artery lesions. |  |
| 2001 | Hamamichi Y [125] | ELISA | In the acute KD group, serum levels of VEGF were evaluated before the initiation of treatment (range, 4 to 7 d; mean, 5.2 d after onset).  All KD patients were treated with i.v. gamma globulin at 200 or 400 mg/kg for 5 d as well as oral aspirin (30 mgzkg-1zd-1).  Two-dimensional echocardiography was performed before treatment with i.v. gamma globulin and at 2 wk, 4 wk, and 2 mo after the onset of KD. Time of disease onset was defined as the day on which fever appeared. | KD Research Committee |  |
| 1998 | Maeno N [126] | ELISA | All patients were treated with a high dose of gammaglobulin and aspirin within 9 d of the onset of disease, and all samples in the acute stage were obtained before the treatment. | 1984 KD Research Committee | Febrile patients with infections served as disease control subjects and patients with hypoxia were excluded from this study because it has been reported that hypoxia induces production of VEGF in endothelial cells .  All the healthy control subjects were afebrile, free of medications, and as investigations showed, normal. |
| 1999 | Ohno T [18] | ELISA | A total of 63 patients received IVGG treatment initial administration on the day 4.8± 2.1, mean±SD) of illness] with acetylsalicylic acid (30 mg/kg per day), while three received only acetylsalicylic acid because of mild symptoms. Ten patients received IVGG (400 mg/kg per day) for 3 to 6 consecutive days (until October 1994) and 53 patients received IVGG (1 g/kg per day, over 10 h) for 1 to 4 consecutive days (since November 1994). Of the 63 patients, 25 (39.7%) received at least 1.6 g/kg IVGG. | Japan Kawasaki Disease Research Committee |  |
| 2002 | Takuro Ohno [127] | ELISA | All patients received intra venous gammaglobulin (IVGG) treatment, initial administration on day 4.8±1.8 (mean±SD) of illness, with acetylsalicylic acid (30 mg/kg per day) except in two cases who received only acetylsalicylic acid because of mild symptoms. Seven patients received IVGG (400 mg/kg per day) for 3 to 6 consecutive day s (until October 1994) and 32 patient shad IVGG (1 g/kg per day, over 10 h) for 1 to 4 consecutive days (since November 1994). Of the39 patients, 17(43.6%) received at least 1.6 g/kg of IVGG. | Diagnostic guidelines |  |
| 2019 | Su Y [128] | ELISA | All blood samples were drawn before IVIG therapy in KD patients in the first week of illness. | Japan Kawasaki Disease Research Committee |  |
| 2009 | Ueno K [129] | ELISA | KS patients were all treated with IVIG 1 g/kg per day for one or two consecutive days or 2 g/kg per day for 1 day. Patients also received aspirin (30 mg/kg), and the dose of aspirin was decreased to 5 mg/kg per day after the normalisation of C-reactive protein (CRP) values. | Japanese criteria (Ayusawa et al, 2005). |  |
| 2016 | Zeng H [130] | ELISA |  |  |  |

| **Appendix 1 Study characteristics in the meta-analysis: AS** | | | | | |
| --- | --- | --- | --- | --- | --- |
| Year | Author | Index test method | Treatment | Diagnostic criteria | In/Exclusion criteria |
| 2016 | Akar S [131] | ELISA |  | BASDAI, BASFI, BASMI. /Radiographs of the pelvis, cervical and lumbar spine were scored |  |
| 2016 | Deveci K [55] | ELISA |  |  |  |
| 2002 | Goldberger C [132] | ELISA | Nonsterodial anti-inflammatory drugs(NSAIDs) and corticosteroids. | Modified New York criteria/Bath Ankylosing Spondylitis Metrology Index(BASMI) score. |  |
| 2015 | Lin TT [133] | ELISA |  | Modified New York criteria/Bath Ankylosing Spondylitis Disease Activity Index (BASDAI). |  |
| 2016 | Przepiera-Bedzak H [134] | ELISA | Patients were treated with nonsteroidal anti‑inflammatory drugs (NSAIDs) only or in combination with sulfasala‑ zine (2g/d) or methotrexate (15 mg/wk). | Modified New York criteria/ visual analogue scale (VAS). /BASDAI/BASMI/ Ankylosing Spondylitis Disease Activity Score (ASDAS) |  |
| 2015 | Przepiera-Bedzak H [135] | ELISA | 24 patients received non-steroidal anti-inflammatory drugs (NSAIDs), 26 received sulfasalazine 2 g/day, and 11 received methotrexate 15 mg/week. | Modified New York criteria/ visual analogue scale (VAS). /BASDAI |  |
| 2016 | Przepiera-Bedzak H [136] | ELISA | NSADSs with sulfasalazine 47 (58%); NSAIDS with methotrexate 9 (11.1%); NSAIDS with methotrexate and cyclosporine 0 (0%); Hypertensive drugs 25 (30.9%). | Modified New York criteria/ visual analogue scale (VAS). /BASDAI |  |
| 2016 | Sakellariou GT [137] | ELISA | Anti-TNF treatment naïve patients | Modified New York criteria/ visual analogue scale (VAS). /BASDAI/BASMI | Exclusion criteria for the recruited patients: history of osteoporosis; severe liver or kidney dysfunction; history of cardiovascular disease, diabetes mellitus or metabolic syndrome; any malignancy; previous use of biphosphonates or other anti-osteoporotic treatment; use of glucocorticoids in the previous 6 months; a high intake of non-steroidal anti-inflammatory drugs (NSAIDs) according to the recently proposed Assessment of SpA international Society (ASAS) recommendations  in the previous 3 months (a mean NSAIDs intake index ≥ 50); and use of disease modifying anti-rheumatic drugs (DMARDs) in the previous 6 months. |
| 2015 | Solmaz D [138] | ELISA |  | Modified New York criteria/ /BASDAI/BASMI / modified New York and modified Stokes ankylosing spondylitis spinal score (mSASSS). |  |
| 2018 | Solmaz D [139] | ELISA |  | Modified New York criteria/ Ankylosing spondylitis disease activity score – C-reactive protein (ASDAS-CRP)/BASDAI/mSASSS | Patients with (i) uncontrolled hypertension, (ii) severe hepatic and kidney disease (creatinine clearance <60 mL/min), (iii) severe obesity (body mass index >35 kg/m2) or (iv) malignancy were excluded. AS patients receiving TNF inhibitor (TNFi) therapy were also excluded. |
| 209 | Torres L [140] | ELISA | 76% of the patients on a tumor necrosis factor inhibitor (TNFi) were co-medicated with a conventional synthetic disease-modifying anti-rheumatic drug (csDMARD), mostly methotrexate 15 mg weekly. (Glucocorticoids 7 (3%); NSAID 158 (77%); TNFi monotherapy 10 (5%); TNFi and cDMARD in co-medication 32 (16%); csDMARD monotherapy 30 (15%). | Modified New York criteria/BASDAI/ASDAS-CRP/Bath Ankylosing Spondylitis Patient Global Score (BAS-G)/ Bath Ankylosing Spondylitis Functional Index (BASFI), /BASMI/mSASSS | Exclusion criteria were psoriasis, inflammatory bowel disease, dementia, pregnancy, and other concomitant rheumatologic diseases. Patients with language difficulties were also excluded. |
| 2010 | Tseng JC [73] | ELISA |  | Ankylosing spondylitis group patients had active disease defined as ESR28 mm/h or CRP1.0 mg/dL. |  |

| **Appendix 1 Study characteristics in the meta-analysis: IBD** | | | | | |
| --- | --- | --- | --- | --- | --- |
| Year | Author | Index test method | Treatment | Diagnostic criteria | In/Exclusion criteria |
| 2018 | Aksoy EK [141] | ELISA | Types of drugs, n (%)  ASA 32 (82.1); Steroid 8 (20.5); Metronidazole/ciprofloxacin/ ampicillin 5 (12.8); Azathioprine 5 (12.8); Cyclosporine 1 (2.6); None 5 (12.8) | UC clinical activity index (UCAI) as described by Seo et al. /Rachmilewitz endoscopic activity index (EAI) for the UC group. | Patients who presented the following conditions were excluded: chronic kidney disease; chronic liver disease; oncological diseases; pregnancy or breastfeeding; use of nonsteroidal anti-inflammatory drugs, anticoagulants or antithrombotic drugs; or previous abdominal surgery. |
| 2014 | Algaba A [142] | ELISA | IBD concomitant treatment: Oral corticosteroids 27 (73.0); Azathioprine/mercaptopurine 26 (70.3); 5-Aminosalicylic acid 12 (32.4); Budesonide 5 (13.5); Methotrexate 3 (8.1). Anti–TNF-alpha treatment was administered at standard dosages: IFX 5 mg/kg at weeks 0, 2, and 6 as induction treatment, followed by maintenance treatment consisting of 5 mg/kg intravenously every 8 weeks or ADA 160/80 mg as induction treatment at weeks 0 and 2, followed by maintenance therapy consisting of 40 mg subcutaneously every other week. The TNF-alpha inhibitor administered was IFX in 22 patients (59.5%) and ADA in 15 (40.5%). Only 2 patients (5.4%) did not proceed to maintenance therapy, one of them due to an adverse reaction to IFX infusion, and the other one because complete response of his perianal disease was achieved after 3 doses of induction treatment (one year later, this patient had to resume IFX treatment due to recurrence of perianal fistula). | IBD was diagnosed by clinical, radiological, endoscopic, and histological criteria. IBD location in accordance with Montreal’s classification/ Crohn’s disease activity index (CDAI) /modified Truelove–Witts indexes | Previous use of a TNF-alpha inhibitor was considered an exclusion criterion. Only patients with active clinical disease at baseline were included in this study. Only patients with active clinical disease at baseline included in this study. |
| 2004 | Di Sabatino A [143] | ELISA | Among the 25 Crohn’s disease patients, 18 were untreated at the time of inclusion in the study, being at the first presentation, 5 were treated with mesalazine and antibiotics and had suspended the steroid treatment at least 3 months earlier, and 2 were treated with azathioprine. | Usual clinical criteria/endoscopy, histology, and enteroclysis /CDAI | Other causes of raised levels of tissue fibrosis, such as non-alcoholic cirrhosis, alcoholic chronic liver disease, and connective tissue disorders, were screened out in all patients and control subjects. |
| 2007 | Dueñas Pousa I [144] | ELISA | 76.7% of EC patients received maintenance therapy: 20.0% aminosalycilates and 53.3% immunomodulators, including azathioprine, mercaptopurine and methotrexate. | Standard clinical, radiological, histological, and endoscopic criteria/ CDAI | All patients and controls were Caucasian. Patients receiving biologic therapies, or those presenting with cancer, coronary disorders, malignant hypertension and pregnant women were excluded from this study. |
| 2006 | Ferrante M [145] | ELISA |  | Clinical, radiologic, and endoscopic examination and histologic findings |  |
| 1999 | Griga T [146] | ELISA | Mesalamine/ Criticosteroids +mesalamine | CDAI/True love and Witss |  |
| 1998 | Griga T [147] | ELISA | Mesalamine/Criticosteroids+mesalamine. Drug treatment with glucocorticoids did not significantly affect VEGF serum levels. | CDAI/True love and Witss |  |
| 2001 | Kanazawa S [148] | ELISA | Both active UC and CD were treated with prednisolone or salazosulfapyridine. A colectomy was performed in UC cases and resection of the ileum or colon was done in CD cases. The controls did not have been taking immunosuppressants, corticosteroids, or aspirin derivatives. None had been taking immunosuppressants, corticosteroids, or aspirin derivatives. | International Organization for the Study of Inflammatory Bowel Disease | The controls were with asthma and 1with diverticulitis who had shown no evidence of inflammatory bowel disease and who had received routine total colonoscopies. None of these control patients with asthma or diverticulitis had been diagnosed with a neoplastic or inflammatory condition. |
| 2003 | Kapsoritakis A [149] | ELISA | None of the patients was under medication that may have caused platelet or coagulation abnormalities such as oral anticoagulants, aspirin, nonsteroidal anti-inflammatory drugs, and contraceptives during the last 8 weeks before blood sampling. | Radiological, endoscopic, and histological studies. /Clinical Colitis Activity Index /CDAI | Patients with abnormal liver or renal function tests, myeloproliferative disorders, or malignancies were not included in the study. |
| 2015 | Kleiner G [150] | Magnetic bead‑based multiplex immunoassay | Controls were excluded from the study if they were on any medication with a known effect on immunological factors, such as corticosteroids. |  | The study population of pediatric patients was restricted to those who were undergoing a medically indicated peripheral venous blood sampling prior to elective surgical interventions or within the scope of elective diagnostic procedures.  For the control group, subjects were excluded if they had an acute or chronic infectious disease, any clinically significant disorder, or if they were on any medication with a known effect on immunological factors, such as corticosteroids. |
| 2004 | Magro F [151] | ELISA | 5-ASA; Steroids; Azathioprine; Infliximab; or no therapy | Harvey and Bradshaw’s activity index/Truelove–Witts index |  |
| 2011 | Pousa ID [152] | ELISA | The angiogenic levels in patients were analyzed at three different intervals: before starting corticosteroid treatment (baseline), during corticosteroid treatment (1 week ± 4 days after the beginning of the treatment) and after treatment (from 1 to 5 days after finishing corticosteroid treatment). | Standard clinical, radiological, histological, and endoscopic criteria/ Truelove–Witts activity index. | Patients who were receiving biologic therapies, who presented with cancer, coronary disorders, or malignant hypertension, or who were pregnant, were excluded from the study. |
| 2007 | Pousa ID [153] | ELISA | Fifty-six CD patients (80%) were receiving maintenance therapy, 25.7% aminosalycilates and 60.0% immunosuppressors including azathioprine, mercaptopurine, and methotrexate. | Standard clinical, radiological, histological, and endoscopic criteria/CDAI | All patients and controls were white. Patients who were receiving biologic therapies, who presented with cancer, coronary disorders, or malignant hypertension, or who were pregnant were excluded from the study. |
| 1997 | Schurer-Maly CC [154] | ELISA |  | CDAI |  |
| 2020 | deZoeten EF [155] | ELISA |  |  |  |
|  |  | pediatric 17 |  | Standard clinical, radiographic, endoscopic, and histologic criteria. /Mayo score /simple clinical colitis activity index (SCCAI). | Subjects were excluded if they had known gastrointestinal infection, known causes of hypoxia, history of transplant or neutropenia.  The reasons for non-enrollment were: colitis not deemed consistent with IBD (eosinophilic colitis or mild non-specific colitis) (n=5); colonoscopy not performed (n=2); colonoscopy performed at different site (n=2); and Clostridium difficile infection (n=1). Control patients who were found to have no colitis on biopsy histology. Twelve pediatric subjects had UC, five had CD, and one had IBD unclassified. |
|  |  | adult 19 |  | Standard clinical, radiographic, endoscopic, and histologic criteria/Mayo endoscopic scoring system/Mayo score /simple clinical colitis activity index (SCCAI). |  |
| 2007 | Wiercinska-Drapalo A [156] | ELISA | Patients were treated with 5-ASA derivates in the standard dose of 3.0 g/24 h. None of them received any steroids at the time of the study. | Typical clinical and endoscopical signs of distal part bowel involvement. |  |

| **Appendix 1 Study characteristics in the meta-analysis: PsA** | | | | | |
| --- | --- | --- | --- | --- | --- |
| Year | Author | Index test method | Treatment | Diagnostic criteria | In/Exclusion criteria |
| 2009 | Ablin JN [14] | ELISA | Systemic therapy n (%) for skin psoriasis, psoriatic arthritis respectively.  Anti-TNF 0 (0), 12 (54.5);  MTX 0 (0), 7 (31.8); ACE inhibitors 1 (10), 2 (9.1); Statins1 (10), 3 (13.6); Aspirin 0 (0), 1 (4.5); NSAID’s 0 (0), 6 (27.3) | PASI score | Exclusion criteria included known cardiovascular disorders, including ischemic heart disease or prior event of CVA, diabetes and active malignancy. Demographic data were recorded as well as information regarding medication use, including anti-inXammatory agents (e.g. NSAIDS), statins etc. |
| 2007 | Akman A [157] | ELISA | Patients with psoriasis were randomly allocated to PUVA, NB-UVB or Re-PUVA treatment protocols.  The patients were treated three times a week, in Waldmann 7001-K therapy unit.  No healthy control received any phototherapy, photochemotherapy or any other systemic and topical medication.  Results of previous treatment modalities, availability for the particular treatment and patients’ medical conditions were evaluated at the baseline. | Psoriasis area and severity index (PASI) | The patients who had had any topical or systemic therapy for at least 2 months were included in the study |
| 2010 | Anderson KS [158] | Luminex Milliplex MAP multiplex assay | Patients received standard narrowband ultraviolet (UV) B therapy and peripheral blood serum samples were collected at weeks 0, 2, 4 and 6 and at follow-up, 10 weeks. Nine of 11 evaluable patients (82%) experienced an improvement in PASI of at least 75%. Patients received narrowband ultraviolet (UV) B treatment 3 days a week for 8–10 weeks. | PASI score | Patients had psoriasis vulgaris for which no treatment had been given during the past 4 weeks. None of the patients had psoriatic arthritis (PsA). Fourteen patients were included. Patients with other types of psoriasis (guttate psoriasis and psoriatic arthritis) were not include. |
| 2001 | Ballara S [12] | ELISA |  |  |  |
| 2016 | Batycka-Baran A [159] | ELISA | The patients with PsA did not receive any topical or systemic therapy at least 4 weeks prior to the initiation of the study, and had never been treated with a TNF-α antagonist. | Classification Criteria for Psoriatic Arthritis (CASPAR)/disease activity score (DAS) 28 /Moll & Wright classification / PASI. | Exclusion criteria were: history of CVD, chronic renal or liver disease, diabetes mellitus, skin disease other than psoriasis, malignancies or any significant abnormalities in blood count. |
| 2012 | Batycka-Baran A [160] | ELISA | The psoriatic patients had not received any topical or systemic therapy for at least 3 months prior to the initiation of the study. | PASI score | Exclusion criteria included: known cardiovascular disease, chronic renal or liver disease, diabetes mellitus, skin disease, malignancies or any significant abnormalities in blood count. The psoriatic patients had not received any topical or systemic therapy for at least 3 months prior to the initiation of the study. |
| 2016 | Capkin AA [161] | ELISA |  | PASI score | Patients with a history of acute coronary syndrome, cerebrovascular event, recent myocardial infarction (<3 months), heart failure, or acute severe infection were excluded. |
| 1999 | Bhushan M [162] | ELISA | None of the patients was receiving systemic or photo therapy. All topical therapies, apart from emollients, were discontinued 2 weeks prior to entry into the study | PASI score |  |
| 2002 | Creamer D [163] | ELISA |  | PASI score | Of the 22 patients (15 men and 7 women; age range, 29-77 years; mean age, 47 years) with active psoriasis in this study, 5 had GPP, 2 had erythrodermic psoriasis, and 15 had moderate-severe plaque psoriasis. |
| 2010 | Flisiak I [164] | ELISA | Blood and scales samples were collected before start of any treatment. | PASI score | Patients with other forms of psoriasis as well as persons with a history of any other inflammatory chronic disease were not included in the study. Patients with other forms of psoriasis, chronic diseases of liver, bowel, kidney, joints and any other inflammatory chronic disease were excluded from the study. |
| 2007 | Fink AM [165] | ELISA |  | Criteria for psoriasis arthritis of Vasey and Espinoza /visual analog scale (VAS)/ PASI score | Healthy patients with no history of inflammatory joint disease or osteoarthritis were recruited as controls. |
| 2012 | Kaur S [166] | Evidence Investigator TM cytokine and growth factors high-sensitivity array | None of the patients had received any systemic treatments or UV therapy in the previous 2 months, and topical therapy was stopped 5 days before blood collection. | PASI score |  |
| 2014 | Meki AR [167] | ELISA | The patients did not receive topical treatment for 1 week or systemic treatment for 1 month such as steroids, methotraxate, Psoralen Ultraviolet A (PUV A), retenoids, or cyclosporin. All patients were free of infections. | PASI score according to Fredrikson and Petterson. | Exclusion criteria were hypertension; obesity; diabetes mellitus; connective tissue diseases; and disorders of thyroid, kidney, and liver functions. |
| 2020 | Midde HS [168] | ELISA | Methotrexate monotherapy Patients with psoriasis were treated with systemic MTX, which is given orally as a tablet at a dosage of 7.5 mg/week. After 4 weeks, the dosage of MTX was slowly raised to 10 mg/week and after 8 weeks, it was increased to 15 mg/week. | PASI score | Exclusion criteria were age < 18 years, pregnancy, presence of diabetes mellitus, pre-existing atherosclerotic vascular disease, inflammatory or infectious diseases, malignancies, hepatic disease or inflammatory cutaneous disorders, and previous treatment with systemic/topical therapy. |
| 2002 | Nielsen HJ [169] | ELISA | Plasma VEGF was meassured before the treatment.  All patients were in a progressive or stable phase of the disease, and treatment with topical and/or systemic drugs and ultraviolet irradiation was stopped at least 4 weeks before study entry.  The patients were instructed that worsening might occur, and only topical indifferent emollients might be used. | PASI score | Exclusion criteria were: endocrine and autoimmune diseases, alcohol or drug abuse, pregnancy, lactation, or abnormal kidney and liver function tests. |
| 2008 | Nofal A [170] | ELISA | Blood samples were collected from patients before treatment and from controls at baseline to asses the serum levels of VEGF. | PASI score | Patients with history of hepatic, renal or cardiac insufficiency, pregnant and lactating women, and those who received topical or systemic treatment for psoriasis in the last month before enrolment were excluded from the study. |
| 2015 | Przepiera-Bedzak H [135] | ELISA | 17 patients received methotrexate 15 mg/day, 2 received methotrexate 15 mg/week in combination with cyclosporine A 3 mg/kg, 35 received sulfasalazine 2 g/day, and 15 received NSAIDs. | Caspar classification criteria/ PASI score | All PsA patients had plaque-type psoriasis. |
| 2016 | Przepiera-Bedzak H [136] | ELISA | NSAIDs only 16 (21.1%), NSADSs with sulfasalazine 31 (40.8%), NSAIDS with methotrexate 25 (32.9%), NSAIDS with methotrexate and cyclosporine 4 (5.3%), Hypertensive drugs 13 (17.1%) | Caspar classification criteria/ PASI score |  |
| 2013 | Przepiera-Bedzak H [171] | ELISA | 75 patients were treated with disease‑modifying antirheumatic drugs (37 received methotrexate, 2 received methotrexate in combination with cyclosporine A, and 36 received sulfasalazine) and 23 received only | Caspar classification criteria/ PASI score |  |
| 2016 | Shahidi-Dadras M [172] | ELISA |  | PASI score | Patients with confirmed moderate to severe chronic plaque psoriasis were recruited to our study.  Exclusion criteria included patients and controls: with classic cardiovascular risk factors; a family history of atherosclerosis; those who had overt or previous cardiovascular or cerebrovascular accidents; pregnant or lactating females; alcoholics; smokers; those who were being treated with estrogen, statins, warfarin, antiplatelet drugs, long-term systemic steroids, anti-VEGF therapies (e.g., bevacizumab) for any reason or cyclooxygenase-1 or -2 nonsteroidal anti-inflammatory drugs that can inhibit angiogenesis; patients with hepatic or cardiac insufficiency, rheumatoid arthritis, and other chronic inflammatory disorders; and those who were on systemic (for the last 6 months) or topical treatment (for the last 4 weeks) for psoriasis. |
| 2016 | Shahidi-Dadras M [173] | ELISA | Patients had received no systemic therapy or phototherapy in the last 6 months and no topical treatment apart from emollients was applied for at least 4 weeks prior to the study.  Baseline medication: Topical agents 13 (22.4%); Phototherapy 18 (31.0%); Systemic agents 27 (46.6%); Methotrexate 9 (33.3%); Cyclosporine 5 (18.5%); Acitretine 2 (7.4%); Re-PUVA 8 (29.6%); Biologic agents 3 (11.1%). | PASI score | Exclusion criteria included receiving anti-VEGF therapy for any reason such as bevacizumab; receiving COX-1 or COX-2 nonsteroidal anti-inflammatory drugs that could inhibit angiogenesis 14; a history of comorbidity with metabolic syndrome, hepatic, renal or cardiac insufficiency, rheumatoid arthritis and other chronic inflammatory disorders; pregnancy and lactation; smoking; and not achieving a PASI-75 response after 8 months of proper treatment. |
| 2009 | Takahashi H [174] | ELISA | Cases of treated, untreated, well-controlled and poorly controlled psoriasis were seen within the group, and treatments included topical steroid, topical vitamin D3, psoralen ultraviolet A and systemic treatments (etretinate, cicrosporin). Cytokine levels were measured before and after treatment, and the average treatment duration was 12 weeks. |  | The types of psoriasis seen were psoriasis vulgaris, guttate psoriasis, psoriatic arthropathica and psoriatic erythroderma. |
| 2017 | Zheng YZ [175] | ELISA | No patients had previously received medication or ultraviolet (UV A/UVB) therapy or had received drug treatment for psoriasis. | Psoriasis Lesions Area /PASI score | Inclusion criteria were listed as follows: (1) patients with psoriasis vulgaris had typical skin lesion after explicit diagnosis ; (2) patients had not received glucocorticoids or other drugs that can have an effect on body immunity function within previous month.  Exclusion criteria were listed as follows: (1) patients who had previously received medication or ultraviolet (UV A/UVB) therapy; (2) patients who had received drug treatment for psoriasis; (3) patients with autoimmune disease, allergic disease, metabolic syndrome or other serious chronic systemic diseases. |

| **Appendix 1 Study characteristics in the meta-analysis: GD** | | | | | |
| --- | --- | --- | --- | --- | --- |
| Year | Author | Index test method | Treatment | Diagnostic criteria | In/Exclusion criteria |
| 2020 | Cheng CW [10] | Bioplex Multiplex Suspension Array |  | GD and HT were diagnosed according the reported literature (Lin JD, Wang YH, Liu CH, et al. Association of IRF8 gene polymorphisms with autoimmune thyroid disease) | Blood samples of 14 women without AIDs were obtained by the Health Screening Centre of Shuang Ho Hospital from May to August 2014.  Participants were excluded if they were aged younger than 20 years, were pregnant, were alcoholic, or had a history of drug intoxication. |
| 2009 | Figueroa-Vega N [176] | ELISA | Untreated AITD patients | Patients fulfilled the diagnostic criteria for AITD/ history and physical examination, with assessment of goiter by palpation/ GO was diagnosed by complete eye examination performed / Ophthalmopathy activity was scored by using the Werners’ NOSPECS classification scheme and Clinical Activity Score (CAS) | Controls were subjects excluding the presence of thyroid disorders in all. |
| 1998 | Iitaka M [177] | ELISA | Serum VEGF were measured before and after treatment. Patients with GD were treated with antithyroid drugs. | Established clinical and laboratory criteria. | All patients with GD were hyperthyroid |
| 2014 | Kajdaniuk D [178] | ELISA | GO patients were treated with high-dose intravenous methylprednisolone pulse therapy (MP) (6 g/14 days — 1 g per day repeated within two weeks) (groups: GO before MP vs. GO after MP) and followed up by an ophthalmological assessment (two and four weeks after the beginning of MP therapy) | To evaluate eye disease, a clinical activity score (CAS) and an ophthalmopathy index (OI) were used. The active phase of GO was confirmed before MP treatment with magnetic resonance imaging (MRI) of the orbits. |  |
| 2016 | Rancier M [179] | Biochip array analyzer (Evidence) | After 6 months treatment plasma levels of VEGF. At the starting point of the discovery of the disease, GD patients were treated with synthetic antithyroid according to their status with specific doses depending on clinical parameters. | American Association of Clinical Endocrinologists and the American Thyroid Association (AACE/ATA) |  |
| 2014 | Ye X [180] | ELISA | All the patients with active GO underwent corticosteroid therapy consisting of intravenous infusions of methylprednisolone (MP) in two series of 3 g each session for 2 weeks and subsequent treatment with oral prednisone (P) at 60 mg per day for two months and then a gradual tapering schedule with a reduction of 5 mg per week by 14 weeks). All of the patients of both GO and GD groups were treated with thiamazol. | Complete eye examination, / clinical activity score (CAS) suggested by Mourits et al.. | Patients who had had prior treatment with steroids or radiation were excluded.  Healthy volunteers (control group) had neither family history of Graves’ disease nor other autoimmune diseases.  No acute infections were observed in the GO, GD, and control subjects three weeks prior to the study.  None had any other autoimmune-related disease. |
